# Metal-catalyzed phosphorylation by phosphite and energy at origins^☆^

**DOI:** 10.1016/j.bbabio.2026.149610

**Published:** 2026-09-11

**Authors:** Nadja K. Hoffmann, Manon L. Schlikker, William F. Martin

**Affiliations:** Institute of Molecular Evolution, University of Düsseldorf, 40225, Düsseldorf, Germany

**Keywords:** Phosphorylation, Phosphite, Palladium, Native metals, Hydrothermal vents, Ribose phosphate, Acetyl phosphate

## Abstract

How did phosphate become the universal energetic currency of life? Traditional approaches to phosphorylation in early evolution studies entail non-physiological conditions. Phosphite, ${\text{HPO}}_{3}^{2-}$, which is oxidized enzymatically by many microbes and which naturally occurs in serpentinizing hydrothermal vents, readily phosphorylates ribose, glucose, glycerol, serine, AMP, creatine and acetate to generate phosphoester, phosphoanhydride and acylphosphate bonds in hours to days at 25–100 ^°^C in pure alkaline water. These reactions are thermodynamically favorable because anoxic phosphite oxidation to phosphate and H_2_ is highly exergonic. But the reactions do not proceed without catalysts. The most effective catalyst yet identified is a nanoparticular form of a shiny metal: zero-valent (native, or elemental) palladium (Pd^0^). Native palladium, like phosphite, also naturally occurs in serpentinizing hydrothermal vents, as do other native platinum group elements (PGE), including Pt, Rh, Ru and Ir. Here we survey the use of standard currencies of biochemical energy used in core biosynthesis of amino acids, bases and cofactors from H_2_, CO_2_, NH_3_, H_2_S, H_2_O and P_i_, their frequency is phosphate > redox > thioesters. We test PGE as catalysts of phosphite oxidation and phosphorylation. Although all metals tested readily oxidize phosphite, only Pd^0^ efficiently catalyzes phosphorylation, generating phosphorylated products at physiologically relevant concentrations. In phosphorylation reactions via phosphite oxidation, a portion of the energy released is conserved in phosphorylated products, as in biological energy conservation. Favorable thermodynamics of aqueous phosphite-dependent phosphorylation reactions suggest their possible involvement in the origin of metabolism.

## 1 Introduction

Life is an energy-releasing chemical reaction. Its catalysts harness sources of environmental energy using information stored in genes. Since its beginning ~3.8 billion years ago [1,2], life has always required phosphate for energetics and information [3]. Quantitatively, the main function of phosphate in life is its energetic role in ATP. During growth, *Escherichia coli* hydrolyses ~10^10^ phosphate bonds in ATP [4] to make a cell that contains only ~10^8^ phosphate residues [5]. In free solution under standard physiological conditions (1 M concentrations of reactants, pH 7 and 25 ^°^C; Δ*G*_0_′) the ATP hydrolysis reaction to ADP and phosphate is exergonic by – 32 kJ·mol^−1^. Given suitable catalysts at pH 7 and 25 ^°^C, the ATP/ADP ratio at equilibrium is 10^−7^/1 [6]. In growing cells, the ATP hydrolysis reaction is more exergonic than under standard physiological conditions because in growing cells ATP is present at higher concentrations than ADP, counter to their concentrations at equilibrium, and both metabolites are present in roughly millimolar, not molar, concentrations [7]. In the cytosol of *E. coli* growing on glucose without electron acceptors, for example, the ATP/ADP ratio is 7.6/1 with Δ*G* for ATP hydrolysis to ADP and phosphate at 37 ^°^C being −46.5 kJ·mol^−1^ [8]. In the matrix of respiring mammalian mitochondria, Δ*G* for ATP hydrolysis is −47 kJ·mol^−1^ [6]. For the slightly alkaline cytosol of *Streptococcus faecalis*, Δ*G* for ATP hydrolysis is −50.2 kJ·mol^−1^ [7].

The main reason why ATP hydrolysis is thermodynamically more favorable in cells versus the reaction in free solution is the constant activity of the rotor stator ATP synthase, which keeps the ATP hydrolysis reaction far from equilibrium by harnessing ion gradients generated during the redox reactions of energy metabolism. This familiar configuration—a rotor-stator ATP synthase [9] that harnesses chemiosmotic ion gradients [10] to constantly synthesize ATP from ADP and phosphate—is the backbone of bioenergetics in modern life. The ATP synthase is as universal as the genetic code and is older than the machinery that the physiologically most primitive archaea (methanogens) and bacteria (acetogens) use to couple redox reactions [11,12] to the pumping of sodium ions from the inside of the cell to the outside. Ever since the very first cells on Earth arose, the archetypal bioenergetic circuit has gone unchanged: Redox reactions that power ion pumps that generate ion gradients that power ATP synthases [13]. That conservation traces the bioenergetic circuit to the first free-living cells [14].

What sources of energy drove organic synthesis before there were free-living cells? Bioenergetics based on displacement from equilibrium and ATP synthase activity operate in cells, which are *closed* systems [6,7] such that equilibrium thermodynamics apply [6,7]. As it concerns energy at origins [3,14–24], the topic of this paper, the closed system of the free-living cell represents the endpoint of early (precellular) evolution [14–20]. We are concerned here with processes that preceded the origins of cells, where evolution took place in *open* systems, which freely exchange energy and material with the environment [6]. The ATP synthase is synthesized by ribosomes and in any sensible account of early evolution, ribosomes arose before free-living cells. Ribosomes arose before the proteins that they synthesize today [20] and the RNA component of the ribosome is, in turn, 29% phosphate by weight. This brings us back to Westheimer’s [3] question of how and why phosphate came to play a role in energy and information before there were cells whose energetics were governed by equilibrium thermodynamics.

Protein structural data are now permitting insights into the nature of ancient bioenergetic processes that existed before there were free-living bacteria and archaea [15]. A case can be made that GTP preceded ATP in evolution [20], but that only begs the question of what came before GTP, the source of energy that powered amino acid synthesis required for the very first proteins, and the source of energy that reduced CO_2_ to organic compounds in the first place? Clearly, the chemical reactions of life, particularly at origins, require sources of energy [14–26]. It is possible that relics of ancestral energy currencies are preserved in metabolism. Many essential and highly conserved reactions of cells depend upon phosphate, all the way down to the synthesis of phosphorylated nucleosides and cofactors, and their prebiotic (non-enzymatic) forerunners. Here we survey the currencies of energy that are used in the conserved core of biosynthetic metabolism for evidence of chemical energy sources used at origins and newer findings on phosphite oxidation that impact the possible nature of non-enzymatically synthesized geochemical energy currencies.

## 2 Material and methods

### 2.1 Energy currencies in metabolism

**Fig. 1:**
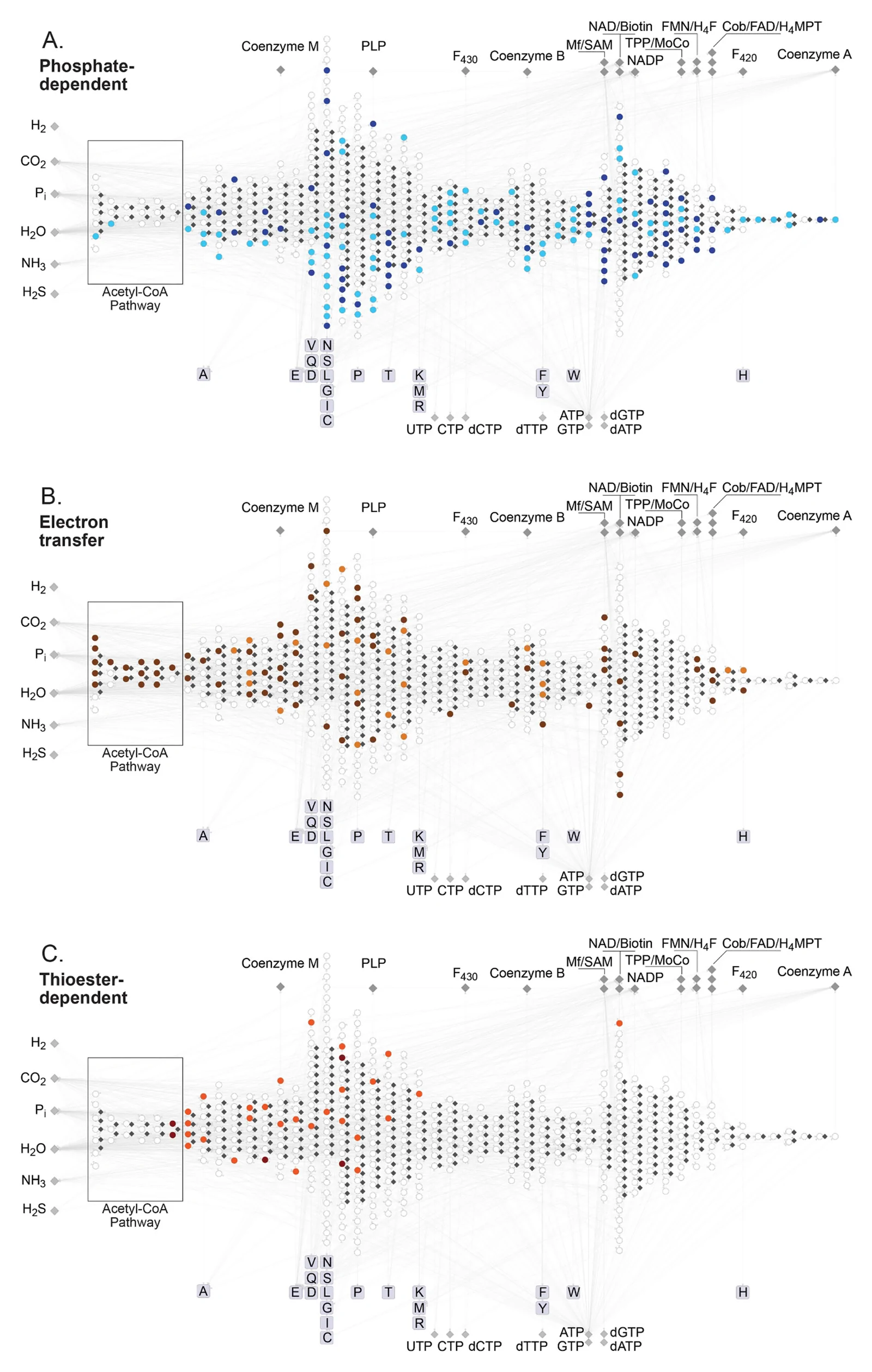
Energy in metabolism. Vectorial representation of metabolism highlighting the roles of phosphate, electron transfer, and thioester-dependent reactions. Compounds are represented as diamonds and connected by reactions represented as circles. The starting compounds of metabolism are indicated on the left: H_2_, CO_2_, Pi, H_2_O, NH_3_, and H_2_S. (**A**) Phosphate-related reactions are color-coded in dark blue for phosphorus bond formation or cleavage and in light blue for reactions in which ATP, other NTPs, or NDPs are used as cofactors. (**B**) Electron transfer reactions are shown in light brown for oxidations and in dark brown for the remaining electron transfer processes. (**C**) Thioester-dependent reactions are colored orange when a thioester is consumed and dark red when a thioester is formed. (For interpretation of the references to color in this figure legend, the reader is referred to the web version of this article.)

The set of 420 core biosynthetic reactions (see Table S1) described by Mrnjavac et al. (2026) [15], encoded as tabulated reactions, were converted into a network using Cytoscape (Version 3.10.3). Reactions, represented as circles in Fig. 1, were color-coded based on their functional categories or energy currencies (see Tables S2–S4). The resulting graphs were edited using Affinity Designer (Version 2.6.3).

### 2.2 Phosphite oxidation

Reactions contained 200 mM sodium phosphite (NaH_2_PO_3_; Merck, Sigma-Aldrich) dissolved in distilled water and adjusted to a final volume of 1.5 mL. Where indicated, serine (100 mM; Carl Roth) or adenosine monophosphate (AMP, 100 mM; Merck, Sigma-Aldrich) was added. Catalysts were purchased from Merck, Sigma-Aldrich, unless stated otherwise. Metals (Pt, Pt/Al_2_O_3_, Ir, Rh, Rh/Al_2_O_3_, Ru, Ru/C, Ru/ Al_2_O_3_, Pd, Pd/C, Ni and Ni/C) were weighed out under ambient laboratory conditions and added at a loading of 1.5 mmol of metal per reaction. Supported catalysts, Ru/C (10 wt% Ru on activated carbon) and Pd/C (10 wt% Pd on activated carbon), were added at amounts corresponding to approximately 0.15 mmol of elemental metal per reaction. Catalyst loadings are reported as mmol of elemental metal. Reaction mixtures were transferred into 3 mL glass vials, sealed with screw caps, and punctured with a sterile needle to allow pressure equilibration while maintaining a closed reaction environment. The vials were placed in a stainless-steel pressure reactor equipped with a temperature controller (Berghof BR-300 with BTC-3000). The reactor was purged three times with argon and subsequently pressurized to 5 bar Ar (99.999%). Reactions were conducted at 100 ^°^C for 72 h. After completion, the reactor was depressurized, and the vials were immediately transferred to 2 mL microcentrifuge tubes. Samples were centrifuged for 20 min at 16,060 × *g* to separate the catalyst from the supernatant. The supernatants were collected for NMR analysis as described [15,26].

## 3 Results

### 3.1 Energy at origins: querying metabolism

Evidence for the nature of energy at origins should be preserved within metabolism itself [23,27]. What is metabolism? In some views, metabolism is everything that is not RNA replication [28]. In other views it is all 12,000 reactions that are included in the Kyoto Encyclopedia of Genes and Genomes database [29,30]. In our approach to origins, metabolism is the reaction set that converts microbial growth substrates present on the early Earth into the building blocks of life [15,31]. That discrete reaction set connects microbial physiology to the early Earth and was a requirement for the growth of the first cells. The 420 reactions that convert H_2_, CO_2_, NH_3_, H_2_O, H_2_S, and phosphate [15] into the 20 standard amino acids, the 8 (unmodified) bases of DNA and RNA and 18 cofactors used by chemolithoautotrophs today are shown in Fig. 1. Because these reactions are conserved across prokaryotic lineages, they trace to LUCA, whereby only about half of the corresponding enzymes do, with environmental catalysts preceding enzymes at many reactions [15].

The depiction of metabolism in Fig. 1 differs from standard biochemical pathway maps in that the reactions of metabolism are ordered from left to right in columns. A reaction is plotted in a column (a stratum: [15]) only if its educts are present in the preceding column: either present in the environment (the food set for the network [15]) or generated by reactions in preceding columns. This ordering of metabolic reactions starts with environmental substrates and generates increasingly complex compounds from left to right, the corresponding algorithm is called stratification, developed by Mike Steel and Daniel Huson (for details, see [15]). It polarizes metabolism in the anabolic direction employed by chemolithoautotrophs. Metabolism starts from H_2_ and CO_2_ via the acetyl-CoA pathway, indicated in the figure, the most ancient pathway of CO_2_ fixation, the only pathway of CO_2_ fixation that operates in bacteria and archaea [32,33] and the only pathway of CO_2_ fixation that goes forward in the laboratory without enzymes—native metals catalyze the synthesis of pyruvate from H_2_ and CO_2_ overnight in water under the conditions of serpentinizing hydrothermal vents [34–38]. The entire set of enzymes and cofactors of the acetyl-CoA pathway from H_2_ and CO_2_ to pyruvate can be replaced by a simple piece of native metal, Ni^0^ or Ni_3_Fe [34], metals that are naturally deposited in serpentinizing hydrothermal vents [39]. Enzymes do not make impossible reactions possible, they just accelerate reactions that tend to occur anyway [40]. The Ni^0^-catalyzed acetyl-CoA pathway is arguably the precursor of the enzymatic pathway [32]. Metabolism speaks to sources of energy at origins.

### 3.2 Phosphate

Phosphate bonds are the most widely discussed currency of energy in metabolism [3,41]. Fig. 1A shows the 420 reactions of metabolism, highlighting those 149 reactions (35%) that involve the formation or cleavage of a phosphate bond. In 67 reactions (16%), a phosphoanhydride bond, usually in ATP, is cleaved. Those 67 reactions, 11 of which are adenylylations in cofactor synthesis, are highlighted in dark blue. There are 82 other reactions in which a phosphate bond is otherwise formed or cleaved (highlighted in light blue). Among the 420 reactions, 271 (65%) involve phosphorylated compounds, indicating that phosphate is hard-wired into the reactions of metabolism and is a main source of energy for the network that produces the amino acids of which its enzymatic catalysts are composed. Contrary to some views [29], Fig. 1A indicates that no reaction network approaching the organization and structure of metabolism as defined by amino acid, base, and cofactor biosynthesis can have formed or existed without phosphorylation. As a case in point, the enzymatic biosynthesis of ATP in *Methanococcus thermolithotrophicus* from the starting compounds in Fig. 1 requires 15 ATP: five to phosphoribosyl pyrophosphate [42], eight more to AMP as in *E. coli* [15], and two more to ATP. Was ATP the source of primordial phosphorylation for its own synthesis before there were enzymes, or did simpler phosphorylating compounds precede ATP? That will be the topic of a later section.

### 3.3 Redox

Behind phosphate, the next most common energy currency in metabolism is electrons (reductions). Reduced ferredoxins are an important currency of energy conservation in anaerobes [43–45]. The starting point of metabolism, the acetyl-CoA pathway, requires low potential ferredoxins with a midpoint potential on the order of *E*_0_′ = −450 mV [43–46]. How methanogens and acetogens reduce low potential ferredoxin with electrons from H_2_ (*E*_0_′ = −414 mV) was not clarified until the discovery of flavin based electron bifurcation [43–45]. Under conditions of serpentinizing hydrothermal vents, Fe^0^ can reduce low potential ferredoxin with H_2_, functionally substituting for flavin based electron bifurcation [47]. In Fig. 1B, all redox reactions of metabolism are highlighted in brown. Highlighted in dark brown are the 72 reactions (17%) in which a carbon substrate is reduced, 38 reactions using NAD(*P*)H. NAD(*P*)H is a moderately strong reductant (*E*_0_′= − 320 mV), such that NAD(*P*)H-dependent reductions are often exergonic. For example, using the Nernst equation, the NADH-dependent reduction of pyruvate to lactate (*E*_0_′ = −190 mV) is exergonic with Δ*G*_0_′ = −25 kJ·mol^−1^ under standard physiological conditions. Light brown are the 24 reactions (6%) in which the carbon substrate is oxidized, usually with NAD(P)^+^. In the cytosol of growing *E. coli*, the ratio of NAD^+^/NADH is 31/1, while the ratio of NADPH to NADP^+^ is 57/1 [48]. In core metabolism, reactions involving reductions with NAD(*P*)H (17%) are just as frequent as reactions involving hydrolysis of phosphoanhydride bonds in ATP (16%).

The role of redox in the acetyl-CoA pathway (Fig. 1B) is noteworthy. Though there are only six reactions in metabolism that require reduced ferredoxin, five are required in the acetyl-CoA pathway to pyruvate. In acetogens, only one reaction of the pathway is ATP-dependent, formyltetrahydrofolate synthase [45], and that ATP investment is regained from chemiosmotic energy coupling at the Rnf reaction, which pumps Na^+^ during the exergonic reduction of NAD^+^ with reduced ferredoxin [12,45]. In methanogens, none of the reactions from H_2_ and CO_2_ to pyruvate require ATP. Laboratory synthesis of pyruvate from H_2_ and CO_2_ using native metals as catalysts goes forward because the reactions are exergonic under the conditions of serpentinizing hydrothermal vents [34]. In the absence of enzymes, native metals catalyze the H_2_-dependent reduction of ferredoxin [47], the H_2_-dependent reduction of NAD^+^ [49,50], the reduction of FAD [51], and can replace both enzymes and NAD(P)H in >12 different reactions of metabolism [52].

### 3.4 Thioesters

Thioesters are commonly regarded as an important primordial energy currency [53]. There are 32 reactions of metabolism in which thioesters are involved, these are highlighted in Fig. 1C. Only five reactions of metabolism generate thioesters from thiols (marked dark yellow in Fig. 1C): the acetyl-CoA synthase reactions in the acetyl-CoA pathway, succinyl-CoA synthetase, citryl-CoA synthetase, and ATP citrate lyase. The first two are redox-dependent using CO as reductant, the latter three are ATP-dependent. The remaining 27 involve thioester hydrolysis, usually acetyl-CoA, of which eight are involved in biotin synthesis, and nine are involved in amino acid biosynthesis (a complete list of reactions is given in Table S1). The main source of thioesters in organisms that use the acetyl-CoA pathway is the CO-dependent synthesis of a Ni-bound acetyl moiety at the active site of acetyl-CoA synthase that is removed from the enzyme by thiolysis with CoASH [54]. There is currently no known environmental source of abiotically synthesized thioesters or thioacids, and both have low environmental stability [55,56].

Among 420 reactions of metabolism, 149 involve phosphate bonds, 72 involve electron transfer (redox), and 32 involve thioesters. Thioesters are a currency of energy conservation, but they are generated endogenously by metabolism, rather than being an environmental source of energy and they are not environmentally stable [56]. That stands in contrast to H_2_, which is an environmental source of energy and electrons that is continuously produced by geochemical reactions in serpentinizing hydrothermal vents [18,39,46,57–60]. Is there an environmental source of energy for phosphorylation [41], similar to the case for H_2_-dependent reductions, that could have served as a phosphorylating agent prior to the origin of enzymatic phosphorylation? Findings from the study of anaerobic bacteria provide important clues.

### 3.5 Enzymatic and metal-catalyzed phosphite dependent phosphorylation

The acetyl-CoA pathway used by anaerobic bacteria and archaea served as a guide to identify metals that catalyze primordial entry of carbon into metabolism [32,34]. In the same way, phosphite oxidation in anaerobic bacteria served as a guide to identify metals that catalyze primordial entry of phosphate into metabolism [26]. *Phosphitispora fastidiosa* is a Gram-positive bacterium that uses the acetyl-CoA pathway for CO_2_ fixation. It can grow on CO_2_ as an acetogen, but it can use phosphite, ${\text{HPO}}_{3}^{2-}$, which it oxidizes to phosphate, as the sole electron source [61] instead of H_2_. A surprisingly large number of bacteria can use phosphite as their sole source of phosphorus [62,63], evidence that phosphite naturally occurs in the environment. Phosphite-oxidizing genes are enriched in microbiomes from serpentinizing hydrothermal vents [18,64,65], indicating that phosphite is present in serpentinizing environments, and limited environmental sampling has shown that serpentinizing systems contain phosphite [66], as did Archaean oceans [67]. Bernhard Schink and colleagues recently showed that *P. fastidiosa* performs substrate-level phosphorylation in the phosphite oxidizing enzymatic reaction, converting AMP to ADP in a hitherto unprecedented example of a redox-dependent substrate-level phosphorylation reaction [61,68].

The occurrence of phosphite in serpentinizing systems is noteworthy. Serpentinization is a geochemical process that entails circulation of deep-sea water through the Earth’s crust and chemical reactions of water with Fe(II)-rich minerals (rock-water interactions) that generate highly reducing conditions (pH 9–11) and H_2_ gas as diffusible reductant [57–60]. The midpoint potential of the phosphite–phosphate redox couple is −690 mV [69]. Because of high alkalinity (pH often >10) and high H_2_ concentrations, the midpoint potential of effluent in serpentinizing systems can exceed −900 mV [46,70] because alkalinity pulls the equilibrium in the H_2_ oxidizing reaction, H_2_ → 2e^−^ + 2H^+^, to the right [18]. These highly reducing conditions lead to the formation and deposition of shiny transition metals (synonymous with native metals, zero-valent metals, elemental metals), mainly Ni^0^, Fe^0^, Co^0^, Ni_3_Fe alloys, CoFe alloys [39], but including the less common platinum group elements (PGE), Pd^0^, Pt^0^, Rh^0^, Ru^0^, Ir^0^ and Os [71]. Previous work has shown that Ni^0^, Co^0^, Fe^0^ and their alloys serve as effective catalysts for H_2_-dependent CO_2_ fixation in reactions that generate products of the acetyl-CoA pathway [33]: formate, acetate, pyruvate and methane with high specificity under aqueous conditions of serpentinizing vents [34–38]. The reactions generate up to 200 mM formate and up to 200 μM pyruvate [35], equal to the physiological concentration of pyruvate in cells that use that acetyl-CoA pathway for carbon and energy metabolism [72]. The native metals not only replace the enzymes of the acetyl-CoA pathway, which have Fe, Ni and Co catalysis at their active sites [73–76]. They replace enzymes of hydrogen oxidation, amino acid synthesis [77], the reverse citric acid cycle [77,78], hydrocarbon synthesis, C1 transfers [33–38], pyridoxal metabolism [79], NAD^+^, ferredoxin and FAD reduction [47,49–51] among others [15,32,52,80]. These native metals replace many enzymes that have no metal cofactor and they replace not just enzymes, they also replace cofactors [15,52].

Because native metals exhibit broad specificity for reactions and substrates, and because both native metals and phosphite naturally occur in serpentinizing systems, we asked whether native metals could also replace the enzyme AdpA, which catalyzes the phosphite-dependent, NADH-forming substrate-level phosphorylation reaction:

$$\text{AMP}+{{\text{HPO}}_{3}}^{2-}+{\text{NAD}}^{+}\to \text{ADP}+\text{NADH}$$

in *Phosphitispora fastidiosa* [68]. We found that Ni^0^ catalyzes the oxidation of phosphite to phosphate and replaces the enzyme in the AdpA reaction, also replacing NADH with H_2_ [15,26]. But we also found that Pd^0^, directly below Ni^0^ in the periodic table, is a far more effective catalyst of the same reaction:

$$\text{AMP}+{{\text{HPO}}_{3}}^{2-}\to \text{ADP}+{\text{H}}_{2}.$$

**Fig. 2:**
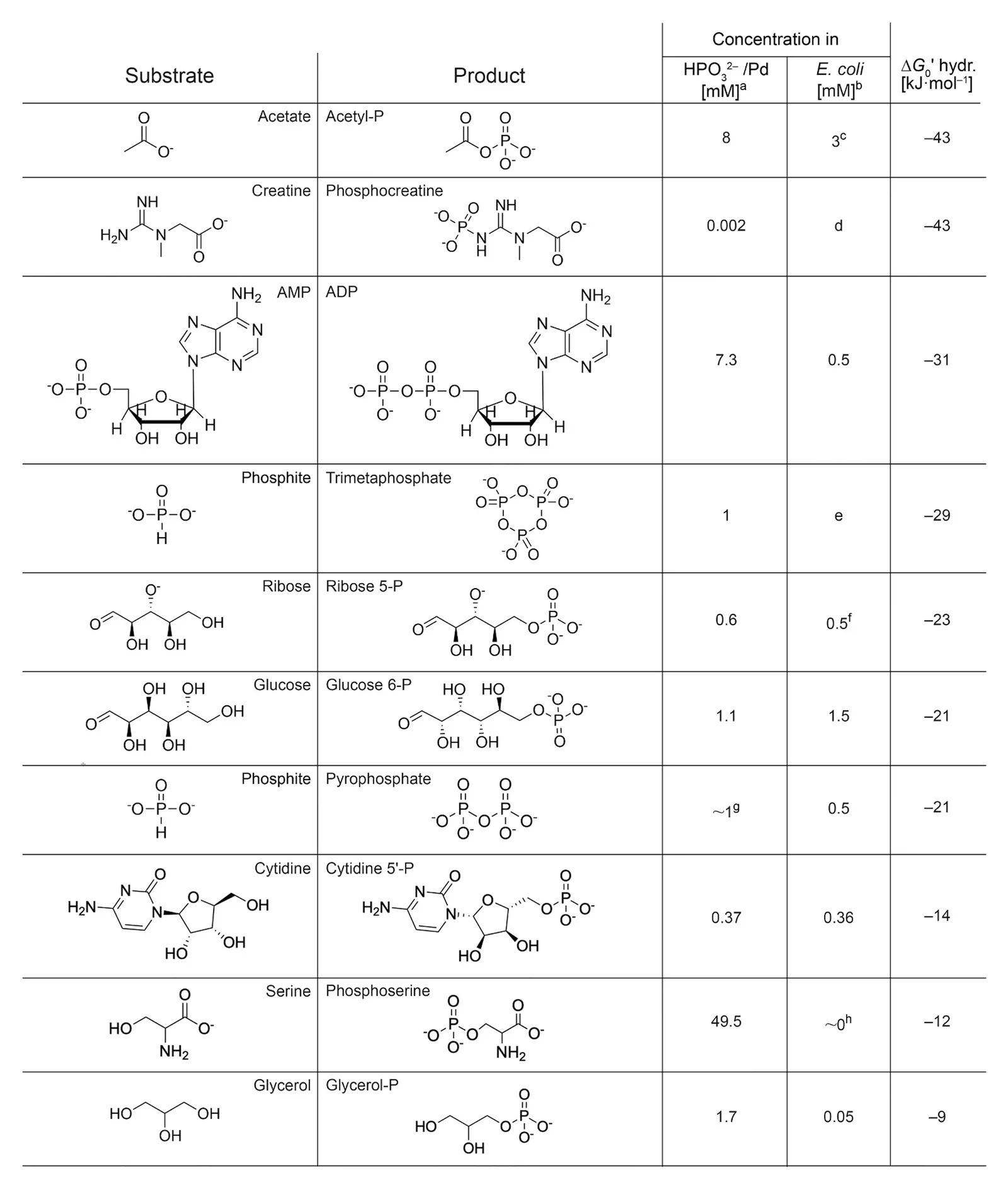
Products obtained by Schlikker et al. (2026) [26] in reactions of each substrate (100 mM) with 200 mM phosphite in water over Pd^0^ catalysts. Times 18–72 h, temperatures 25–80 ^°^C (details in Schlikker et al. (2026) [26]). ^a^) Concentrations of the phosphorylated product obtained with ${\text{HPO}}_{3}^{2-}$over Pd^0. b^) Cytosolic concentration of the product in *E. coli*, values from Bennett et al. (2009) [48] unless otherwise indicated. ^e^) From ref. Klein et al. (2007) [85]. ^d^) Does not occur in *E. coli*, a typical energy storage compound (phosphagen) of animal cells [86]; concentration determined by LC-MS [48]. ^e^) Meyerhof et al. (1953) [87] measured the free energy of hydrolysis of trimetaphosphate to three orthophosphate molecules as −88 kJ·mol^−1^ (−29.3 kJ·mol^−1^ per metaphosphate bond). ^f^) Estimated, Bennett et al. (2007) [48] report 1.3 mM for ribose-5-P, ribulose-5-P and xylulose-5-P combined. ^g^) Small specific peak in ^31^P NMR above detection limit of 1% conversion [26], the *E. coli* pyrophosphate concentration is from ref. [88]. ^h^) *E. coli* synthesizes cysteine via *O*-acetyl serine and expresses phosphoserine phosphatase that keeps phosphoserine levels low.

The formation of the phosphoanhydride bond in ADP in that reaction is energetically driven by the highly exergonic phosphite oxidation reaction:

$${{\text{HPO}}_{3}}^{2-}+{\text{H}}_{2}\text{O}\to {{\text{HPO}}_{4}}^{2-}+{\text{H}}_{2}$$

with Δ*G*_0_′ = −46 kJ·mol^−1^ [81]. Phosphite over Pd^0^ phosphorylates AMP, but it also phosphorylates serine, ribose, glucose, cytidine, glycerol, creatine and acetate [26] as shown in Fig. 2.

### 3.6 Physiologically relevant amounts

Phosphite has long been discussed as a theoretical source of phosphate at origins [41,82,83], but the discussion became more concrete with the isolation of bacteria that use phosphite as their sole source of phosphorus and electrons [69], which prompted the suggestion by Buckel (2001) [84] that phosphite oxidation could readily give rise to acetyl phosphate, an ancient currency of biochemical energy [16,21]. Subsequent studies reported evidence for phosphite in Hadean oceans [67] and detected phosphite in modern serpentinizing systems [66]. The discussion of phosphite as a source of phosphate and phosphorylation at origins became even more concrete with the recent demonstration that Pd^0^ catalyzes phosphite-dependent phosphorylation [26].

Though Pd^0^ phosphorylates a broad spectrum of substrates in water at mild temperatures, the yields in most cases were low, in the mM range (Fig. 2). From a ‘metabolism first’ perspective, the question is not whether the yields are close to 100%, rather the question is whether the yields are physiologically relevant. In Fig. 2, we have indicated the concentration of the corresponding product measured from the cytosol of growing *E. coli* cells [48] next to the concentration of phosphorylated product obtained with ${\text{HPO}}_{3}^{2-}$ in water. We are not suggesting that metabolite concentrations in *E. coli* are a relic of primordial phosphorylation reactions in the presence of 200 mM phosphite. Rather, we simply underscore the well-known observation that concentrations of metabolites involved in core carbon and energy pathways in growing cells of *E. coli* and other microbes are typically in the low mM to low μM range [48] and that a single native metal catalyst (Pd^0^) that naturally occurs in serpentinizing hydrothermal systems [71] generates physiologically relevant concentrations of phosphorylated products using ${\text{HPO}}_{3}^{2-}$ and Pd^0^ which both occur in serpentinized systems [66,71]. We emphasize that the reactions shown in Fig. 2 used 200 mM phosphite as a reactant although 75 mM ${\text{HPO}}_{3}^{2-}$ also works in some cases [26]. Aqueous phosphite concentrations within modern serpentinizing vents have so far not been measured.

We used 75–200 mM phosphite in order to have short reaction times. Is 75–200 mM outside the realm of the possible in a serpentinizing system? Two observations deserve consideration. First, serpentinizing systems expand rock volume by up to 44%, generating microfractured environments at the advancing waterfront and locally low water activities, hence high solute concentrations (phosphite liberation from the solid phase), on the micrometer scale [15] within the serpentinizing rock during the serpentinization process. Second, Herschy et al. [89] detected phosphite in several rock samples ca. 3.8 Ga in age. They showed that phosphate reduction with Fe(II) to 4% phosphite is facile at low hydrothermal temperatures of 160 ^°^C, and they estimated that Archaean oceans contained on the order of 0.1 mM ${\text{HPO}}_{3}^{2-}$ based on modern hydrothermal flux rates [89]. If global Archaean oceans contained 0.1 mM ${\text{HPO}}_{3}^{2-}$, the geological sources of that phosphite, including hydrothermal systems where phosphite has been detected [66], necessarily contained vastly more ${\text{HPO}}_{3}^{2-}$ that was continuously synthesized prior to the hydrothermal effluent being diluted in the open ocean. How much more ${\text{HPO}}_{3}^{2-}$ was present in Archaean vents is unknown, but a comparison from modern systems is useful: Actively serpentinizing hydrothermal systems can contain 5 mM H_2_ [18] which is roughly one million times more than the H_2_ concentration in the open ocean [90]. Phosphite has so far not been reported in the effluent of modern serpentinizing systems, though phosphite-oxidizing genes are enriched in that environment [64,65], indicating that aqueous phosphite is geochemically provided and (continuously) present somewhere within the system.

In contrast to traditional prebiotic phosphorylation methods, ${\text{HPO}}_{3}^{2-}$/Pd^0^ reactions operate in alkaline water, without nitriles or other condensing agents, in hours or days, at temperatures of 20–80 ^°^C and without recourse to drying cycles. The latter point is important, because one well-established school of thought on origins [91,92] infers the existence of evaporation-based wet-dry cycles on the early Earth to promote phosphorylation for RNA synthesis. Whatever prebiotic, environmental phosphorylation mechanism operated at the onset of metabolism and genetics, it had to operate continuously [15] until enzymes arose that could usurp the function of the prebiotic phosphorylation mechanism. This continuity calls for a geochemical setting that is itself in a far from equilibrium state. Geochemically generated, far from equilibrium conditions are an explicit foundation of the serpentinizing hydrothermal vent theory [14–18]. Spontaneous geochemical reactions between Fe(II)-rich rocks and water during serpentinization [57–60] generate pH and redox gradients [14–18] in a far from equilibrium geochemical setting. This thermodynamic potential is why serpentinizing systems convert H_2_ and CO_2_ to abiotic formate and methane today, the latter reaction being the same exergonic reaction used by modern methanogens for growth [93].

The standard prebiotic chemistry alternative to our continuous disequilibrium, Pd^0^/${\text{HPO}}_{3}^{2-}$ phosphorylation proposal is that desiccation (drying) was required at every phosphorylation step [92,94]. Under desiccative phosphorylation, the ribosomal synthesis of active enzymes, which requires 4 ATP per peptide bond [20], could not have taken place, because nascent polypeptides and ribosomes would have denatured at every desiccation-dependent step. Similar hurdles, but involving oxidative rather than desiccative damage to actively translating ribosomes, are encountered in proposals requiring stoichiometric equivalents of hydrogen peroxide in H_2_O_2_-dependent phosphorylation mechanisms [95]. The highly exergonic nature of the aqueous phosphite oxidation reaction [15,26] and the broad specificity of the Pd^0^ catalyst are desirable attributes for a prebiotic energy-conserving phosphorylation reaction [41,96], as are the natural co-occurrence of phosphorylating agent and activating catalyst in the same aqueous serpentinizing environment [15,26].

### 3.7 Do other PGEs also catalyze phosphorylation with $HPO_{3}^{2-}$?

**Fig. 3:**
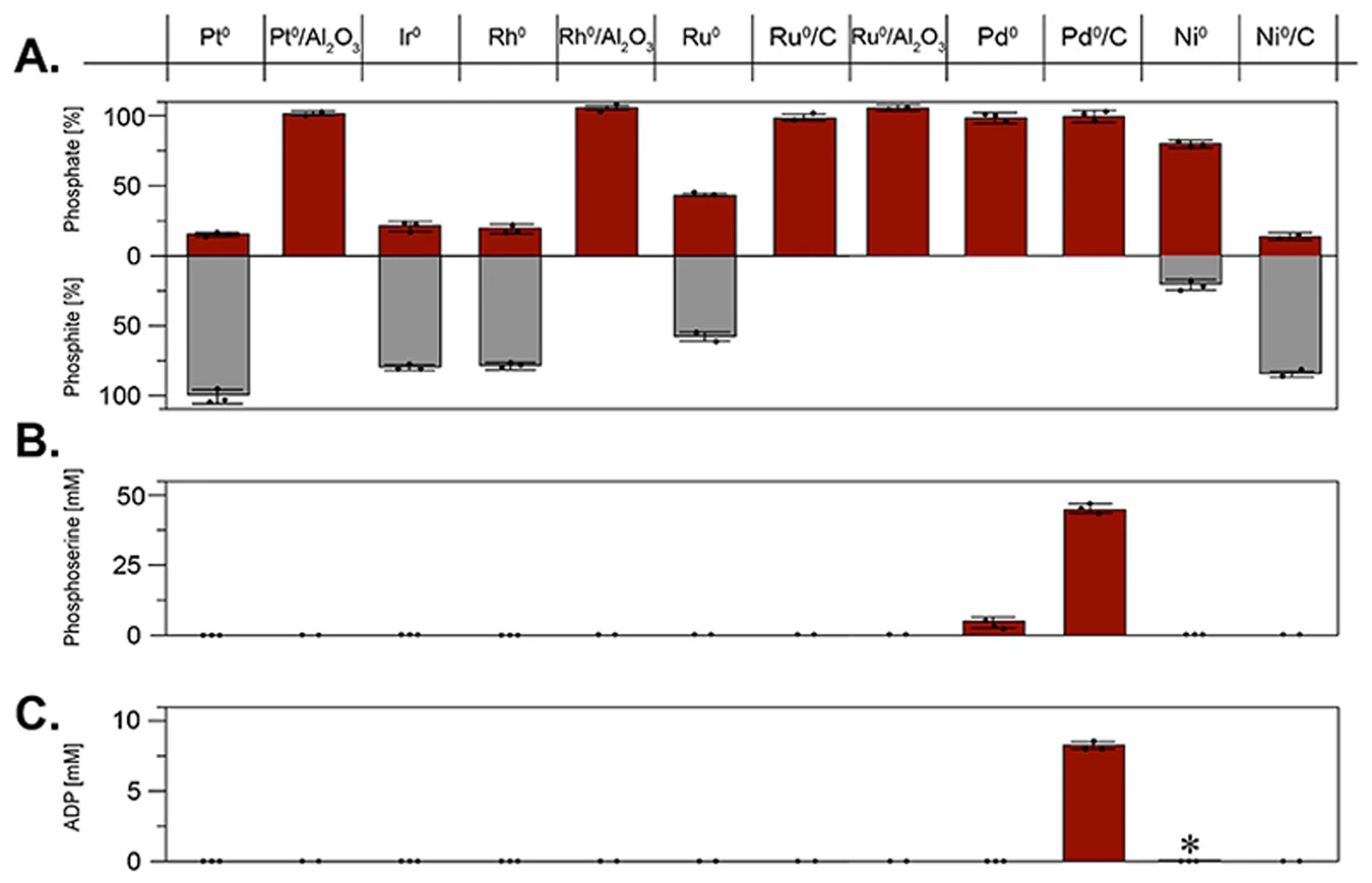
Comparison of platinum-group-element (PGE) and nickel-based catalysts for phosphite oxidation and phosphorylation reactions, control without catalyst at left. **A**, Phosphite oxidation to phosphate in the presence of different metal catalysts after incubation at 100 ^°^C for 72 h under 5 bar Ar in 1.5 mL aqueous solutions containing 200 mM sodium phosphite (^31^P NMR raw spectra shown in Fig. S1). **B**, Formation of phosphoserine from serine and phosphite after incubation at 50 ^°^C for 18 h (^31^P NMR raw spectra shown in Fig. S2). **C**, Formation of ADP from AMP and phosphite after incubation at 50 ^°^C for 18 h (^31^P NMR raw spectra shown in Fig. S3). All reactions contained 200 mM sodium phosphite. Supported catalysts were applied at loadings corresponding to approximately 0.15 mmol elemental metal, except for Pd/C, which was applied at 1.5 mmol catalyst (corresponding to approximately 0.15 mmol Pd^0^). The asterisk (*) indicates the formation of approximately 7 μM ADP, previously detected by LC–MS analysis in Schlikker et al. (2026) [26], whereas all other product concentrations were quantified by NMR spectroscopy. Osmium was not investigated owing to laboratory safety considerations. Native iron (Fe^0^) and awaruite (Ni_3_Fe) do not catalyze phosphite oxidation under these conditions [26].

In organic chemistry, PGEs are renowned for their catalytic properties, whereby most applications of PGE are in homogeneous catalysis: one atom of the metal is coordinated individually as a charged cation within a soluble organic molecule, sometimes more than one metal atom per molecule [97], pincer complexes being one example catalyst class [98], metal-organic frameworks [99] being another. The metals used here as catalysts are nanoparticles, either on supports such as carbon or aluminum silicate, or as pure metal nanoparticles. A typical 2 nm diameter Pd nanoparticle will have a few hundred atoms, most commercial nanoparticles are larger. Importantly, the Pd particles we use are not dissolved, they are in the solid phase, they have a concentration of zero in solution and their structure is amorphous, so that the number of catalytically active atoms per particle, if any, is unknown. Pd and other PGE are deposited and enriched in serpentinizing hydrothermal systems, often as very small particles [71]. We investigated Pt, Ru, Rh and Ir in comparison to Pd for their ability to oxidize phosphite and catalyze two phosphorylation reactions. A comparative screen of PGE and nickel-based catalysts was performed. Though all PGE catalysts tested readily promoted phosphite oxidation under hydrothermal conditions, only Pd^0^ efficiently catalyzed the formation of phosphorylated products, including ADP and *O*-phosphoserine, as detected by ^31^P NMR spectroscopy (Fig. 3). Because ^31^P NMR has a relatively low sensitivity corresponding to approximately 1% conversion, lower levels of phosphorylation activity for PGE other than Pd^0^ cannot be excluded.

Neither Ru^0^/C nor Ni^0^/C generated detectable phosphorylated organics. Also, there is ten times less metal in the reaction when we use carbon-supported metals, except for Pd/C, where the catalyst loading was adjusted to provide the same amount of elemental Pd. The C support environment is apparently conducive to phosphorylation in the case of Pd^0^. Phosphite oxidation is highly exergonic (-46 kJ per mol of phosphate produced), hence the equilibrium should lie far on the side of phosphate. In the presence of several catalysts tested, the reaction goes to completion, but for several catalysts, over 50% of the phosphite did not react (Fig. 3A). This suggests either very slow reaction rates or the presence of reactions that poison the catalyst surface. In the absence of catalysts, phosphite does not undergo oxidation to phosphate in water under anaerobic conditions. The aluminum silicate supported metals are effective catalysts for phosphite oxidation to phosphate, but do not yield phosphorylated products detectable by ^31^P NMR (Fig. 3). In the case of Ni^0^, liquid chromatography and mass spectrometry (LCMS) detected 6.7 μM ADP product (0.0067% conversion) [15], indicated by an asterisk in Fig. 3C. The most common form of palladium in serpentinizing systems is palladium awaruite, Pd_n_Ni_3_Fe [71], which is also active as a solid state catalyst in phosphite oxidation and phosphorylation experiments [26].

Despite lack of direct evidence for the nature of reaction intermediates in our Pd-catalyzed phosphite oxidation and phosphorylation reactions, enzymatic studies and some observations suggest a possible reaction mechanism involving hydride removal, generating metaphosphate [PO_3_]^−^ as an intermediate and active phosphorylation species [26]. We have not observed any cases in which native metals generate phosphorylated products using phosphate as reactant [26], indicating that the phosphite oxidation reaction itself is required for phosphorylation. Enzymatic phosphite oxidation is NAD^+^ dependent in cases studied so far [62,100] indicating hydride removal in the reaction mechanism [68]. Nagaosa and Aoyama [101] reported the oxidation of phosphite to phosphate in aqueous solution using Pd^0^ and Pd/C in the presence of air (21% O_2_). The ability of Pd^0^ to catalyze O_2_-dependent phosphite oxidation is known, the anaerobic route of phosphite oxidation using Pd^0^ is new. Although neither Ni^0^ nor Pd^0^ can serve as oxidants, they are good hydride acceptors [102,103] and can readily catalyze the synthesis of H_2_ in water via chemisorption in the presence of a strong reductant, which phosphite is (*E*_0_′ = − 690 mV) [69]. Third, metaphosphate is implicated in some enzymatic reactions. Meta-phosphate [PO_3_]^−^ is a planar ion and highly unstable in water. Westheimer (1981) [104] reports the estimated free energy of the reaction

$${\left[{\text{PO}}_{3}\right]}^{-}+{\text{H}}_{2}\text{O}\to {\text{H}}_{2}{\text{PO}}_{4}^{-}$$

as −107 kJ·mol^−1^ and free metaphosphate is never observed in water [3]. However, metaphosphate is implicated or detected in some enzyme reactions. For example, the dissociative mechanism proposed for *Bacillus* sp. phosphite oxidase [100] involves a metaphosphate intermediate. Metaphosphate is discussed as an intermediate in enzymatic phosphoester hydrolysis [105]. Fructose-1,6-bisphosphatase has been reported to generate metaphosphate as an enzyme bound reaction intermediate identifiable in the crystal structure [106]. Quantum mechanical studies of phosphoserine phosphatase [107] and mechanistic studies of phosphoglucomutase [108] also suggest the participation of metaphosphate intermediates. The accumulation of trimetaphosphate (Fig. 2) and polyphosphates in our experiments [26] would be compatible with a metaphosphate intermediate.

### 3.8 Phosphorylation in the early evolutionary process

**Fig. 4:**
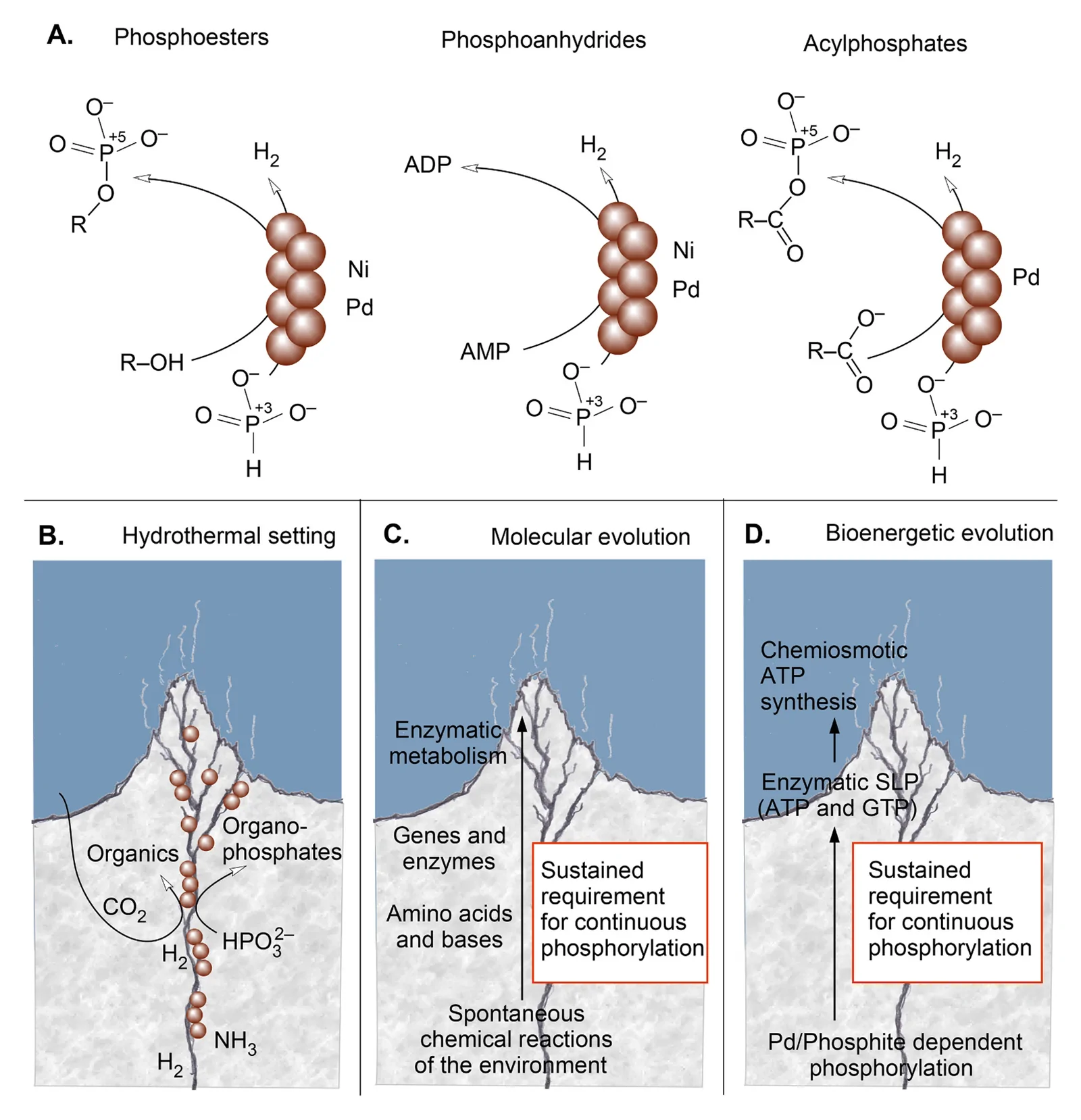
Phosphite-dependent phosphorylation over Pd^0^ in the palaeoecological context of molecular, metabolic and bioenergetic evolution in serpentinizing hydrothermal vents: a chemically reactive site of concomitant organic synthesis and phosphorylation, immune to disruption by thunderstorms, wind, drought, erosion, UV light, bolide impact, tsunami or any combination of the above, and independent of the low solubility of phosphate [117]. Archaean oceans contained phosphite [89], as do modern serpentinized rocks [66].

Aqueous phosphorylation under serpentinizing conditions impacts views of bioenergetic evolution [25,46]. Under conditions of serpentinizing hydrothermal vents [93], Pd^0^/${\text{HPO}}_{3}^{2-}$ generates the three main kinds of phosphate bonds that occur in core biosynthetic metabolism [15,31]: phosphoesters, phosphoanhydrides and acylphosphates (Fig. 4A). The organic substrates for those phosphorylation reactions can be synthesized from H_2_ and CO_2_ by native metal catalysts under the same serpentinizing conditions [26,36,37,52,77] (Fig. 4B). A single, aqueous site for the carbon skeleton synthesis and product phosphorylation in the hydrothermal vent model avoids patchwork synthesis and product loss inherent to models that involve transport of intermediates from one reaction locality to another [91]. The concentrating properties of inorganic microcompartments at vents in the presence of temperature gradients (thermophoresis) are well-known [109,110].

Whatever environmental mechanism of phosphorylation operated before the origin of genes and enzymes, it had to operate continuously [15] at the required site of synthesis until enzymes arose that could usurp the function of the environmental phosphorylation process (Fig. 4C). This puts severe constraints on the environment and nature of prebiotic phosphorylation, because it stipulates that the environmental phosphorylation mechanism had to be compatible with and coexist with, early generations of proteins (enzymes) until these could themselves provide phosphorylation, and it had to operate to support genetically encoded enzyme synthesis on ribosomes. No enzymes are known that harness oven-drying, phosphides, P_2_O_5_ or condensing agents such as cyanoformamide as a source of energy for phosphorylation [94,111–115]. Moreover, no enzymes would preserve their activity in such harsh conditions; they would irreversibly denature at the first phosphorylation reaction. By contrast, the AdpA enzyme, AMP-dependent phosphite dehydrogenase, *does* harness the energy contained in the phosphite oxidation reaction to perform a substrate-level phosphorylation [68] reaction in aqueous solution (bacterial cytosol). In the ADP-forming reaction in Fig. 2, Pd^0^ merely substitutes for the AdpA enzyme and its NAD^+^ cofactor [68]. Until enzymes invented means to harness environmental energy in such a way as to *replace abiotic phosphorylation*, there existed a non-negotiable demand on the environment to provide a sustained supply of continuous phosphorylation that was compatible with enzymatic chemistry, as indicated in Fig. 4C. In contrast to harsh chemistry or oven-drying for phosphorylation, aqueous phosphite oxidation affords continuity from prebiotic to enzymatic reactions.

What is the ultimate source of the energy required to synthesize phosphite for early chemical evolution? In the simplest alternatives, either (i) a portion of the phosphorus on the Earth’s early crust following the Moon forming impact was present as phosphite, or (ii) perhaps more likely, phosphite was produced from phosphate minerals in situ in serpentinizing hydrothermal vents [66,89], which have been in existence for as long as liquid water [58] and which generate the extremely reducing conditions needed to convert phosphate to phosphite (*E*_0_ = −690 mV). In the latter case, the source of energy was the same as today: rock-water interactions [116], whereby the amount of phosphite and Pd^0^ required was governed by the local environment of specific hydro-thermal vents (Fig. 4), not global ocean or atmospheric conditions.

### 3.9 A starting point for the bioenergetic process

There are only two sources of net phosphorylation known in life: substrate-level phosphorylation and chemiosmotic gradient harnessing via ATP synthases [7,118]. Substrate-level phosphorylation (SLP) involves stoichiometric reactions of organophosphates with sufficient group transfer potential to generate ATP (or ADP from AMP, or GTP from GDP). Such organophosphates are typically acyl phosphates such as 1,3-bisphosphoglycerate, succinyl phosphate or acetyl phosphate and the like [21,22]. Acetyl phosphate has been discussed as a candidate for such a primordial bioenergetic driver [16]. Abiotic synthesis of acetyl phosphate has been previously reported involving, however, reactants unlikely to have formed by abiotic means and with no evident role for catalysts [55,119,120]. Schreibersite has also been proposed as a source of prebiotic phosphorylation, but its only known natural occurrence is in meteorites, and because it generates radicals, the reported reaction products [121] included non-physiological compounds arising from one-electron radical chemistry that attacked the methyl rather than the carboxyl group of acetate.

The synthesis of acetyl phosphate from phosphite and acetate [26] identifies an inorganically catalyzed SLP reaction involving substrates and catalysts that occur under serpentinizing conditions. At present, the other mechanism of net ATP supply, chemiosmotic gradient harnessing via ATP synthases [7,118] has no known inorganically catalyzed analogue. Although modern ATP synthases can be driven by synthetically generated ion gradients across chemically primitive membranes consisting only of fatty acids (isoprene alcohols do not sustain a gradient) [122], no structurally or functionally primitive ATP synthases are known, calling for mechanisms of biochemical phosphorylation that predate chemiosmotic harnessing in evolution [123]. Phosphorylation by ${\text{HPO}}_{3}^{2-}$ over Pd^0^ is such a mechanism.

A critic might ask: if Pd^0^ is such an efficient catalyst, why was it never incorporated into metabolism? This issue was recently addressed for native nickel [80]. Nickel catalyzes a broad spectrum of reactions in vitro as Ni^0^ nanoparticles [80], while in vivo nickel is active in enzymes and cofactors only as Ni^2+^ ions coordinated by N, S, O and C atoms [80]. Cells can, in principle, convert Ni^2+^ to Ni^0^ (*E*_0_′ = −260 mV) using low potential ferredoxins (*E*_0_′ = −450 mV). But the substrate specificity of Ni^0^ encompasses the activity of several divergent and unrelated enzyme classes [80]: cells would be unable to keep cytosolic Ni^0^ from reacting indiscriminately with H_2_, NADH, carbonyl, vinyl, amino or other moieties [77]. An indiscriminately active Ni^0^ catalyst in the cytosol would disrupt the network of high-specificity enzymatic reactions that comprise metabolism [80]. The same disruptive argument applies to Pd^0^, with the additional limitation that Pd^0^ is more noble than Ag^0^, resisting oxidation even by O_2_, precluding primordial Pd^2+^ generation for import into the cytosol. The virtue of native metals at origins—broad catalytic activity to promote many different reactions—is their drawback in enzymatic metabolism [52]. When metalloenzymes and metal-locofactors arose in evolution [14], metabolism and ribosomes could synthesize specific catalysts that use soluble, importable, coordination-amenable and controllable metal ions [52,80], which replaced native metals, thereby liberating metabolism from solid state catalysts in the crust [15,16].

As with metabolic evolution (Fig. 4C), whatever environmental mechanism of phosphorylation operated before the origin of enzymatic SLP, it had to operate continuously at the required site of synthesis until enzymes arose that could usurp the function of the environmental phosphorylation process (Fig. 4D). And even then, the first enzymes of SLP required an environmentally supplied organophosphate or inorganic phosphodonor capable of phosphorylating ADP. Phosphite over Pd^0^ could be that supply. Phosphite oxidation over Pd^0^ presents a broad-specificity, physiologically useful, geologically sustainable and environmentally supplied source of phosphorylation at origins. It can also generate acyl phosphates and, once they arise in metabolism, nucleoside phosphates such as ADP, which serve as additional phosphodonors in non-enzymatic phosphorylation reactions [124]. This would generate short, exergonic phosphotransfer cascades at origins, similar to the short phosphotransfer cascades in prokaryotic cells [125]. For those who have argued for decades in favor of the evolutionary significance of hydrothermal vents in spawning life [19,126,127], phosphite over Pd^0^ presents progress on the origins problem, as it identifies a mechanism of metabolic phosphorylation using catalysts and substrates supplied by serpentinizing hydrothermal vents, where anaerobic autotrophs still thrive today [60,93].

## Supplementary Material

**Appendix A. Supplementary data**

Supplementary data to this article can be found online at https://doi.org/10.1016/j.bbabio.2026.149610.

Supplementary material

## Data Availability

Data will be made available on request.
