# Supplementary material for: Metal-catalyzed phosphorylation by phosphite and energy at origins
Source: Biochim Biophys Acta Bioenerg. Author manuscript; Available in PMC 2026 Nov 5. (PMC7619703; doi:10.1016/j.bbabio.2026.149610)
Supplement: Supplementary file 1 [file EMS220409-supplement-Supplementary_material.pdf]

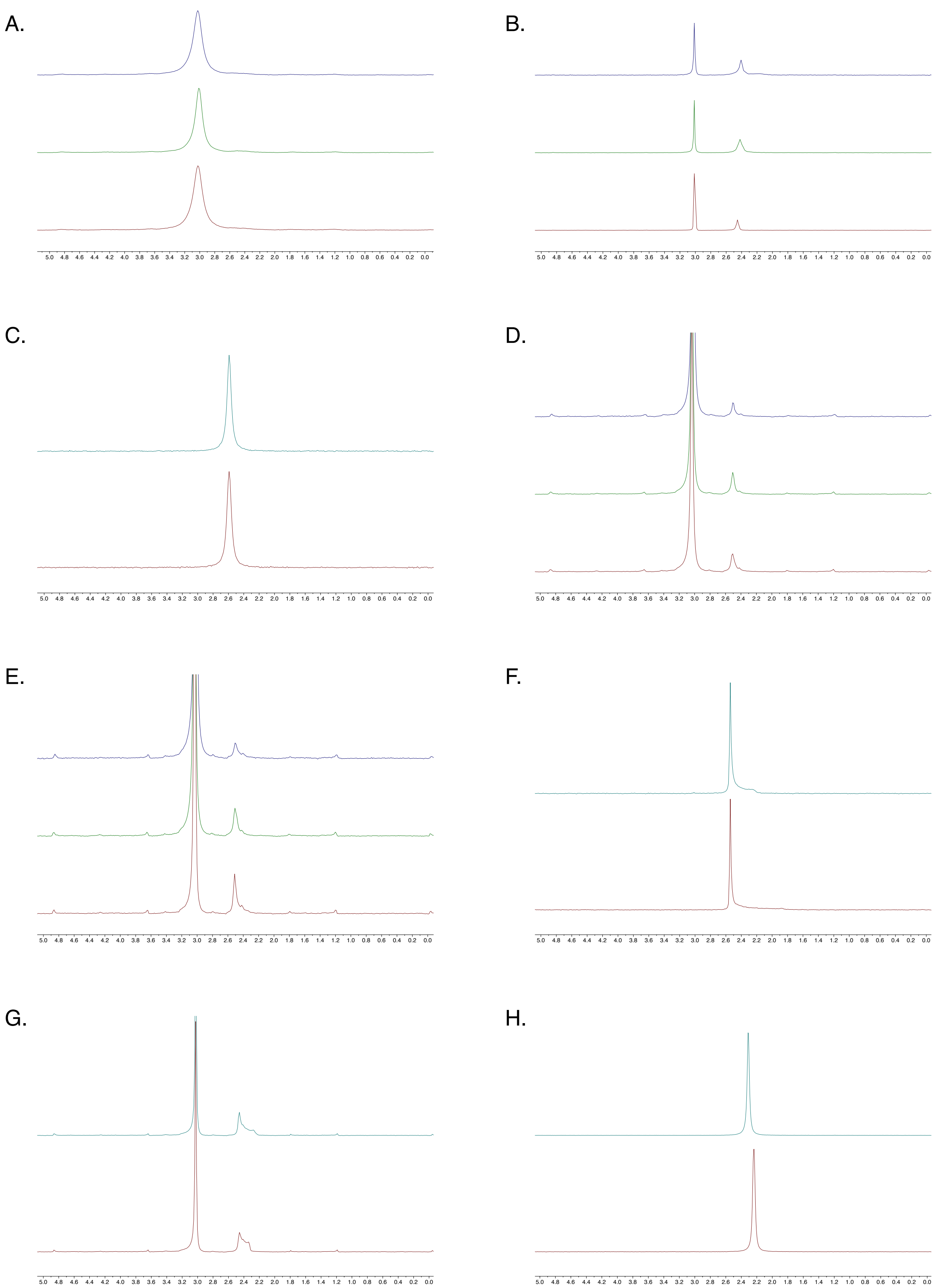
A.
B.
C.
D.
E.
F.
G.
H.

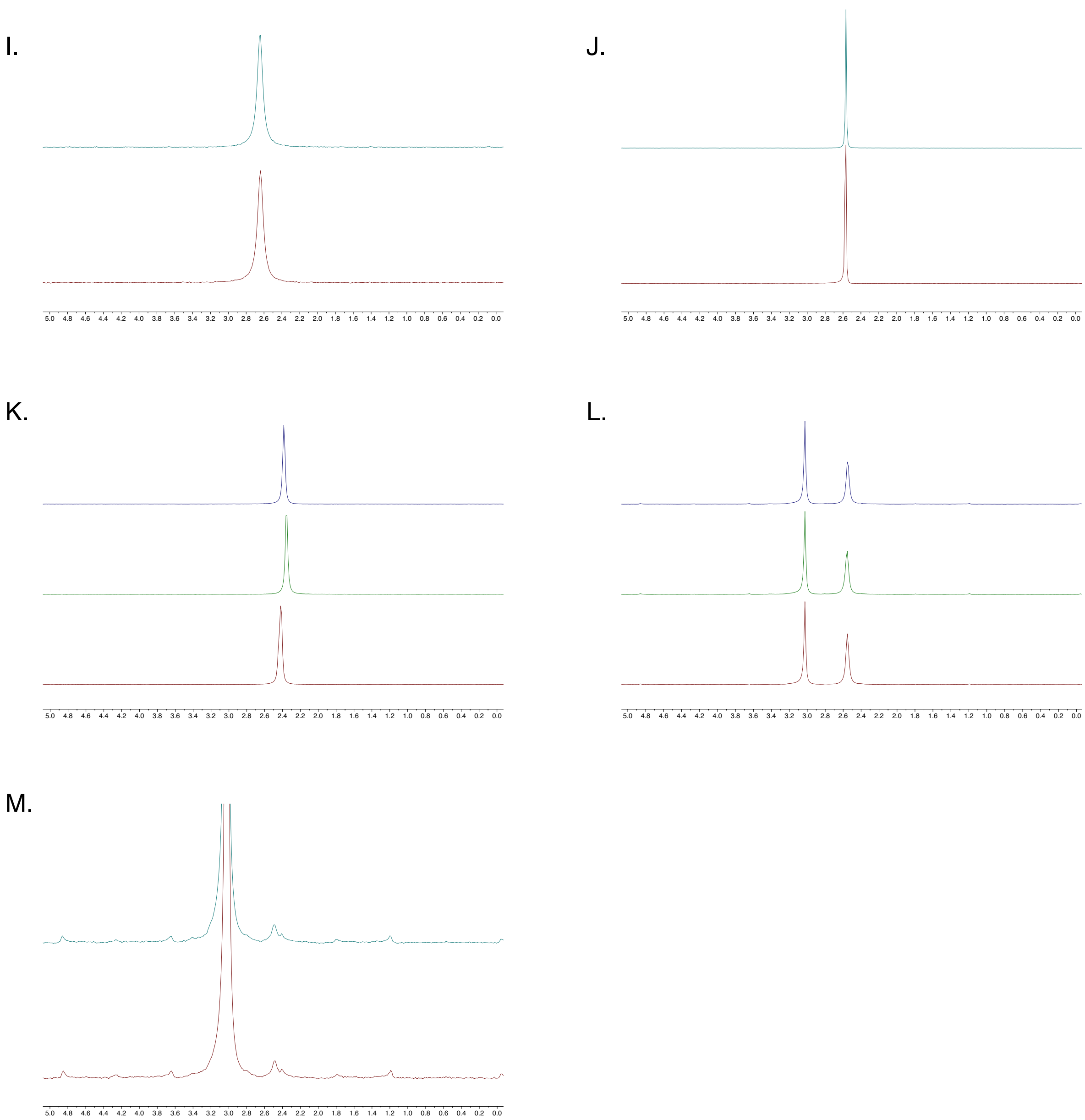

Figure S1. ^{31}P-NMR spectra corresponding to the phosphite oxidation experiments shown in Figure 3 A after incubation at 100 °C for 72 h with different metal catalysts. All reactions contained 200 mM phosphite and 0.15 mmol catalyst, except for Pd/C, where 1.5 mmol catalyst was used. Spectra are shown for (A) control without catalyst, (B) Pt^0, (C) Pt^0/Al_2O_3, (D) Ir^0, (E) Rh^0, (F) Rh^0/Al_2O_3, (G) Ru^0, (H) Ru^0/C, (I) Ru^0/Al_2O_3, (J) Pd^0, (K) Pd^0/C, (L) Ni^0, and (M) Ni^0/C.

A.

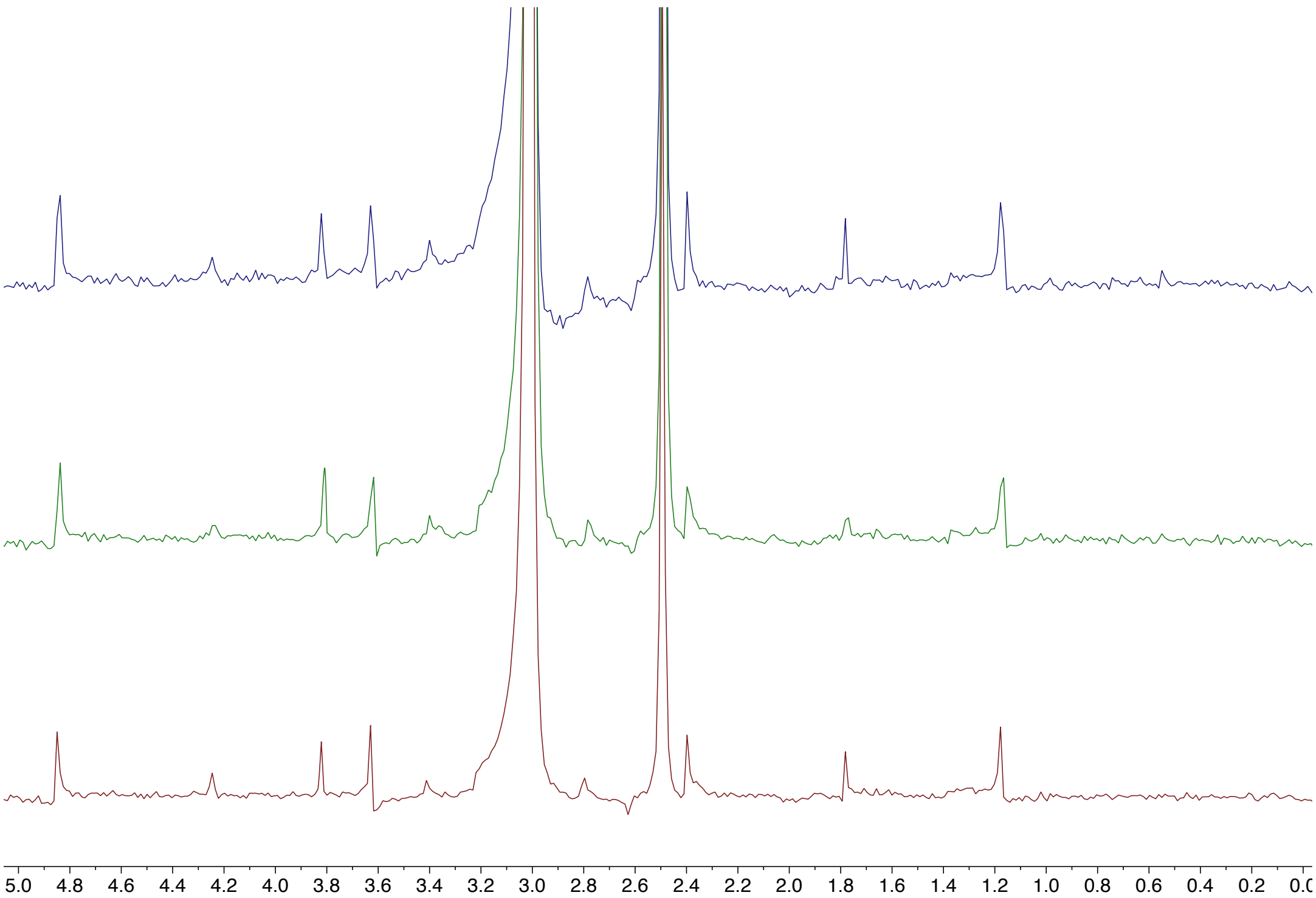

B.

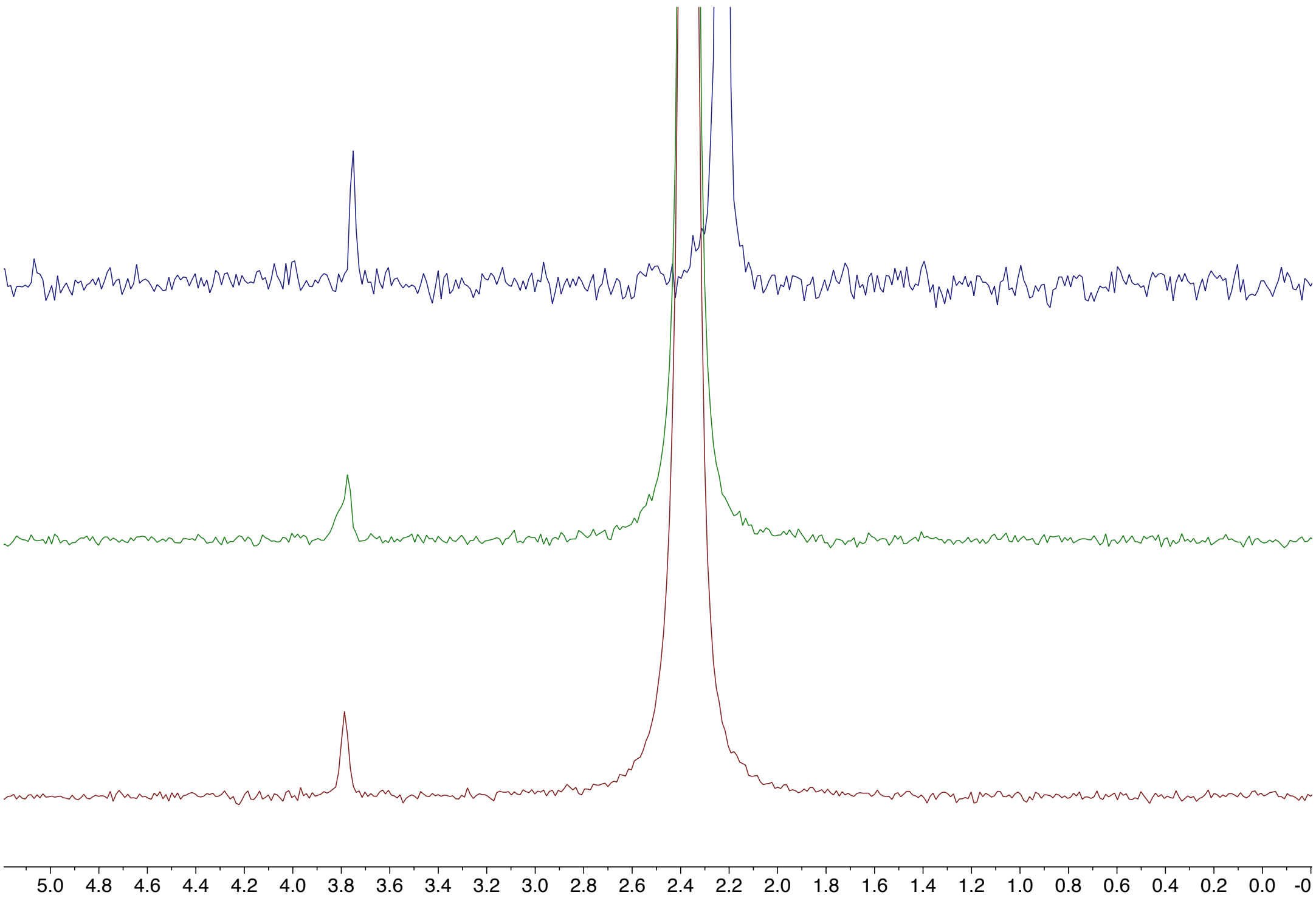

Figure S2. Raw ^{31}P-NMR spectra corresponding to the phosphoserine formation experiments shown in Figure 3 B after incubation at 50 °C for 18 h. All reactions contained 100 mM serine and 200 mM phosphite. (A) shows the reaction with Pd^0, and (B) the reaction with Pd/C.

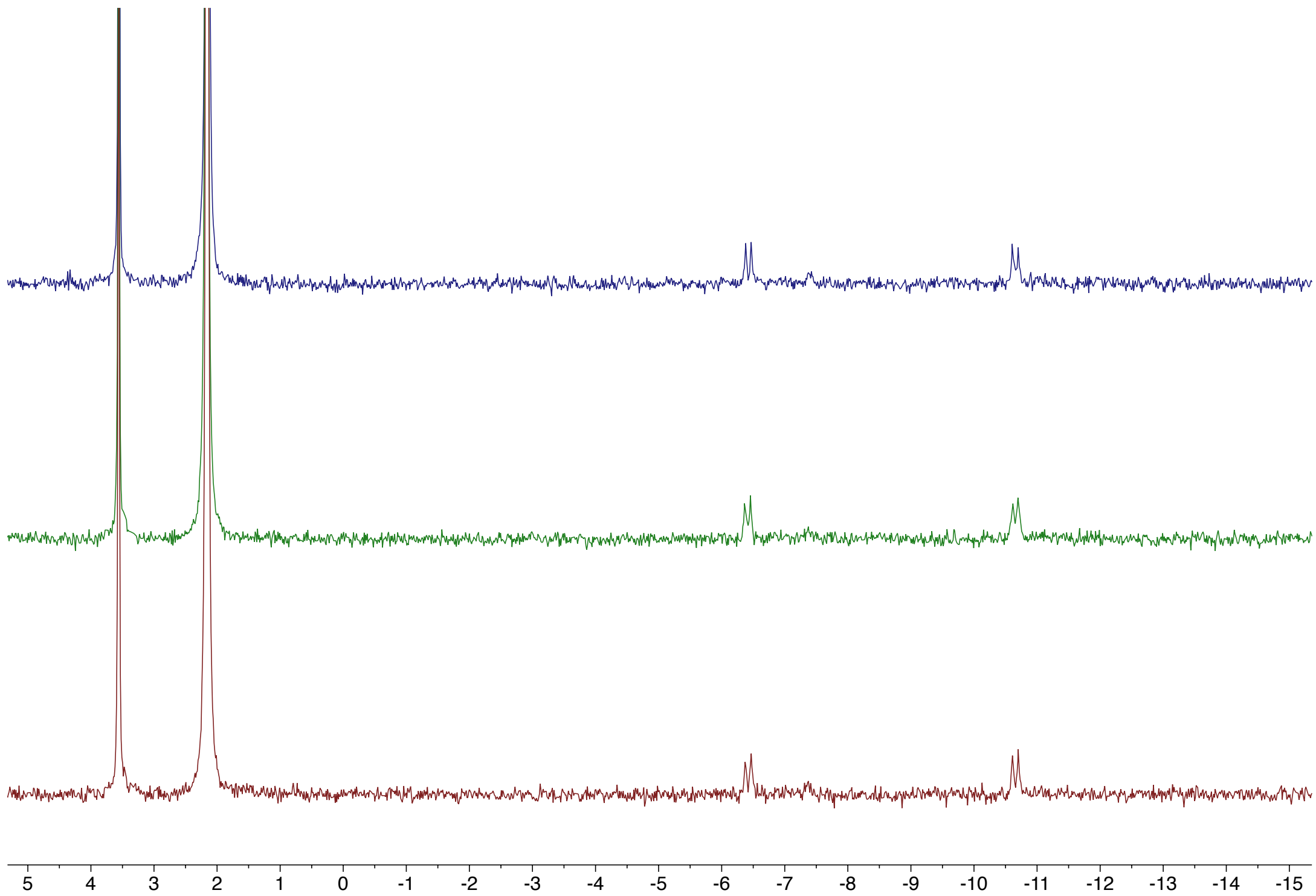

Figure S3. Raw ^{31}P-NMR spectrum corresponding to the AMP phosphorylation experiment shown in Figure 3 C after incubation at 50 °C for 18 h. The reaction contained 100 mM AMP and 200 mM phosphite.

Table S1. Overview of all reactions included in Figure 2, including Reaction ID, stratification rank, recoded reaction equation, reaction equations in compound names (left and right), pathway information.

ReactionID	Rank	Recodedreactionequation	Reactionequation(Compoundname)left	Reactionequation(Compoundname)right
R00134	1	C00011 + C00005 + C00080 <=>C00058 + C00006	CO2 + NADPH+ H+	Formate + NADP+
R01195	1	2C00138+C00006+C00080<=>2C00139+C00005	2Reducedferredoxin+NADP++H+	2Oxidizedferredoxin+NADPH
R03015	1	C00862 + 2C00138 + C00011 +2 C00080 <=>C01001 + 2C00139 + C00001	Methanofuran+ 2Reducedferredoxin+ CO2+ 2H+	Formylmethanofuran +2 Oxidized ferredoxin +H2O
R07157	1	C00011 + 2 C00138+ 2 C00080<=> C00237+ C00001 +2 C00139	CO2 + 2Reduced ferredoxin +2 H+	CO+ H2O+ 2 Oxidizedferredoxin
R10092	1	C00011 + C00001 <=>C00288 + C00080	CO2 + H2O	HCO3-+ H+
R00149	1	2C00002+C00014+C00011+C00001<=>2C00008+C00009+C00169	2ATP+Ammonia+CO2+H2O	2ADP+Orthophosphate+Carbamoylphosphate
RMAN4	1	C00011 + C00282 <=>C00058 + C00080	CO2 + Hydrogen	Formate +H+
R00019	2	C00282 + 2C00139 <=>2 C00138 +2 C00080	Hydrogen + 2Oxidized ferredoxin	2Reducedferredoxin+2H+
R03390	2	C01001 + C01217 <=>C00862 + C01274	Formylmethanofuran +5,6,7,8-Tetrahydromethanopterin	Methanofuran + 5-Formyl-5,6,7,8-tetrahydromethanopterin
R00943	2	C00101 + C00058 + C00002 <=>C00008 + C00009 + C00234	Tetrahydrofolate +Formate + ATP	ADP +Orthophosphate + 10-Formyltetrahydrofolate
R03464	3	C01274 + C00080 <=>C04330 + C00001	5-Formyl-5,6,7,8-tetrahydromethanopterin + H+	5,10-Methenyltetrahydromethanopterin + H2O
R01655	3	C00234 + C00080 <=>C00445 + C00001	10-Formyltetrahydrofolate + H+	5,10-Methenyltetrahydrofolate + H2O
R04455	4	C04330 + C00282 <=>C04377 + C00080	5,10-Methenyltetrahydromethanopterin +Hydrogen	5,10-Methylenetetrahydromethanopterin + H+
R01220	4	C00445 + C00005 <=>C00143 + C00006	5,10-Methenyltetrahydrofolate + NADPH	5,10-Methylenetetrahydrofolate + NADP+
R04456	4	C04330 + C01080<=> C04377+ C00876 +C00080	5,10-Methenyltetrahydromethanopterin+ Reduced coenzyme F420	5,10-Methylenetetrahydromethanopterin +Coenzyme F420 + H+
R07168	5	C00143 + C00004 + C00080 <=>C00440 + C00003	5,10-Methylenetetrahydrofolate + NADH +H+	5-Methyltetrahydrofolate + NAD+
R04464	5	C04377 + C01080 <=>C04488 + C00876	5,10-Methylenetetrahydromethanopterin+ Reduced coenzyme F420	5-Methyl-5,6,7,8-tetrahydromethanopterin +Coenzyme F420
R03025	5	C00876 + C00282 <=>C01080	Coenzyme F420 +Hydrogen	Reduced coenzyme F420
R10243	6	C00440 + C00010+ C00237 <=>C00101 + C00024	5-Methyltetrahydrofolate +CoA + CO	Tetrahydrofolate + Acetyl-CoA
R09096	6	C00010 + C04488+ C00237 <=> C00024 + C01217	CoA + 5-Methyl-5,6,7,8-tetrahydromethanopterin+ CO	Acetyl-CoA + 5,6,7,8-Tetrahydromethanopterin
R01196	7	2C00138+C00024+C00011+2C00080<=>2C00139+C00022+C00010	2Reducedferredoxin+Acetyl-CoA+CO2+2H+	2Oxidizedferredoxin+Pyruvate+CoA
R00230	7	C00024 + C00009 <=>C00010 + C00227	Acetyl-CoA + Orthophosphate	CoA + Acetyl phosphate
R00742	7	C00002 + C00024+ C00288 <=>C00008 + C00009+ C00083	ATP + Acetyl-CoA+ HCO3-	ADP +Orthophosphate + Malonyl-CoA
R10866	7	C00024 + C00011 +C02745 <=>C00022 + C00010+ C02869	Acetyl-CoA + CO2 +Reduced flavodoxin	Pyruvate + CoA +Oxidized flavodoxin
R00396	8	C00022 + C00014+ C00004 +C00080 <=>C00041 + C00003+ C00001	Pyruvate + Ammonia +NADH+ H+	L-Alanine +NAD+ + H2O
R00226	8	2C00022<=>C06010+C00011	2Pyruvate	(S)-2-Acetolactate +CO2
R01626	8	C00083 + C00229 <=>C00010 + C01209	Malonyl-CoA + Acyl-carrierprotein	CoA +Malonyl-[acyl-carrierprotein]
R07399	8	C00024 + C00022+ C00001 <=>C02612 + C00010	Acetyl-CoA +Pyruvate + H2O	(R)-2-Methylmalate + CoA
R00206	8	C00002 + C00022+ C00009 <=>C00020 + C00074+ C00013	ATP + Pyruvate +Orthophosphate	AMP +Phosphoenolpyruvate + Diphosphate
R00199	8	C00002 + C00022+ C00001 <=>C00020 + C00074+ C00009	ATP + Pyruvate+ H2O	AMP +Phosphoenolpyruvate + Orthophosphate
R00344	8	C00002 + C00022+ C00288 <=>C00008 + C00009+ C00036	ATP +Pyruvate + HCO3-	ADP +Orthophosphate + Oxaloacetate
R00342	9	C00036 + C00004+ C00080 <=>C00149 + C00003	Oxaloacetate + NADH+ H+	(S)-Malate + NAD+
R05071	9	C06010 <=> C04181	(S)-2-Acetolactate	3-Hydroxy-3-methyl-2-oxobutanoicacid
R04439	9	C06010 + C00005+ C00080 <=>C04272 + C00006	(S)-2-Acetolactate + NADPH+ H+	(R)-2,3-Dihydroxy-3-methylbutanoate + NADP+
R00345	9	C00001 + C00074+ C00011 <=>C00009 + C00036	H2O+ Phosphoenolpyruvate+ CO2	Orthophosphate +Oxaloacetate
R00431	9	C00044 + C00036<=> C00035+ C00074 +C00011	GTP + Oxaloacetate	GDP + Phosphoenolpyruvate+ CO2
R00341	9	C00002 + C00036<=> C00008+ C00074 +C00011	ATP + Oxaloacetate	ADP +Phosphoenolpyruvate + CO2
R00658	9	C00074 + C00001 <=>C00631	Phosphoenolpyruvate + H2O	2-Phospho-D-glycerate
R03896	9	C02612 <=>C02226 + C00001	(R)-2-Methylmalate	2-Methylmaleate + H2O
R07476	9	C00094 + C00074<=> C11536	Sulfite + Phosphoenolpyruvate	(2R)-O-Phospho-3-sulfolactate
R04440	10	C04181 + C00005+ C00080 <=>C04272 + C00006	3-Hydroxy-3-methyl-2-oxobutanoicacid + NADPH+ H+	(R)-2,3-Dihydroxy-3-methylbutanoate + NADP+
R10115	10	C19673 + C01209<=> C20372+ C00011 + C00229	Malonyl-[acp] methyl ester +Malonyl-[acyl-carrier protein]	3-Ketoglutaryl-[acp] methyl ester+ CO2 + Acyl-carrierprotein
R05789	10	C11536 + C00001<=> C11537 +C00009	(2R)-O-Phospho-3-sulfolactate + H2O	(2R)-3-Sulfolactate +Orthophosphate
R01082	10	C00149 <=>C00122 + C00001	(S)-Malate	Fumarate + H2O
R03898	10	C02226 + C00001 <=>C06032	2-Methylmaleate + H2O	D-erythro-3-Methylmalate
R04441	10	C04272 <=>C00141 + C00001	(R)-2,3-Dihydroxy-3-methylbutanoate	3-Methyl-2-oxobutanoic acid + H2O
R01518	10	C00631 <=> C00197	2-Phospho-D-glycerate	3-Phospho-D-glycerate
R10116	11	C20372 + C00005+ C00080 <=>C20373 + C00006	3-Ketoglutaryl-[acp] methyl ester + NADPH+ H+	3-Hydroxyglutaryl-[acp] methyl ester+ NADP+
R07136	11	C11537 + C00003 <=>C05528 + C00004+ C00080	(2R)-3-Sulfolactate + NAD+	3-Sulfopyruvate + NADH +H+
R00994	11	C06032 + C00003<=> C00109+ C00011 + C00004+ C00080	D-erythro-3-Methylmalate + NAD+	2-Oxobutanoate +CO2 + NADH+ H+
R01513	11	C00197 + C00003<=> C03232+ C00004 +C00080	3-Phospho-D-glycerate + NAD+	3-Phosphonooxypyruvate + NADH+ H+
R00402	11	C00122 + C00004+ C00080 <=>C00042 + C00003	Fumarate + NADH+ H+	Succinate + NAD+
R01226	11	C00143 + C00141+ C00001 <=>C00101 + C00966	5,10-Methylenetetrahydrofolate +3-Methyl-2-oxobutanoic acid + H2O	Tetrahydrofolate + 2-Dehydropantoate
R01213	11	C00024 + C00141+ C00001 <=>C02504 + C00010	Acetyl-CoA + 3-Methyl-2-oxobutanoicacid+ H2O	alpha-Isopropylmalate + CoA
R01512	11	C00002 + C00197 <=>C00008 + C00236	ATP + 3-Phospho-D-glycerate	ADP +3-Phospho-D-glyceroyl phosphate
R02472	12	C00966 + C00005+ C00080 <=>C00522 + C00006	2-Dehydropantoate + NADPH +H+	(R)-Pantoate + NADP+
R01061	12	C00236 + C00004+ C00080 <=>C00118 + C00009 +C00003	3-Phospho-D-glyceroyl phosphate +NADH+ H+	D-Glyceraldehyde 3-phosphate +Orthophosphate +NAD+
R01063	12	C00236 + C00005+ C00080 <=>C00118 + C00009 +C00006	3-Phospho-D-glyceroyl phosphate +NADPH+ H+	D-Glyceraldehyde 3-phosphate +Orthophosphate + NADP+
R08648	12	C00022 + C00109<=> C06006+ C00011	Pyruvate + 2-Oxobutanoate	(S)-2-Aceto-2-hydroxybutanoate +CO2
R05774	12	C05528 <=>C00593 + C00011	3-Sulfopyruvate	Sulfoacetaldehyde + CO2
R03968	12	C02504 <=>C02631 + C00001	alpha-Isopropylmalate	2-Isopropylmaleate + H2O
R10117	12	C20373 <=>C20374 + C00001	3-Hydroxyglutaryl-[acp] methyl ester	Enoylglutaryl-[acp] methyl ester +H2O
R00405	12	C00002 + C00042+ C00010 <=>C00008 + C00009+ C00091	ATP + Succinate+ CoA	ADP +Orthophosphate + Succinyl-CoA
R05069	13	C06006 <=> C14463	(S)-2-Aceto-2-hydroxybutanoate	(R)-3-Hydroxy-3-methyl-2-oxopentanoate
R01058	13	C00118 + C00006 +C00001 <=>C00197 + C00005+ C00080	D-Glyceraldehyde 3-phosphate + NADP++ H2O	3-Phospho-D-glycerate + NADPH+ H+
R01197	13	2C00138+C00091+C00011+2C00080<=>2C00139+C00026+C00010	2Reducedferredoxin+Succinyl-CoA+CO2+2H+	2Oxidizedferredoxin+2-Oxoglutarate+CoA
R07159	13	C00118 + C00001 +2 C00139 <=>C00197 + 2C00080 + 2C00138	D-Glyceraldehyde 3-phosphate+ H2O+ 2 Oxidized ferredoxin	3-Phospho-D-glycerate + 2H+ +2 Reduced ferredoxin
R10118	13	C20374 + C00005+ C00080 <=>C20375 + C00006	Enoylglutaryl-[acp] methyl ester + NADPH+ H+	Glutaryl-[acp] methyl ester +NADP+
R05636	13	C00022 +C00118 <=>C11437 + C00011	Pyruvate + D-Glyceraldehyde 3-phosphate	1-Deoxy-D-xylulose 5-phosphate+ CO2
R04001	13	C02631 + C00001<=> C04411	2-Isopropylmaleate + H2O	(2R,3S)-3-Isopropylmalate
R10935	13	2C00118<=>C20954+C00009+2C00001	2D-Glyceraldehyde3-phosphate	(5-Formylfuran-3-yl)methyl phosphate +Orthophosphate +2 H2O
R01015	13	C00118 <=>C00111	D-Glyceraldehyde 3-phosphate	Glycerone phosphate
R09153	13	C00593 <=> C03576	Sulfoacetaldehyde	Coenzyme M
R00267	14	C00026 + C00011 +C00005 + C00080<=> C00311 +C00006	2-Oxoglutarate + CO2+ NADPH+ H+	Isocitrate + NADP+
R04426	14	C04411 + C00003 <=>C04236 + C00004+ C00080	(2R,3S)-3-Isopropylmalate + NAD+	(2S)-2-Isopropyl-3-oxosuccinate + NADH+ H+
R05068	14	C14463 + C00005+ C00080 <=>C06007 + C00006	(R)-3-Hydroxy-3-methyl-2-oxopentanoate + NADPH+ H+	(R)-2,3-Dihydroxy-3-methylpentanoate + NADP+
R00243	14	C00026 + C00014+ C00004 +C00080 <=>C00025 + C00003+ C00001	2-Oxoglutarate + Ammonia +NADH + H+	L-Glutamate + NAD++ H2O
R00248	14	C00026 + C00014+ C00005 +C00080 <=>C00025 + C00006+ C00001	2-Oxoglutarate + Ammonia +NADPH + H+	L-Glutamate + NADP++ H2O
R10119	14	C20375 + C01209<=> C20376+ C00011 + C00229	Glutaryl-[acp] methyl ester +Malonyl-[acyl-carrier protein]	3-Ketopimeloyl-[acp] methylester + CO2 + Acyl-carrier protein
R00271	14	C00024 + C00001+ C00026 <=>C01251 + C00010	Acetyl-CoA + H2O+ 2-Oxoglutarate	(R)-2-Hydroxybutane-1,2,4-tricarboxylate + CoA
R01070	14	C00111 + C00118 <=>C05378	Glycerone phosphate +D-Glyceraldehyde 3-phosphate	beta-D-Fructose 1,6-bisphosphate
R11038	14	C20954 + C00041 <=>C21068 + C00022	(5-Formylfuran-3-yl)methyl phosphate + L-Alanine	[5-(Aminomethyl)furan-3-yl]methyl phosphate+ Pyruvate
R10120	15	C20376 + C00005+ C00080 <=>C20377 + C00006	3-Ketopimeloyl-[acp] methyl ester + NADPH+ H+	3-Hydroxypimeloyl-[acp] methyl ester+ NADP+
R01652	15	C04236 <=>C00233 + C00011	(2S)-2-Isopropyl-3-oxosuccinate	4-Methyl-2-oxopentanoate +CO2
R00259	15	C00024 + C00025 <=>C00010 + C00624	Acetyl-CoA + L-Glutamate	CoA + N-Acetyl-L-glutamate
R00355	15	C00036 + C00025 <=>C00049 + C00026	Oxaloacetate + L-Glutamate	L-Aspartate + 2-Oxoglutarate
R00258	15	C00022 + C00025 <=>C00041 + C00026	Pyruvate + L-Glutamate	L-Alanine +2-Oxoglutarate
R04173	15	C03232 + C00025 <=>C01005 + C00026	3-Phosphonooxypyruvate + L-Glutamate	O-Phospho-L-serine + 2-Oxoglutarate
R02073	15	C00009 + C05378 <=>C00013 + C05345	Orthophosphate +beta-D-Fructose 1,6-bisphosphate	Diphosphate +beta-D-Fructose 6-phosphate
R00239	15	C00002 + C00025 <=>C00008 + C03287	ATP + L-Glutamate	ADP +L-Glutamyl 5-phosphate
R04780	15	C05378 + C00001 <=>C05345 + C00009	beta-D-Fructose 1,6-bisphosphate +H2O	beta-D-Fructose 6-phosphate +Orthophosphate
R03444	15	C01251 <=>C04002 + C00001	(R)-2-Hydroxybutane-1,2,4-tricarboxylate	(Z)-But-1-ene-1,2,4-tricarboxylate + H2O
R01900	15	C00311 <=> C00417+ C00001	Isocitrate	cis-Aconitate + H2O
R05070	15	C06007 <=>C00671 + C00001	(R)-2,3-Dihydroxy-3-methylpentanoate	(S)-3-Methyl-2-oxopentanoicacid + H2O
R00253	15	C00002 + C00025+ C00014 <=>C00008 + C00009+ C00064	ATP + L-Glutamate + Ammonia	ADP +Orthophosphate + L-Glutamine
R11039	15	C21068 + C00002 <=>C21069 + C00008	[5-(Aminomethyl)furan-3-yl]methyl phosphate+ ATP	[5-(Aminomethyl)furan-3-yl]methyl diphosphate + ADP
RMAN8	15	C05378 <=> C00354	beta-D-Fructose 1,6-bisphosphate	D-Fructose 1,6-bisphosphate
R01214_1	15	C00141 + C00025 <=>C00183 + C00026	3-Methyl-2-oxobutanoic acid + L-Glutamate	L-Valine +2-Oxoglutarate
RMAN5	15	C00025 <=> C03741	L-Glutamate	(S)-4-Amino-5-oxopentanoate
R03313	16	C03287 + C00005+ C00080 <=>C01165 + C00009 +C00006	L-Glutamyl 5-phosphate +NADPH + H+	L-Glutamate 5-semialdehyde +Orthophosphate + NADP+
R07407	16	C00049 + C00006<=> C05840+ C00005 +C00080	L-Aspartate + NADP+	Iminoaspartate + NADPH +H+
R07410	16	C00049 + C00003<=> C05840+ C00004 +C00080	L-Aspartate + NAD+	Iminoaspartate + NADH +H+
R01088	16	C00233 + C00014+ C00004 +C00080 <=>C00123 + C00001+ C00003	4-Methyl-2-oxopentanoate + Ammonia +NADH + H+	L-Leucine + H2O +NAD+
R01397	16	C00169 + C00049 <=>C00009 + C00438	Carbamoyl phosphate+ L-Aspartate	Orthophosphate +N-Carbamoyl-L-aspartate
R07274	16	C01005 + C00283 <=>C00097 + C00009	O-Phospho-L-serine + Hydrogen sulfide	L-Cysteine + Orthophosphate
R02199	16	C00671 + C00025 <=>C00407 + C00026	(S)-3-Methyl-2-oxopentanoic acid + L-Glutamate	L-Isoleucine +2-Oxoglutarate
R01090	16	C00233 + C00025 <=>C00123 + C00026	4-Methyl-2-oxopentanoate + L-Glutamate	L-Leucine +2-Oxoglutarate
R04779	16	C00002 + C05345 <=>C00008 + C05378	ATP + beta-D-Fructose 6-phosphate	ADP +beta-D-Fructose 1,6-bisphosphate
R09084	16	C05345 + C00008 <=>C05378 + C00020	beta-D-Fructose 6-phosphate + ADP	beta-D-Fructose 1,6-bisphosphate + AMP
R00764	16	C00009 + C00354 <=>C00013 + C00085	Orthophosphate +D-Fructose 1,6-bisphosphate	Diphosphate +D-Fructose 6-phosphate
R00480	16	C00002 + C00049 <=>C00008 + C03082	ATP + L-Aspartate	ADP +4-Phospho-L-aspartate
R02649	16	C00002 + C00624 <=>C00008 + C04133	ATP + N-Acetyl-L-glutamate	ADP +N-Acetyl-L-glutamate 5-phosphate
R00582	16	C01005 + C00001 <=>C00065 + C00009	O-Phospho-L-serine + H2O	L-Serine +Orthophosphate
R00489	16	C00049 <=>C00099 + C00011	L-Aspartate	beta-Alanine +CO2
R01325	16	C00417 + C00001 <=>C00158	cis-Aconitate + H2O	Citrate
R04371	16	C04002 + C00001 <=>C05662	(Z)-But-1-ene-1,2,4-tricarboxylate + H2O	Homoisocitrate
R10121	16	C20377 <=>C20378 + C00001	3-Hydroxypimeloyl-[acp] methyl ester	Enoylpimeloyl-[acp] methyl ester +H2O
R09780	16	C05345 <=> C06019	beta-D-Fructose 6-phosphate	D-arabino-Hex-3-ulose 6-phosphate
R02740	16	C05345 <=> C00668	beta-D-Fructose 6-phosphate	alpha-D-Glucose 6-phosphate
R02272	16	C03741 <=> C00430	(S)-4-Amino-5-oxopentanoate	5-Aminolevulinate
R00483	16	C00002 + C00049+ C00014 <=>C00020 + C00013+ C00152	ATP + L-Aspartate + Ammonia	AMP +Diphosphate + L-Asparagine
R00578	16	C00002 + C00049+ C00064 +C00001 <=>C00020 + C00013+ C00152 +C00025	ATP + L-Aspartate +L-Glutamine +H2O	AMP +Diphosphate + L-Asparagine + L-Glutamate
R00575	16	2C00002+C00064+C00288+C00001<=>2C00008+C00009+C00025+C00169	2ATP+L-Glutamine+HCO3-+H2O	2ADP+Orthophosphate+L-Glutamate+Carbamoylphosphate
R00482	16	C00002 + C00049+ C00014 <=>C00008 + C00009+ C00152	ATP + L-Aspartate + Ammonia	ADP +Orthophosphate + L-Asparagine
RMAN7	16	C05345 <=> C00085	beta-D-Fructose 6-phosphate	D-Fructose 6-phosphate
R00400	16	C00022 + C00049 <=>C00041 + C00036	Pyruvate + L-Aspartate	L-Alanine +Oxaloacetate
R01214_2	16	C00183 + C00026 <=>C00141 + C00025	L-Valine +2-Oxoglutarate	3-Methyl-2-oxobutanoic acid + L-Glutamate
R01934	17	C05662 + C00003<=> C00322+ C00011 + C00004+ C00080	Homoisocitrate + NAD+	2-Oxoadipate +CO2 + NADH+ H+
R02291	17	C03082 + C00005+ C00080 <=>C00441 + C00009+ C00006	4-Phospho-L-aspartate + NADPH+ H+	L-Aspartate 4-semialdehyde +Orthophosphate + NADP+
R03443	17	C04133 + C00005+ C00080 <=>C01250 + C00009+ C00006	N-Acetyl-L-glutamate 5-phosphate +NADPH +H+	N-Acetyl-L-glutamate 5-semialdehyde +Orthophosphate + NADP+
R10122	17	C20378 + C00005+ C00080 <=>C19846 + C00006	Enoylpimeloyl-[acp] methyl ester + NADPH+ H+	Pimeloyl-[acyl-carrier protein] methyl ester + NADP+
R00945	17	C00101 + C00065<=> C00143+ C00037 +C00001	Tetrahydrofolate + L-Serine	5,10-Methylenetetrahydrofolate +Glycine + H2O
R00586	17	C00065 + C00024 <=>C00979 + C00010	L-Serine + Acetyl-CoA	O-Acetyl-L-serine + CoA
R00352	17	C00002 + C00158+ C00010 <=>C00008 + C00009+ C00024 +C00036	ATP + Citrate+ CoA	ADP +Orthophosphate + Acetyl-CoA +Oxaloacetate
R04292	17	C05840 + C00111 <=>C03722 + 2C00001 + C00009	Iminoaspartate +Glycerone phosphate	Quinolinate +2 H2O + Orthophosphate
R00756	17	C00002 + C00085 <=>C00008 + C00354	ATP + D-Fructose 6-phosphate	ADP +D-Fructose 1,6-bisphosphate
R05805	17	C00008 + C00085 <=>C00020 + C00354	ADP +D-Fructose 6-phosphate	AMP +D-Fructose 1,6-bisphosphate
R12342	17	C00008 + C00065 <=>C00020 + C01005	ADP + L-Serine	AMP +O-Phospho-L-serine
R07460	17	C15811 + C00097 <=>C15812 + C00041	[Enzyme]-cysteine +L-Cysteine	[Enzyme]-S-sulfanylcysteine + L-Alanine
R01993	17	C00438 <=>C00337 + C00001	N-Carbamoyl-L-aspartate	(S)-Dihydroorotate + H2O
R05338	17	C06019 <=>C00199 + C00067	D-arabino-Hex-3-ulose 6-phosphate	D-Ribulose 5-phosphate +Formaldehyde
R00036	17	2C00430<=>C00931+2C00001	25-Aminolevulinate	Porphobilinogen + 2 H2O
R02739	17	C00668 <=>C01172	alpha-D-Glucose 6-phosphate	beta-D-Glucose 6-phosphate
R00959	17	C00668 <=> C00103	alpha-D-Glucose 6-phosphate	D-Glucose 1-phosphate
R02473	17	C00002 + C00522+ C00099 <=>C00020 + C00013+ C00864	ATP + (R)-Pantoate + beta-Alanine	AMP + Diphosphate +Pantothenate
R01322	17	C00002 + C00158+ C00010 <=>C00008 + C00009+ C00566	ATP + Citrate+ CoA	ADP +Orthophosphate + (3S)-Citryl-CoA
R01788	17	C00668 + C00001 <=>C00267 + C00009	alpha-D-Glucose 6-phosphate +H2O	alpha-D-Glucose + Orthophosphate
R03314	17	C01165 <=> C03912+ C00001	L-Glutamate 5-semialdehyde	(S)-1-Pyrroline-5-carboxylate + H2O
R01773	18	C00441 + C00004+ C00080 <=>C00263 + C00003	L-Aspartate 4-semialdehyde +NADH + H+	L-Homoserine + NAD+
R01775	18	C00441 + C00005+ C00080 <=>C00263 + C00006	L-Aspartate 4-semialdehyde +NADPH + H+	L-Homoserine + NADP+
R10907	18	C01172 + C00003 <=>C01236 + C00004+ C00080	beta-D-Glucose 6-phosphate +NAD+	D-Glucono-1,5-lactone 6-phosphate +NADH +H+
R02736	18	C01172 + C00006 <=>C01236 + C00005+ C00080	beta-D-Glucose 6-phosphate +NADP+	D-Glucono-1,5-lactone 6-phosphate +NADPH +H+
R01869	18	C00337 + C00003<=> C00295+ C00080 +C00004	(S)-Dihydroorotate + NAD+	Orotate + H++ NADH
R01867	18	C00337 + C00122 <=>C00295 + C00042	(S)-Dihydroorotate + Fumarate	Orotate + Succinate
R01248	18	C03912 + C00004+ C00080 <=>C00148 + C00003	(S)-1-Pyrroline-5-carboxylate + NADH+ H+	L-Proline + NAD+
R01251	18	C03912 + C00005+ C00080 <=>C00148 + C00006	(S)-1-Pyrroline-5-carboxylate + NADPH+ H+	L-Proline + NADP+
R00371	18	C00024 + C00037 <=>C00010 + C03508	Acetyl-CoA +Glycine	CoA + L-2-Amino-3-oxobutanoic acid
R00897	18	C00979 + C00283 <=>C00097 + C00033	O-Acetyl-L-serine + Hydrogen sulfide	L-Cysteine + Acetate
R00084	18	4C00931+C00001<=>C01024+4C00014	4Porphobilinogen+H2O	Hydroxymethylbilane + 4 Ammonia
R00369	18	C00022 + C00037 <=>C00041 + C00048	Pyruvate + Glycine	L-Alanine +Glyoxylate
R01786	18	C00002 + C00267 <=>C00008 + C00668	ATP + alpha-D-Glucose	ADP +alpha-D-Glucose 6-phosphate
R09085	18	C00267 + C00008 <=>C00668 + C00020	alpha-D-Glucose + ADP	alpha-D-Glucose 6-phosphate + AMP
R03018	18	C00002 + C00864 <=>C00008 + C03492	ATP + Pantothenate	ADP + D-4'-Phosphopantothenate
R09725	18	C19846 + C00001 <=>C19845 + C00132	Pimeloyl-[acyl-carrier protein] methyl ester +H2O	Pimeloyl-[acyl-carrier protein] + Methanol
R00947	18	C00103 + C00001 <=>C00267 + C00009	D-Glucose 1-phosphate +H2O	alpha-D-Glucose + Orthophosphate
R00354	18	C00566 <=>C00024 + C00036	(3S)-Citryl-CoA	Acetyl-CoA + Oxaloacetate
R07281	18	C00199 <=>C15556 + C00058	D-Ribulose 5-phosphate	L-3,4-Dihydroxybutan-2-one 4-phosphate +Formate
R10147	18	C00441 + C00022 <=>C20258 + C00001	L-Aspartate 4-semialdehyde+ Pyruvate	(2S,4S)-4-Hydroxy-2,3,4,5-tetrahydrodipicolinate + H2O
R01529	18	C00199 <=> C00231	D-Ribulose 5-phosphate	D-Xylulose 5-phosphate
R01602	18	C00267 <=> C00221	alpha-D-Glucose	beta-D-Glucose
R01056	18	C00199 <=>C00117	D-Ribulose 5-phosphate	D-Ribose 5-phosphate
R08331	18	C00322 + C00024+ C00001 <=>C16583 + C00010	2-Oxoadipate + Acetyl-CoA+ H2O	(R)-(Homo)2-citrate + CoA
RMAN9	18	C00267 <=> C00031	alpha-D-Glucose	D-glucose
R02283	18	C01250 + C00025 <=>C00437 + C00026	N-Acetyl-L-glutamate 5-semialdehyde +L-Glutamate	N-Acetylornithine +2-Oxoglutarate
R00372	19	C00048 + C00025 <=>C00037 + C00026	Glyoxylate + L-Glutamate	Glycine + 2-Oxoglutarate
R03758	19	C03508 <=>C01888 + C00011	L-2-Amino-3-oxobutanoic acid	Aminoacetone + CO2
R04198	19	C20258 + C00004+ C00080 <=>C03972 + C00003+ C00001	(2S,4S)-4-Hydroxy-2,3,4,5-tetrahydrodipicolinate + NADH+ H+	2,3,4,5-Tetrahydrodipicolinate + NAD++ H2O
R04199	19	C20258 + C00005+ C00080 <=>C03972 + C00006+ C00001	(2S,4S)-4-Hydroxy-2,3,4,5-tetrahydrodipicolinate + NADPH+ H+	2,3,4,5-Tetrahydrodipicolinate + NADP++ H2O
R07245	19	C00169 + C00437 <=>C00009 + C15532	Carbamoyl phosphate +N-Acetylornithine	Orthophosphate + N-Acetyl-L-citrulline
R01641	19	C00117 + C00231<=> C05382+ C00118	D-Ribose 5-phosphate +D-Xylulose 5-phosphate	Sedoheptulose 7-phosphate+ D-Glyceraldehyde3-phosphate
R01777	19	C00091 +C00263 <=>C00010 +C01118	Succinyl-CoA + L-Homoserine	CoA + O-Succinyl-L-homoserine
R09086	19	C00221 + C00008<=> C01172 +C00020	beta-D-Glucose + ADP	beta-D-Glucose 6-phosphate + AMP
R05804	19	C00008 + C00031 <=>C00020 + C00092	ADP +D-Glucose	AMP +D-Glucose 6-phosphate
R00299	19	C00002 + C00031 <=>C00008 + C00092	ATP +D-Glucose	ADP +D-Glucose 6-phosphate

R01600	19	C00002 + C00221 <=> C00008 + C01172	ATP + beta-D-Glucose	ADP + beta-D-Glucose 6-phosphate
R01771	19	C00002 + C00263 <=> C00008 + C01102	ATP + L-Homoserine	ADP + O-Phospho-L-homoserine
R01049	19	C00002 + C00117 <=> C00020 + C00119	ATP + D-Ribose 5-phosphate	AMP + 5-Phospho-alpha-D-ribose 1-diphosphate
R02035	19	C01236 + C00001 <=> C00345	D-Glucono-1,5-lactone 6-phosphate + H2O	6-Phospho-D-gluconate
R00669	19	C00437 + C00001 <=> C00033 + C00077	N-Acetylornithine + H2O	Acetate + L-Ornithine
R10392	19	C16583 <=> C20581 + C00001	(R)-(Homo)2-citrate	cis-(Homo)2-aconitate + H2O
R03165	19	C01024 <=> C01051 + C00001	Hydroxymethylbilane	Uroporphyrinogen III + H2O
R10089	19	C00117 + C00118 + C00064 <=> C00018 + C00025 + 3 C00001 + C00009	D-Ribose 5-phosphate + D-Glyceraldehyde 3-phosphate + L-Glutamine	Pyridoxal phosphate + L-Glutamate + 3H2O + Orthophosphate
R04230	19	C00002 + C03492 + C00097 <=> C00020 + C00013 + C04352	ATP + D-4'-Phosphopantothenate + L-Cysteine	AMP + Diphosphate + (R)-4'-Phosphopantothenoyl-L-cysteine
R04231	19	C00063 + C03492 + C00097 <=> C00055 + C00013 + C04352	CTP + D-4'-Phosphopantothenate + L-Cysteine	CMP + Diphosphate + (R)-4'-Phosphopantothenoyl-L-cysteine
RMAN6	19	C00221 <=> C00031	beta-D-Glucose	D-glucose
R10124	19	C19845 + C00041 <=> C01092 + C00229 + C00011	Pimeloyl-[acyl-carrier protein] + L-Alanine	8-Amino-7-oxononanoate + Acyl-carrier protein + CO2
R10221	20	C00345 + C00003 <=> C00199 + C00011 + C00004 + C00080	6-Phospho-D-gluconate + NAD+	D-Ribulose 5-phosphate + CO2 + NADH + H+
R01528	20	C00345 + C00006 <=> C00199 + C00011 + C00005 + C00080	6-Phospho-D-gluconate + NADP+	D-Ribulose 5-phosphate + CO2 + NADPH + H+
R03759	20	C01888 + C00004 + C00080 <=> C03194 + C00003	Aminoacetone + NADH + H+	(R)-1-Aminopropan-2-ol + NAD+
R01398	20	C00169 + C00077 <=> C00009 + C00327	Carbamoyl phosphate + L-Ornithine	Orthophosphate + L-Citrulline
R01827	20	C05382 + C00118 <=> C00279 + C05345	Sedoheptulose 7-phosphate + D-Glyceraldehyde3-phosphate	D-Erythrose 4-phosphate + beta-D-Fructose 6-phosphate
R04365	20	C00091 + C03972 + C00001 <=> C00010 + C04462	Succinyl-CoA + 2,3,4,5-Tetrahydrodipicolinate + H2O	CoA + N-Succinyl-2-L-amino-6-oxoheptanedioate
R04364	20	C03972 + C00024 + C00001 <=> C05539 + C00010	2,3,4,5-Tetrahydrodipicolinate + Acetyl-CoA + H2O	N-Acetyl-L-2-amino-6-oxopimelate + CoA
R01870	20	C00295 + C00119 <=> C01103 + C00013	Orotate + 5-Phospho-alpha-D-ribose 1-diphosphate	Orotidine 5'-phosphate + Diphosphate
R01072	20	C00064 + C00119 + C00001 <=> C03090 + C00013 + C00025	L-Glutamine + 5-Phospho-alpha-D-ribose 1-diphosphate + H2O	5-Phosphoribosylamine + Diphosphate + L-Glutamate
R03348	20	C03722 + C00119 <=> C01185 + C00013 + C00011	Quinolinate + 5-Phospho-alpha-D-ribose 1-diphosphate	Nicotinate D-ribonucleotide + Diphosphate + CO2
R03260	20	C01118 + C00097 <=> C02291 + C00042	O-Succinyl-L-homoserine + L-Cysteine	L-Cystathionine + Succinate
R00667	20	C00077 + C00026 <=> C01165 + C00025	L-Ornithine + 2-Oxoglutarate	L-Glutamate 5-semialdehyde + L-Glutamate
R07613	20	C03972 + C00025 + C00001 <=> C00666 + C00026	2,3,4,5-Tetrahydrodipicolinate + L-Glutamate + H2O	LL-2,6-Diaminoheptanedioate + 2-Oxoglutarate
R09107	20	C15532 + C00001 <=> C00033 + C00327	N-Acetyl-L-citrulline + H2O	Acetate + L-Citrulline
R03269	20	C04352 <=> C01134 + C00011	(R)-4'-Phosphopantothenoyl-L-cysteine	Pantetheine 4'-phosphate + CO2
R10393	20	C20581 + C00001 <=> C16597	cis-(Homo)2-aconitate + H2O	(-)-threo-Iso(homo)2-citrate
R01466	20	C01102 + C00001 <=> C00188 + C00009	O-Phospho-L-homoserine + H2O	L-Threonine + Orthophosphate
R04336	20	C03972 + C00001 <=> C03871	2,3,4,5-Tetrahydrodipicolinate + H2O	L-2-Amino-6-oxoheptanedioate
R01465	21	C00188 + C00003 <=> C03508 + C00004 + C00080	L-Threonine + NAD+	L-2-Amino-3-oxobutanoic acid + NADH + H+
R03947	21	C02463 + C00003 <=> C05778 + C00004 + C00080	Precorrin 2 + NAD+	Sirohydrochlorin + NADH + H+
R02755	21	C03871 + C00014 + C00005 + C00080 <=> C00680 + C00006 + C00001	L-2-Amino-6-oxoheptanedioate + Ammonia + NADPH + H+	meso-2,6-Diaminoheptanedioate + NADP+ + H2O
R01830	21	C00279 + C00231 <=> C05345 + C00118	D-Erythrose 4-phosphate + D-Xylulose 5-phosphate	beta-D-Fructose 6-phosphate + D-Glyceraldehyde 3-phosphate
R01826	21	C00074 + C00279 + C00001 <=> C04691 + C00009	Phosphoenolpyruvate + D-Erythrose 4-phosphate + H2O	2-Dehydro-3-deoxy-D-arabino-heptonate 7-phosphate + Orthophosphate
R04475	21	C04462 + C00025 <=> C04421 + C00026	N-Succinyl-2-L-amino-6-oxoheptanedioate + L-Glutamate	N-Succinyl-LL-2,6-diaminoheptanedioate + 2-Oxoglutarate
R00965	21	C01103 <=> C00105 + C00011	Orotidine 5'-phosphate	UMP + CO2
R00751	21	C00188 <=> C00037 + C00084	L-Threonine	Glycine + Acetaldehyde
R00996	21	C00188 <=> C00109 + C00014	L-Threonine	2-Oxobutanoate + Ammonia
R01001	21	C02291 + C00001 <=> C00097 + C00014 + C00109	L-Cystathionine + H2O	L-Cysteine + Ammonia + 2-Oxobutanoate
R01286	21	C02291 + C00001 <=> C00155 + C00014 + C00022	L-Cystathionine + H2O	L-Homocysteine + Ammonia + Pyruvate
R02735	21	C00666 <=> C00680	LL-2,6-Diaminoheptanedioate	meso-2,6-Diaminoheptanedioate
R04144	21	C00002 + C03090 + C00037 <=> C00008 + C00009 + C03838	ATP + 5-Phosphoribosylamine + Glycine	ADP + Orthophosphate + 5'-Phosphoribosylglycinamide
R01954	21	C00002 + C00327 + C00049 <=> C00020 + C00013 + C03406	ATP + L-Citrulline + L-Aspartate	AMP + Diphosphate + N-(L-Arginino)succinate
R04467	21	C05539 + C00025 <=> C04390 + C00026	N-Acetyl-L-2-amino-6-oxopimelate + L-Glutamate	N6-Acetyl-LL-2,6-diaminoheptanedioate + 2-Oxoglutarate
R08214	21	C16597 + C00003 <=> C16588 + C00011 + C00004 + C00080	(-)-threo-Iso(homo)2-citrate + NAD+	2-Oxopimelate + CO2 + NADH + H+
R00946	22	C00440 + C00155 <=> C00101 + C00073	5-Methyltetrahydrofolate + L-Homocysteine	Tetrahydrofolate + L-Methionine
R00158	22	C00002 + C00105 <=> C00008 + C00015	ATP + UMP	ADP + UDP
R02734	22	C04421 + C00001 <=> C00042 + C00666	N-Succinyl-LL-2,6-diaminoheptanedioate + H2O	Succinate + LL-2,6-Diaminoheptanedioate
R00451	22	C00680 <=> C00047 + C00011	meso-2,6-Diaminoheptanedioate	L-Lysine + CO2
R01290	22	C00065 + C00155 <=> C02291 + C00001	L-Serine + L-Homocysteine	L-Cystathionine + H2O
R03083	22	C04691 <=> C00944 + C00009	2-Dehydro-3-deoxy-D-arabino-heptonate 7-phosphate	3-Dehydroquinate + Orthophosphate
R01086	22	C03406 <=> C00122 + C00062	N-(L-Arginino)succinate	Fumarate + L-Arginine
R11626	22	C05778 + C19609 <=> C21510 + 2 C00080	Sirohydrochlorin + Nickel(2+)	Nickel-sirohydrochlorin + 2 H+
R05807	22	C05778 + C00175 <=> C11538 + 2 C00080	Sirohydrochlorin + Cobalt ion	Cobalt-sirohydrochlorin + 2 H+
R08332	22	C16588 + C00024 + C00001 <=> C16598 + C00010	2-Oxopimelate + Acetyl-CoA + H2O	(R)-(Homo)3-citrate + CoA
R10305	22	C00979 + C00155 <=> C02291 + C00033	O-Acetyl-L-serine + L-Homocysteine	L-Cystathionine + Acetate
R02733	22	C04390 + C00001 <=> C00033 + C00666	N6-Acetyl-LL-2,6-diaminoheptanedioate + H2O	Acetate + LL-2,6-Diaminoheptanedioate
R04325	22	C00234 + C03838 <=> C00101 + C04376	10-Formyltetrahydrofolate + 5'-Phosphoribosylglycinamide	Tetrahydrofolate + 5'-Phosphoribosyl-N-formylglycinamide
R10699	23	C00047 + C01092 <=> C04076 + C01037	L-Lysine + 8-Amino-7-oxononanoate	L-2-Aminoadipate 6-semialdehyde + 7,8-Diaminononanoate
R00156	23	C00002 + C00015 <=> C00008 + C00075	ATP + UDP	ADP + UTP
R00551	23	C00062 + C00001 <=> C00077 + C00086	L-Arginine + H2O	L-Ornithine + Urea
R03084	23	C00944 <=> C02637 + C00001	3-Dehydroquinate	3-Dehydroshikimate + H2O
R10395	23	C16598 <=> C20582 + C00001	(R)-(Homo)3-citrate	cis-(Homo)3-aconitate + H2O
R11627	23	C21510 + 2 C00002 + 2 C00064 + 2 C00001 <=> C21511 + 2 C00008 + 2 C00025 + 2 C00009	Nickel-sirohydrochlorin + 2 ATP + 2 L-Glutamine + 2 H2O	Nickel-sirohydrochlorin a,c-diamide + 2 ADP + 2 L-Glutamate + 2 Orthophosphate
R04463	23	C00002 + C04376 + C00064 + C00001 <=> C00008 + C00009 + C04640 + C00025	ATP + 5'-Phosphoribosyl-N-formylglycinamide + L-Glutamine + H2O	ADP + Orthophosphate + 2-(Formamido)-N1-(5'-phosphoribosyl)acetamidine + L-Glutamate
R02413	24	C02637 + C00005 + C00080 <=> C00493 + C00006	3-Dehydroshikimate + NADPH + H+	Shikimate + NADP+
R10396	24	C20582 + C00001 <=> C16600	cis-(Homo)3-aconitate + H2O	(-)-threo-Iso(homo)3-citrate
R04208	24	C00002 + C04640 <=> C00008 + C00009 + C03373	ATP + 2-(Formamido)-N1-(5'-phosphoribosyl)acetamidine	ADP + Orthophosphate + Aminoimidazole ribotide
R03182	24	C00002 + C01037 + C00011 <=> C00008 + C00009 + C01909	ATP + 7,8-Diaminononanoate + CO2	ADP + Orthophosphate + Dethiobiotin
R11628	24	C00002 + C21511 + 3 C00030 + C00001 <=> C00008 + C00009 + C21512 + 3 C00028	ATP + Nickel-sirohydrochlorin a,c-diamide + 3Reduced acceptor + H2O	ADP + Orthophosphate + 15,17(3)-Seco-F430-17(3)-acid + 3 Acceptor
R00571	24	C00002 + C00075 + C00014 <=> C00008 + C00009 + C00063-SYN	ATP + UTP + Ammonia	ADP + Orthophosphate + CTP
R00573	24	C00002 + C00075 + C00064 + C00001 <=> C00008 + C00009 + C00063-SYN + C00025	ATP + UTP + L-Glutamine + H2O	ADP + Orthophosphate + CTP + L-Glutamate
R08716	24	C17401 <=> C11539	Cobalt-factorIII	Cobalt-precorrin 3
R02412	25	C00002 + C00493 <=> C00008 + C03175	ATP + Shikimate	ADP + Shikimate 3-phosphate
R04209	25	C03373 + C00011 <=> C04751	Aminoimidazole ribotide + CO2	1-(5-Phospho-D-ribosyl)-5-amino-4-imidazolecarboxylate
R07404	25	C00002 + C03373 + C00288 <=> C00008 + C00009 + C15667	ATP + Aminoimidazole ribotide + HCO3-	ADP + Orthophosphate + 5-Carboxyamino-1-(5-phospho-D-ribosyl)imidazole
R11629	25	C21512 + C00002 <=> C05777 + C00008 + C00009	15,17(3)-Seco-F430-17(3)-acid + ATP	Coenzyme F430 + ADP + Orthophosphate
R08215	25	C16600 + C00003 <=> C16589 + C00011 + C00004 + C00080	(-)-threo-Iso(homo)3-citrate + NAD+	2-Oxosuberate + CO2 + NADH + H+
R11636	25	C00063-SYN + C00058 <=> C00458 + C00011 + C00001	CTP + Formate	dCTP + CO2 + H2O
R03460	26	C00074 + C03175 <=> C00009 + C01269	Phosphoenolpyruvate + Shikimate 3-phosphate	Orthophosphate + 5-O-(1-Carboxyvinyl)-3-phosphoshikimate
R02325	26	C00458 + C00001 <=> C00460 + C00014	dCTP + H2O	dUTP + Ammonia
R07772	26	C16242 + C00001 <=> C16243 + C00084	Cobalt-precorrin 5A + H2O	Cobalt-precorrin 5B + Acetaldehyde
R07405	26	C15667 <=> C04751	5-Carboxyamino-1-(5-phospho-D-ribosyl)imidazole	1-(5-Phospho-D-ribosyl)-5-amino-4-imidazolecarboxylate
R04591	26	C00002 + C04751 + C00049 <=> C00008 + C00009 + C04823	ATP + 1-(5-Phospho-D-ribosyl)-5-amino-4-imidazolecarboxylate + L-Aspartate	ADP + Orthophosphate + 1-(5'-Phosphoribosyl)-5-amino-4-(N-succinocarboxamide)-imidazole
R08328	26	C16589 <=> C16590 + C00011	2-Oxosuberate	7-Oxoheptanoicacid + CO2
R02100	27	C00460 + C00001 <=> C00365 + C00013	dUTP + H2O	dUMP + Diphosphate
R01714	27	C01269 <=> C00251 + C00009	5-O-(1-Carboxyvinyl)-3-phosphoshikimate	Chorismate + Orthophosphate
R04559	27	C04823 <=> C00122 + C04677	1-(5'-Phosphoribosyl)-5-amino-4-(N-succinocarboxamide)-imidazole	Fumarate + 1-(5'-Phosphoribosyl)-5-amino-4-imidazolecarboxamide
R10397	27	C16590 <=> C16593	7-Oxoheptanoic acid	7-Mercaptoheptanoic acid
R05812	28	C11542 + C00004 + C00080 <=> C11543 + C00003	Cobalt-precorrin 6 + NADH + H+	Cobalt-dihydro-precorrin 6 + NAD+
R02101	28	C00365 + C00143 <=> C00415 + C00364	dUMP + 5,10-Methylenetetrahydrofolate	Dihydrofolate + dTMP
R04560	28	C00234 + C04677 <=> C00101 + C04734	10-Formyltetrahydrofolate + 1-(5'-Phosphoribosyl)-5-amino-4-imidazolecarboxamide	Tetrahydrofolate + 1-(5'-Phosphoribosyl)-5-formamido-4-imidazolecarboxamide
R01716	28	C00251 + C00064 <=> C11355 + C00025	Chorismate + L-Glutamine	4-Amino-4-deoxychorismate + L-Glutamate
R00985	28	C00251 + C00014 <=> C00108 + C00022 + C00001	Chorismate + Ammonia	Anthranilate + Pyruvate + H2O
R00986	28	C00251 + C00064 <=> C00108 + C00022 + C00025	Chorismate + L-Glutamine	Anthranilate + Pyruvate + L-Glutamate
R01302	28	C00251 <=> C00156 + C00022	Chorismate	4-Hydroxybenzoate + Pyruvate
R01715	28	C00251 <=> C00254	Chorismate	Prephenate
R06975	28	C00058 + C00002 + C04677 <=> C00008 + C00009 + C04734	Formate + ATP + 1-(5'-Phosphoribosyl)-5-amino-4-imidazolecarboxamide	ADP + Orthophosphate + 1-(5'-Phosphoribosyl)-5-formamido-4-imidazolecarboxamide
R08323	28	C16593 + C00188 + C00002 <=> C16594 + C00008 + C00009	7-Mercaptoheptanoic acid + L-Threonine + ATP	7-Mercaptoheptanoylthreonine + ADP + Orthophosphate
R01728	29	C00254 + C00003 <=> C01179 + C00011 + C00004 + C00080	Prephenate + NAD+	3-(4-Hydroxyphenyl)pyruvate + CO2 + NADH + H+
R00936	29	C00415 + C00004 + C00080 <=> C00101-SYN + C00003	Dihydrofolate + NADH + H+	Tetrahydrofolate + NAD+
R00939	29	C00415 + C00005 + C00080 <=> C00101-SYN + C00006	Dihydrofolate + NADPH + H+	Tetrahydrofolate + NADP+
R01073	29	C00108 + C00119 <=> C04302 + C00013	Anthranilate + 5-Phospho-alpha-D-ribose 1-diphosphate	N-(5-Phospho-D-ribosyl)anthranilate + Diphosphate
R11102	29	C00119 + C00156 <=> C21107 + C00011 + C00013	5-Phospho-alpha-D-ribose 1-diphosphate + 4-Hydroxybenzoate	4-(beta-D-Ribofuranosyl)phenol 5'-phosphate + CO2 + Diphosphate
R01731	29	C00049 + C00254 <=> C00036 + C00826	L-Aspartate + Prephenate	Oxaloacetate + L-Arogenate
R07276	29	C00254 + C00025 <=> C00826 + C00026	Prephenate + L-Glutamate	L-Arogenate + 2-Oxoglutarate
R02094	29	C00002 + C00364 <=> C00008 + C00363	ATP + dTMP	ADP + dTDP
R01127	29	C04734 <=> C00130 + C00001	1-(5'-Phosphoribosyl)-5-formamido-4-imidazolecarboxamide	IMP + H2O
R05553	29	C11355 <=> C00568 + C00022	4-Amino-4-deoxychorismate	4-Aminobenzoate + Pyruvate
R01373	29	C00254 <=> C00166 + C00001 + C00011	Prephenate	Phenylpyruvate + H2O + CO2
R08217	29	C16594 + C00002 <=> C04628 + C00008	7-Mercaptoheptanoylthreonine + ATP	Coenzyme B + ADP
R01730	29	C00254 + C00006 <=> C01179 + C00011 + C00005 + C00080	Prephenate + NADP+	3-(4-Hydroxyphenyl)pyruvate + CO2 + NADPH + H+
R01130	30	C00130 + C00003 + C00001 <=> C00655 + C00004 + C00080	IMP + NAD+ + H2O	Xanthosine 5'-phosphate + NADH + H+
R00694	30	C00166 + C00025 <=> C00079 + C00026	Phenylpyruvate + L-Glutamate	L-Phenylalanine + 2-Oxoglutarate
R00734	30	C01179 + C00025 <=> C00082 + C00026	3-(4-Hydroxyphenyl)pyruvate + L-Glutamate	L-Tyrosine + 2-Oxoglutarate
R02093	30	C00002 + C00363 <=> C00008 + C00459	ATP + dTDP	ADP + dTTP
R00691	30	C00826 <=> C00079 + C00001 + C00011	L-Arogenate	L-Phenylalanine + H2O + CO2
R03509	30	C04302 <=> C01302	N-(5-Phospho-D-ribosyl)anthranilate	1-(2-Carboxyphenylamino)-1-deoxy-D-ribulose 5-phosphate
R01135	30	C00044 + C00130 + C00049 <=> C00035 + C00009 + C03794	GTP + IMP + L-Aspartate	GDP + Orthophosphate + N6-(1,2-Dicarboxyethyl)-AMP
R00732	30	C00826 + C00003 <=> C00082 + C00011 + C00004 + C00080	L-Arogenate + NAD+	L-Tyrosine + CO2 + NADH + H+
R00733	30	C00826 + C00006 <=> C00082 + C00011 + C00005 + C00080	L-Arogenate + NADP+	L-Tyrosine + CO2 + NADPH + H+
RMAN1	30	C21107 <=> C20559	4-(beta-D-Ribofuranosyl)phenol 5'-phosphate	4-(beta-D-Ribofuranosyl)aniline 5'-phosphate
R00736	31	C00082 <=> C00483 + C00011	L-Tyrosine	Tyramine + CO2
R03508	31	C01302 <=> C03506 + C00011 + C00001	1-(2-Carboxyphenylamino)-1-deoxy-D-ribulose 5-phosphate	Indoleglycerol phosphate + CO2 + H2O
R01083	31	C03794 <=> C00122 + C00020-SYN	N6-(1,2-Dicarboxyethyl)-AMP	Fumarate + AMP
R05814	31	C11545 <=> C05773	Cobalt-precorrin 8	Cobyrinate
R01230	31	C00002 + C00655 + C00014 <=> C00020 + C00013 + C00144-SYN	ATP + Xanthosine 5'-phosphate + Ammonia	AMP + Diphosphate + GMP
R01231	31	C00002 + C00655 + C00064 + C00001 <=> C00020 + C00013 + C00144-SYN + C00025	ATP + Xanthosine 5'-phosphate + L-Glutamine + H2O	AMP + Diphosphate + GMP + L-Glutamate
R00127	32	C00002 + C00020-SYN <=> 2 C00008-SYN	ATP + AMP	2 ADP
R00332	32	C00002 + C00144-SYN <=> C00008 + C00035-SYN	ATP + GMP	ADP + GDP
R02722	32	C00065 + C03506 <=> C00078 + C00118 + C00001	L-Serine + Indoleglycerol phosphate	L-Tryptophan + D-Glyceraldehyde 3-phosphate + H2O
R05815	32	C05773 + 2 C00064 + 2 C00002 + 2 C00001 <=> C06504 + 2 C00025 + 2 C00008 + 2 C00009	Cobyrinate + 2 L-Glutamine + 2 ATP + 2 H2O	Cob(II)yrinate a,c diamide + 2 L-Glutamate + 2 ADP + 2 Orthophosphate
R10902	32	C00002 + C00483 + C00025 <=> C00008 + C00009 + C20926	ATP + Tyramine + L-Glutamate	ADP + Orthophosphate + gamma-Glutamyltyramine
R00200	33	C00008-SYN + C00074 <=> C00002-SYN + C00022	ADP + Phosphoenolpyruvate	ATP + Pyruvate
R00430	33	C00035-SYN + C00074 <=> C00044-SYN + C00022	GDP + Phosphoenolpyruvate	GTP + Pyruvate
R00315	33	C00008-SYN + C00227 <=> C00002-SYN + C00033	ADP + Acetyl phosphate	ATP + Acetate
R00330	33	C00002 + C00035-SYN <=> C00008 + C00044-SYN	ATP + GDP	ADP + GTP
R11040	33	C21069 + C20926 <=> C21070 + C00013	[5-(Aminomethyl)furan-3-yl]methyl diphosphate + gamma-Glutamyltyramine	(4-{4-[2-(gamma-L-Glutamylamino)ethyl]phenoxymethyl}furan-2-yl)methanamine + Diphosphate
R05218	33	2C06504+C01847<=>2C06505+C00061+2C00080	2 Cob(II)yrinate a,c diamide + Reduced FMN	2 Cob(I)yrinate a,c diamide + FMN + 2 H+
R05705	34	C00061 + C00004 + C00080 <=> C01847 + C00003	FMN + NADH + H+	Reduced FMN + NAD+
R05706	34	C00061 + C00005 + C00080 <=> C01847 + C00006	FMN + NADPH + H+	Reduced FMN + NADP+
R01071	34	C00002-SYN + C00119 <=> C02739 + C00013	ATP + 5-Phospho-alpha-D-ribose 1-diphosphate	1-(5-Phospho-D-ribosyl)-ATP + Diphosphate
R05220	34	C06505 + C00002-SYN <=> C06506 + C00536	Cob(I)yrinate a,c diamide + ATP	Adenosyl cobyrinate a,c diamide + Triphosphate
R00177	34	C00002-SYN + C00073 + C00001 <=> C00009 + C00013 + C00019-SYN	ATP + L-Methionine + H2O	Orthophosphate + Diphosphate + S-Adenosyl-L-methionine
R03005	34	C00002-SYN + C01185 <=> C00013 + C00857	ATP + Nicotinate D-ribonucleotide	Diphosphate + Deamino-NAD+
R03035	34	C00002-SYN + C01134 <=> C00013 + C00882	ATP + Pantetheine 4'-phosphate	Diphosphate + Dephospho-CoA
R07459	34	C15810 + C00002-SYN <=> C15813 + C00013	Sulfur-carrierprotein + ATP	Adenylyl-[sulfur-carrier protein] + Diphosphate
R00428	34	C00044-SYN + C00001 <=> C05922	GTP + H2O	Formamidopyrimidine nucleoside triphosphate
R00425	34	C00044-SYN + 3 C00001 <=> C00058 + C01304 + C00013	GTP + 3 H2O	Formate + 2,5-Diamino-6-(5-phospho-D-ribosylamino)pyrimidin-4(3H)-one + Diphosphate
RMAN10	34	C00074 + C00044-SYN <=> C22297 + C00013	PEP + GTP	Enolpyruvoyl-2-diphospho-5'-guanosine + Diphosphate
RMAN3	34	C21070 <=> C00862-SYN	(4-{4-[2-(gamma-L-Glutamylamino)ethyl]phenoxymethyl}furan-2-yl)methanamine	Methanofuran
R11633	34	C00044-SYN + C00058 <=> C00286 + C00011 + C00001	GTP + Formate	dGTP + CO2 + H2O
R11634	34	C00002-SYN + C00058 <=> C00131 + C00011 + C00001	ATP + Formate	dATP + CO2 + H2O
R03194	35	2C00019-SYN+C01051<=>2C00021-SYN+C02463	2S-Adenosyl-L-methionine+UroporphyrinogenIII	2S-Adenosyl-L-homocysteine+Precorrin2
R05808	35	C11538 + C00019-SYN <=> C17401 + C00021-SYN	Cobalt-sirohydrochlorin + S-Adenosyl-L-methionine	Cobalt-factorIII + S-Adenosyl-L-homocysteine
R07773	35	C16243 + C00019-SYN <=> C11542 + C00021-SYN	Cobalt-precorrin 5B + S-Adenosyl-L-methionine	Cobalt-precorrin 6 + S-Adenosyl-L-homocysteine
R07774	35	C11543 + C00019-SYN <=> C16244 + C00021-SYN + C00011	Cobalt-dihydro-precorrin 6 + S-Adenosyl-L-methionine	Cobalt-precorrin 7 + S-Adenosyl-L-homocysteine + CO2
R09543	35	C00019-SYN + C01209 <=> C00021-SYN + C19673	S-Adenosyl-L-methionine + Malonyl-[acyl-carrier protein]	S-Adenosyl-L-homocysteine + Malonyl-[acp] methyl ester
R05810	35	C11540 + C00019-SYN <=> C16242 + C00021-SYN	Cobalt-precorrin 4 + S-Adenosyl-L-methionine	Cobalt-precorrin 5A + S-Adenosyl-L-homocysteine
R11580	35	C00019-SYN + C17401 + C00030 <=> C00021-SYN + C11540 + C00028	S-Adenosyl-L-methionine + Cobalt-factor III + Reducedacceptor	S-Adenosyl-L-homocysteine + Cobalt-precorrin 4 + Acceptor
R03231	35	C00019-SYN + C01092 <=> C04425 + C01037	S-Adenosyl-L-methionine + 8-Amino-7-oxononanoate	S-Adenosyl-4-methylthio-2-oxobutanoate + 7,8-Diaminononanoate
R00130	35	C00002 + C00882 <=> C00008 + C00010-SYN	ATP + Dephospho-CoA	ADP + CoA
R07461	35	C15812 + C15813 + C00030 <=> C00020-SYN + C15814 + C15811 + C00028	[Enzyme]-S-sulfanylcysteine + Adenylyl-[sulfur-carrierprotein] + Reduced acceptor	AMP + Thiocarboxy-[sulfur-carrierprotein] + [Enzyme]-cysteine + Acceptor
R01078	35	C01909 + C17023 + 2 C00019-SYN + 2 C05359 + 2 C00080 <=> C00120 + 2 C00073 + 2 C05198	Dethiobiotin + Sulfurdonor + 2 S-Adenosyl-L-methionine + 2 e- + 2 H+	Biotin + 2 L-Methionine + 2 5'-Deoxyadenosine
R05046	35	C05922 + C00001 <=> C05923 + C00058	Formamidopyrimidine nucleoside triphosphate + H2O	2,5-Diaminopyrimidine nucleoside triphosphate + Formate
R03459	35	C01304 + C00001 <=> C01268 + C00014	2,5-Diamino-6-(5-phospho-D-ribosylamino)pyrimidin-4(3H)-one + H2O	5-Amino-6-(5'-phosphoribosylamino)uracil + Ammonia
R04035	35	C02739 + C00001 <=> C02741 + C00013	1-(5-Phospho-D-ribosyl)-ATP + H2O	Phosphoribosyl-AMP + Diphosphate
R03472	35	C03373 + C00019-SYN <=> C04556 + C05198 + C00073 + C00058 + C00237	Aminoimidazole ribotide + S-Adenosyl-L-methionine	4-Amino-2-methyl-5-(phosphooxymethyl)pyrimidine + 5'-Deoxyadenosine + L-Methionine + Formate + CO
R10246	35	C00082 + C00019-SYN + C00030 <=> C15809 + C01468 + C05198 + C00073 + C00028	L-Tyrosine + S-Adenosyl-L-methionine + Reduced acceptor	Iminoglycine + 4-Cresol + 5'-Deoxyadenosine + L-Methionine + Acceptor
R09394	35	C00044-SYN + C00019-SYN + C00030 <=> C21310 + C05198 + C00073 + C00028	GTP + S-Adenosyl-L-methionine + Reduced acceptor	(8S)-3',8-Cyclo-7,8-dihydroguanosine 5'-triphosphate + 5'-Deoxyadenosine + L-Methionine + Acceptor
R00189	35	C00002 + C00857 + C00014 <=> C00020 + C00013 + C00003-SYN	ATP + Deamino-NAD+ + Ammonia	AMP + Diphosphate + NAD+
R00257	35	C00002 + C00857 + C00064 + C00001 <=> C00020 + C00013 + C00003-SYN + C00025	ATP + Deamino-NAD+ + L-Glutamine + H2O	AMP + Diphosphate + NAD+ + L-Glutamate
R05225	35	C06506 + 4 C00064 + 4 C00002 + 4 C00001 <=> C06507 + 4 C00025 + 4 C00009 + 4 C00008	Adenosyl cobyrinatea,cdiamide + 4L-Glutamine + 4ATP + 4H2O	Adenosyl cobyrinate hexaamide + 4L-Glutamate + 4 Orthophosphate + 4 ADP
R05809	35	C11539 + C00019-SYN <=> C11540 + C00021-SYN	Cobalt-precorrin 3 + S-Adenosyl-L-methionine	Cobalt-precorrin 4 + S-Adenosyl-L-homocysteine
R07775	35	C16244 + C00019-SYN <=> C11545 + C00021-SYN	Cobalt-precorrin 7 + S-Adenosyl-L-methionine	Cobalt-precorrin 8 + S-Adenosyl-L-homocysteine
R11072	35	C01304 <=> C05925 + C00001	2,5-Diamino-6-(5-phospho-D-ribosylamino)pyrimidin-4(3H)-one	Dihydroneopterin phosphate + H2O
R11919	35	C03373 + C00019-SYN + C00030 <=> C21764 + C05198 + C00073 + C00058 + C00014 + C00009 + C00028	Aminoimidazole ribotide + S-Adenosyl-L-methionine + Reduced acceptor	5-Hydroxybenzimidazole + 5'-Deoxyadenosine + L-Methionine + Formate + Ammonia + Orthophosphate + Acceptor
R12026	36	C00003-SYN + C00037 + C00283 <=> C00153 + C20784 + 3 C00001	NAD+ + Glycine + Hydrogen sulfide	Nicotinamide + ADP-5-ethyl-4-methylthiazole-2-carboxylate + 3 H2O
R00104	36	C00002 + C00003-SYN <=> C00008 + C00006-SYN	ATP + NAD+	ADP + NADP+
R04509	36	C00002 + C04556 <=> C00008 + C04752	ATP + 4-Amino-2-methyl-5-(phosphooxymethyl)pyrimidine	ADP + 4-Amino-5-hydroxymethyl-2-methylpyrimidine diphosphate
R10247	36	C11437 + C15809 + C15814 <=> C20246 + C15810 + 2 C00001	1-Deoxy-D-xylulose 5-phosphate + Iminoglycine + Thiocarboxy-[sulfur-carrierprotein]	2-[(2R,5Z)-2-Carboxy-4-methylthiazol-5(2H)-ylidene]ethyl phosphate + Sulfur-carrierprotein + 2 H2O
R00194	36	C00021-SYN + C00001 <=> C03539 + C00147	S-Adenosyl-L-homocysteine + H2O	S-Ribosyl-L-homocysteine + Adenine
R05048	36	C05923 <=> C06148	2,5-Diaminopyrimidine nucleoside triphosphate	2,5-Diamino-6-(5'-triphosphoryl-3',4'-trihydroxy-2'-oxopentyl)-amino-4-oxopyrimidine
R04037	36	C02741 + C00001 <=> C04896	Phosphoribosyl-AMP + H2O	5-(5-Phospho-D-ribosylaminoformimino)-1-(5-phosphoribosyl)-imidazole-4-carboxamide
R11372	36	C21310 + C00001 <=> C18239 + C00013	(8S)-3',8-Cyclo-7,8-dihydroguanosine 5'-triphosphate + H2O	PrecursorZ + Diphosphate
R07302	36	C00002 + C06507 + C03194 <=> C00008 + C00009 + C06508	ATP + Adenosyl cobyrinate hexaamide + (R)-1-Aminopropan-2-ol	ADP + Orthophosphate + Adenosyl cobinamide

R04621	36	C05925 + C00001 <=>C04874 + C00009	Dihydroneopterin phosphate +H2O	7,8-Dihydroneopterin +Orthophosphate
R12854	36	C00019-SYN + C21764 <=>C00021-SYN + C22450	S-Adenosyl-L-methionine +5-Hydroxybenzimidazole	S-Adenosyl-L-homocysteine + 5-Methoxybenzimidazole
R03458	36	C01268 + C00005+ C00080 <=>C04454 + C00006	5-Amino-6-(5'-phosphoribosylamino)uracil + NADPH+ H+	5-Amino-6-(5'-phospho-D-ribitylamino)uracil + NADP+
R09395	37	C18239 + 2 C15814 <=>C05924 + 2 C15810	PrecursorZ + 2 Thiocarboxy-[sulfur-carrierprotein]	Molybdopterin + 2 Sulfur-carrier protein
R07280	37	C04454 + C00001 <=>C04732 + C00009	5-Amino-6-(5'-phospho-D-ribitylamino)uracil +H2O	5-Amino-6-(1-D-ribitylamino)uracil +Orthophosphate
R04639	37	C06148 <=>C04895 + C00001	2,5-Diamino-6-(5'-triphosphoryl-3',4'-trihydroxy-2'-oxopentyl)-amino-4-oxopyrimidine	7,8-Dihydroneopterin 3'-triphosphate +H2O
R03504	37	C04874 <=>C00266 + C01300	7,8-Dihydroneopterin	Glycolaldehyde +6-(Hydroxymethyl)-7,8-dihydropterin
R01291	37	C03539 <=>C11838 + C00155	S-Ribosyl-L-homocysteine	(S)-4,5-Dihydroxypentane-2,3-dione +L-Homocysteine
R04640	37	C04896 <=> C04916	5-(5-Phospho-D-ribosylaminoformimino)-1-(5-phosphoribosyl)-imidazole-4-carboxamide	N-(5'-Phospho-D-1'-ribulosylformimino)-5-amino-1-(5''-phospho-D-ribosyl)-4-imidazolecarboxamide
R05221	37	C06508 + C00002 <=>C06509 + C00008	Adenosyl cobinamide+ ATP	Adenosyl cobinamide phosphate + ADP
R06558	37	C06508 + C00044 <=>C06509 + C00035	Adenosyl cobinamide +GTP	Adenosyl cobinamide phosphate +GDP
R12855	37	C22450 <=> C22451	5-Methoxybenzimidazole	5-Methoxy-6-methylbenzimidazole
R09977	37	C20246 <=> C20247	2-[(2R,5Z)-2-Carboxy-4-methylthiazol-5(2H)-ylidene]ethyl phosphate	2-(2-Carboxy-4-methylthiazol-5-ylEthyl phosphate
R12161	38	C04732 + C00082+ C00019-SYN <=>C21971 + C15809+ C00073 +C05198	5-Amino-6-(1-D-ribitylamino)uracil +L-Tyrosine + S-Adenosyl-L-methionine	5-Amino-5-(4-hydroxybenzyl)-6-(D-ribitylimino)-5,6-dihydrouracil + Iminoglycine +L-Methionine+ 5'-Deoxyadenosine
R03066	38	C01300 + C00568 <=>C00921 + C00001	6-(Hydroxymethyl)-7,8-dihydropterin + 4-Aminobenzoate	Dihydropteroate + H2O
R10712	38	C04752 + C20247<=> C01081+ C00013 +C00011	4-Amino-5-hydroxymethyl-2-methylpyrimidine diphosphate+ 2-(2-Carboxy-4-methylthiazol-5-yl)ethyl phosphate	Thiamin monophosphate+ Diphosphate +CO2
R04457	38	C04732 + C15556<=> C04332+ 2 C00001+ C00009	5-Amino-6-(1-D-ribitylamino)uracil+ L-3,4-Dihydroxybutan-2-one 4-phosphate	6,7-Dimethyl-8-(D-ribityl)lumazine +2 H2O + Orthophosphate
R03503	38	C00002 + C01300 <=>C00020 + C04807	ATP + 6-(Hydroxymethyl)-7,8-dihydropterin	AMP +6-Hydroxymethyl-7,8-dihydropterin diphosphate
R05222	38	C06509 + C00044-SYN <=>C06510 + C00013	Adenosyl cobinamide phosphate +GTP	Adenosine-GDP-cobinamide + Diphosphate
R09726	38	C00002-SYN + C05924 <=>C00013 + C19848	ATP + Molybdopterin	Diphosphate + Adenylated molybdopterin
R04620	38	C04895 + 3 C00001 <=>C04874 + 3 C00009	7,8-Dihydroneopterin 3'-triphosphate +3 H2O	7,8-Dihydroneopterin +3 Orthophosphate
R04638	38	C04895 + C00001 <=>C05925 + C00013	7,8-Dihydroneopterin 3'-triphosphate +H2O	Dihydroneopterin phosphate +Diphosphate
R04558	38	C04916 + C00064<=> C04666+ C04677 +C00025	N-(5'-Phospho-D-1'-ribulosylformimino)-5-amino-1-(5''-phospho-D-ribosyl)-4-imidazolecarboxamide + L-Glutamine	D-erythro-1-(Imidazol-4-yl)glycerol 3-phosphate + 1-(5'-Phosphoribosyl)-5-amino-4-imidazolecarboxamide+ L-Glutamate
R12856	38	C22451 <=>C03114	5-Methoxy-6-methylbenzimidazole	Dimethylbenzimidazole
R11719	38	C04895 <=>C01300 + C21615	7,8-Dihydroneopterin 3'-triphosphate	6-(Hydroxymethyl)-7,8-dihydropterin + Glycolaldehyde triphosphate
R09735	39	C19848 + C06232 <=>C18237 + C00020-SYN+ C00001	Adenylated molybdopterin + Molybdate	Molybdoenzyme molybdenum cofactor + AMP+ H2O
R04148	39	C01185 + C03114 <=>C00253 + C04778+ C00080	Nicotinate D-ribonucleotide + Dimethylbenzimidazole	Nicotinate +N1-(5-Phospho-alpha-D-ribosyl)-5,6-dimethylbenzimidazole + H+
R03067	39	C04807 + C00568 <=>C00013 + C00921	6-Hydroxymethyl-7,8-dihydropterin diphosphate + 4-Aminobenzoate	Diphosphate +Dihydropteroate
R00066	39	2C04332<=>C00255+C04732	26,7-Dimethyl-8-(D-ribityl)lumazine	Riboflavin +5-Amino-6-(1-D-ribitylamino)uracil
R00617	39	C00002 + C01081 <=>C00008 + C00068	ATP + Thiamin monophosphate	ADP + Thiamin diphosphate
R03457	39	C04666 <=>C01267 + C00001	D-erythro-1-(Imidazol-4-yl)glycerol 3-phosphate	3-(Imidazol-4-yl)-2-oxopropyl phosphate +H2O
R12162	39	C21971 + C00019-SYN <=>C19154 + C00014+ C00073 +C05198	5-Amino-5-(4-hydroxybenzyl)-6-(D-ribitylimino)-5,6-dihydrouracil + S-Adenosyl-L-methionine	7,8-Didemethyl-8-hydroxy-5-deazariboflavin + Ammonia +L-Methionine+ 5'-Deoxyadenosine
R02237	39	C00002 + C00921+ C00025 <=>C00008 + C00009+ C00415	ATP + Dihydropteroate +L-Glutamate	ADP +Orthophosphate + Dihydrofolate
R10339	39	C04807 + C20559 <=>C20562 + C00013	6-Hydroxymethyl-7,8-dihydropterin diphosphate + 4-(beta-D-Ribofuranosyl)aniline5'-phosphate	N-[(7,8-Dihydropterin-6-yl)methyl]-4-(beta-D-ribofuranosyl)aniline 5'-phosphate + Diphosphate
R03243	40	C01267 + C00025<=> C01100 +C00026	3-(Imidazol-4-yl)-2-oxopropyl phosphate +L-Glutamate	L-Histidinol phosphate+ 2-Oxoglutarate
R00549	40	C00002 + C00255 <=>C00008 + C00061-SYN	ATP + Riboflavin	ADP +FMN
R04594	40	C04778 + C00001 <=>C05775 + C00009	N1-(5-Phospho-alpha-D-ribosyl)-5,6-dimethylbenzimidazole + H2O	alpha-Ribazole +Orthophosphate
RMAN11	40	C19154 + C22297 <=>C22277 + C00144-SYN	7,8-Didemethyl-8-hydroxy-5-deazariboflavin + Enolpyruvoyl-2-diphospho-5'-guanosine	Dehydro coenzyme F420-0+ GMP
RMAN2	40	C20562 <=> C05927	N-[(7,8-Dihydropterin-6-yl)methyl]-4-(beta-D-ribofuranosyl)aniline 5'-phosphate	7,8-Dihydromethanopterin
R10802	41	C05927 + C00030 <=>C01217-SYN + C00028	7,8-Dihydromethanopterin +Reduced acceptor	5,6,7,8-Tetrahydromethanopterin + Acceptor
R00161	41	C00002-SYN + C00061-SYN <=>C00013 + C00016	ATP +FMN	Diphosphate +FAD
R05223	41	C06510 + C05775 <=>C00194 + C00144-SYN	Adenosine-GDP-cobinamide + alpha-Ribazole	Cobamide coenzyme + GMP
R03013	41	C01100 + C00001 <=>C00860 + C00009	L-Histidinol phosphate +H2O	L-Histidinol +Orthophosphat
RMAN12	41	C22277 + C01847<=> C19153+ C00061 +C00080	Dehydro coenzyme F420-0+ Reduced FMN	Coenzyme F420-0 +FMN + H+
R03388	41	C05927 + C00080 +C00005 <=>C01217-SYN + C00006	7,8-Dihydromethanopterin + H++ NADPH	5,6,7,8-Tetrahydromethanopterin + NADP+
R03012	42	C00860 + C00003<=> C01929+ C00004 +C00080	L-Histidinol +NAD+	L-Histidinal +NADH + H+
R09399	42	C19153 + C00044+ C00025 <=>C19152 + C00035+ C00009	Coenzyme F420-0 +GTP + L-Glutamate	Coenzyme F420-1 +GDP + Orthophosphate
R01163	43	C01929 + C00001+ C00003 <=>C00135 + C00004+ C00080	L-Histidinal +H2O + NAD+	L-Histidine + NADH +H+
R09400	43	C19152 + C00044+ C00025 <=>C00876-SYN + C00035 +C00009	Coenzyme F420-1 +GTP + L-Glutamate	Coenzyme F420 +GDP + Orthophosphate

Table S2. Overview of phosphate-related reactions (Figure 2A), including Reaction ID, stratification rank, recoded reaction equation, reaction equations in compound names (left and right), pathway information, and xDP/xTP involvement.

Reaction ID	Rank	Recoded reaction equation	Reaction equation (Compound name) left	Reaction equation (Compound name) right	Pathway	xDP/xTP
R00149	1	2 C00002 + C00014 + C00011 + C00001 <=> 2 C00008 + C00009 + C00169	2 ATP + Ammonia + CO2 + H2O	2 ADP + Orthophosphate + Carbamoyl phosphate	Arginine	1
R00943	2	C00101 + C00058 + C00002 <=> C00008 + C00009 + C00234	Tetrahydrofolate + Formate + ATP	ADP + Orthophosphate + 10-Formyltetrahydrofolate	Carbon fixation, WL	1
R00230	7	C00024 + C00009 <=> C00010 + C00227	Acetyl-CoA + Orthophosphate	CoA + Acetyl phosphate	Carbon fixation, WL	
R00742	7	C00002 + C00024 + C00288 <=> C00008 + C00009 + C00083	ATP + Acetyl-CoA + HCO3-	ADP + Orthophosphate + Malonyl-CoA	Carbon fixation	1
R00199	8	C00002 + C00022 + C00001 <=> C00020 + C00074 + C00009	ATP + Pyruvate + H2O	AMP + Phosphoenolpyruvate + Orthophosphate	Carbon fixation, rTCA	1
R00344	8	C00002 + C00022 + C00288 <=> C00008 + C00009 + C00036	ATP + Pyruvate + HCO3-	ADP + Orthophosphate + Oxaloacetate	Carbon fixation, rTCA	1
R00206	8	C00002 + C00022 + C00009 <=> C00020 + C00074 + C00013	ATP + Pyruvate + Orthophosphate	AMP + Phosphoenolpyruvate + Diphosphate	Carbon fixation, rTCA	1
R00341	9	C00002 + C00036 <=> C00008 + C00074 + C00011	ATP + Oxaloacetate	ADP + Phosphoenolpyruvate + CO2	Glycolysis/gluconeogenesis	1
R00431	9	C00044 + C00036 <=> C00035 + C00074 + C00011	GTP + Oxaloacetate	GDP + Phosphoenolpyruvate + CO2	Glycolysis/gluconeogenesis	1
R00345	9	C00001 + C00074 + C00011 <=> C00009 + C00036	H2O + Phosphoenolpyruvate + CO2	Orthophosphate + Oxaloacetate	Carbon fixation, rTCA	
R05789	10	C11536 + C00001 <=> C11537 + C00009	(2R)-O-Phospho-3-sulfolactate + H2O	(2R)-3-Sulfolactate + Orthophosphate	CoM	
R01518	10	C00631 <=> C00197	2-Phospho-D-glycerate	3-Phospho-D-glycerate	Glycolysis/gluconeogenesis	
R01512	11	C00002 + C00197 <=> C00008 + C00236	ATP + 3-Phospho-D-glycerate	ADP + 3-Phospho-D-glyceroyl phosphate	Glycolysis/gluconeogenesis	1
R01061	12	C00236 + C00004 + C00080 <=> C00118 + C00009 + C00003	3-Phospho-D-glyceroyl phosphate + NADH + H+	D-Glyceraldehyde 3-phosphate + Orthophosphate + NAD+	Glycolysis/gluconeogenesis	
R01063	12	C00236 + C00005 + C00080 <=> C00118 + C00009 + C00006	3-Phospho-D-glyceroyl phosphate + NADPH + H+	D-Glyceraldehyde 3-phosphate + Orthophosphate + NADP+	Glycolysis/gluconeogenesis	
R00405	12	C00002 + C00042 + C00010 <=> C00008 + C00009 + C00091	ATP + Succinate + CoA	ADP + Orthophosphate + Succinyl-CoA	Carbon fixation, rTCA	1
R10935	13	2 C00118 <=> C20954 + C00009 + 2 C00001	2 D-Glyceraldehyde 3-phosphate	(5-Formylfuran-3-yl)methyl phosphate + Orthophosphate + 2 H2O	Methanofuran	
R11039	15	C21068 + C00002 <=> C21069 + C00008	[5-(Aminomethyl)furan-3-yl]methyl phosphate + ATP	[5-(Aminomethyl)furan-3-yl]methyl diphosphate + ADP	Methanofuran	1
R00239	15	C00002 + C00025 <=> C00008 + C03287	ATP + L-Glutamate	ADP + L-Glutamyl 5-phosphate	Proline	1
R00253	15	C00002 + C00025 + C00014 <=> C00008 + C00009 + C00064	ATP + L-Glutamate + Ammonia	ADP + Orthophosphate + L-Glutamine	Glutamine	1
R04780	15	C05378 + C00001 <=> C05345 + C00009	beta-D-Fructose 1,6-bisphosphate + H2O	beta-D-Fructose 6-phosphate + Orthophosphate	Glycolysis/gluconeogenesis	
R02073	15	C00009 + C05378 <=> C00013 + C05345	Orthophosphate + beta-D-Fructose 1,6-bisphosphate	Diphosphate + beta-D-Fructose 6-phosphate	Glycolysis/gluconeogenesis	
R00575	16	2 C00002 + C00064 + C00288 + C00001 <=> 2 C00008 + C00009 + C00025 + C00169	2 ATP + L-Glutamine + HCO3- + H2O	2 ADP + Orthophosphate + L-Glutamate + Carbamoyl phosphate	Pyrimidine	1
R04779	16	C00002 + C05345 <=> C00008 + C05378	ATP + beta-D-Fructose 6-phosphate	ADP + beta-D-Fructose 1,6-bisphosphate	Glycolysis/gluconeogenesis	1
R00480	16	C00002 + C00049 <=> C00008 + C03082	ATP + L-Aspartate	ADP + 4-Phospho-L-aspartate	Lysine,Threonine,Methionine	1
R00482	16	C00002 + C00049 + C00014 <=> C00008 + C00009 + C00152	ATP + L-Aspartate + Ammonia	ADP + Orthophosphate + L-Asparagine	Asparagine	1
R00483	16	C00002 + C00049 + C00014 <=> C00020 + C00013 + C00152	ATP + L-Aspartate + Ammonia	AMP + Diphosphate + L-Asparagine	Asparagine	1
R00578	16	C00002 + C00049 + C00064 + C00001 <=> C00020 + C00013 + C00152 + C00025	ATP + L-Aspartate + L-Glutamine + H2O	AMP + Diphosphate + L-Asparagine + L-Glutamate	Asparagine	1
R02649	16	C00002 + C00624 <=> C00008 + C04133	ATP + N-Acetyl-L-glutamate	ADP + N-Acetyl-L-glutamate 5-phosphate	Arginine	1
R09084	16	C05345 + C00008 <=> C05378 + C00020	beta-D-Fructose 6-phosphate + ADP	beta-D-Fructose 1,6-bisphosphate + AMP	Glycolysis/gluconeogenesis	1
R01397	16	C00169 + C00049 <=> C00009 + C00438	Carbamoyl phosphate + L-Aspartate	Orthophosphate + N-Carbamoyl-L-aspartate	Pyrimidine	
R03313	16	C03287 + C00005 + C00080 <=> C01165 + C00009 + C00006	L-Glutamyl 5-phosphate + NADPH + H+	L-Glutamate 5-semialdehyde + Orthophosphate + NADP+	Proline	
R00582	16	C01005 + C00001 <=> C00065 + C00009	O-Phospho-L-serine + H2O	L-Serine + Orthophosphate	Serine	
R07274	16	C01005 + C00283 <=> C00097 + C00009	O-Phospho-L-serine + Hydrogen sulfide	L-Cysteine + Orthophosphate	Cysteine	
R00764	16	C00009 + C00354 <=> C00013 + C00085	Orthophosphate + D-Fructose 1,6-bisphosphate	Diphosphate + D-Fructose 6-phosphate	Glycolysis/gluconeogenesis	
R02291	17	C03082 + C00005 + C00080 <=> C00441 + C00009 + C00006	4-Phospho-L-aspartate + NADPH + H+	L-Aspartate 4-semialdehyde + Orthophosphate + NADP+	Lysine,Threonine,Methionine	
R05805	17	C00008 + C00085 <=> C00020 + C00354	ADP + D-Fructose 6-phosphate	AMP + D-Fructose 1,6-bisphosphate	Glycolysis/gluconeogenesis	1
R12342	17	C00008 + C00065 <=> C00020 + C01005	ADP + L-Serine	AMP + O-Phospho-L-serine	Cysteine	1
R00959	17	C00668 <=> C00103	alpha-D-Glucose 6-phosphate	D-Glucose 1-phosphate	Glycolysis/gluconeogenesis	
R01788	17	C00668 + C00001 <=> C00267 + C00009	alpha-D-Glucose 6-phosphate + H2O	alpha-D-Glucose + Orthophosphate	Glycolysis/gluconeogenesis	
R02473	17	C00002 + C05222 + C00099 <=> C00020 + C00013 + C00864	ATP + (R)-Pantoate + beta-Alanine	AMP + Diphosphate + Pantothenate	CoA	1
R00352	17	C00002 + C00158 + C00010 <=> C00008 + C00009 + C00024 + C00036	ATP + Citrate + CoA	ADP + Orthophosphate + Acetyl-CoA + Oxaloacetate	Carbon fixation, rTCA	1
R01322	17	C00002 + C00158 + C00010 <=> C00008 + C00009 + C00566	ATP + Citrate + CoA	ADP + Orthophosphate + (3S)-Citryl-CoA	Carbon fixation, rTCA	1
R00756	17	C00002 + C00085 <=> C00008 + C00354	ATP + D-Fructose 6-phosphate	ADP + D-Fructose 1,6-bisphosphate	Glycolysis/gluconeogenesis	1
R04292	17	C05840 + C00111 <=> C03722 + 2 C00001 + C00009	Iminoaspartate + Glycerone phosphate	Quinolinate + 2 H2O + Orthophosphate	NAD	
R03443	17	C04133 + C00005 + C00080 <=> C01250 + C00009 + C00006	N-Acetyl-L-glutamate 5-phosphate + NADPH + H+	N-Acetyl-L-glutamate 5-semialdehyde + Orthophosphate + NADP+	Arginine	
R09085	18	C00267 + C00008 <=> C00668 + C00020	alpha-D-Glucose + ADP	alpha-D-Glucose 6-phosphate + AMP	Glycolysis/gluconeogenesis	1
R01786	18	C00002 + C00267 <=> C00008 + C00668	ATP + alpha-D-Glucose	ADP + alpha-D-Glucose 6-phosphate	Glycolysis/gluconeogenesis	1
R03018	18	C00002 + C00864 <=> C00008 + C03492	ATP + Pantothenate	ADP + D-4'-Phosphopantothenate	CoA	1
R00947	18	C00103 + C00001 <=> C00267 + C00009	D-Glucose 1-phosphate + H2O	alpha-D-Glucose + Orthophosphate	Glycolysis/gluconeogenesis	
R05804	19	C00008 + C00031 <=> C00020 + C00092	ADP + D-Glucose	AMP + D-Glucose 6-phosphate	Glycolysis/gluconeogenesis	1
R01600	19	C00002 + C00221 <=> C00008 + C01172	ATP + beta-D-Glucose	ADP + beta-D-Glucose 6-phosphate	Glycolysis/gluconeogenesis	1
R04230	19	C00002 + C03492 + C00097 <=> C00020 + C00013 + C04352	ATP + D-4'-Phosphopantothenate + L-Cysteine	AMP + Diphosphate + (R)-4'-Phosphopantothenoyl-L-cysteine	CoA	1
R00299	19	C00002 + C00031 <=> C00008 + C00092	ATP + D-Glucose	ADP + D-Glucose 6-phosphate	Glycolysis/gluconeogenesis	1
R01049	19	C00002 + C00117 <=> C00020 + C00119	ATP + D-Ribose 5-phosphate	AMP + 5-Phospho-alpha-D-ribose 1-diphosphate	Pentose phosphate pathway	1
R01771	19	C00002 + C00263 <=> C00008 + C01102	ATP + L-Homoserine	ADP + O-Phospho-L-homoserine	Threonine	1
R09086	19	C00221 + C00008 <=> C01172 + C00020	beta-D-Glucose + ADP	beta-D-Glucose 6-phosphate + AMP	Glycolysis/gluconeogenesis	1
R07245	19	C00169 + C00437 <=> C00009 + C15532	Carbamoyl phosphate + N-Acetylornithine	Orthophosphate + N-Acetyl-L-citrulline	Arginine	
R04231	19	C00063 + C03492 + C00097 <=> C00055 + C00013 + C04352	CTP + D-4'-Phosphopantothenate + L-Cysteine	CMP + Diphosphate + (R)-4'-Phosphopantothenoyl-L-cysteine	CoA	1
R10089	19	C00117 + C00118 + C00064 <=> C00018 + C00025 + 3 C00001 + C00009	D-Ribose 5-phosphate + D-Glyceraldehyde 3-phosphate + L-Glutamine	Pyridoxal phosphate + L-Glutamate + 3 H2O + Orthophosphate	PLP	
R01398	20	C00169 + C00077 <=> C00009 + C00327	Carbamoyl phosphate + L-Ornithine	Orthophosphate + L-Citrulline	Arginine	
R01072	20	C00064 + C00119 + C00001 <=> C03090 + C00013 + C00025	L-Glutamine + 5-Phospho-alpha-D-ribose 1-diphosphate + H2O	5-Phosphoribosylamine + Diphosphate + L-Glutamate	Purine	
R01466	20	C01102 + C00001 <=> C00188 + C00009	O-Phospho-L-homoserine + H2O	L-Threonine + Orthophosphate	Threonine	
R01870	20	C00295 + C00119 <=> C01103 + C00013	Orotate + 5-Phospho-alpha-D-ribose 1-diphosphate	Orotidine 5'-phosphate + Diphosphate	Pyrimidine	
R03348	20	C03722 + C00119 <=> C01185 + C00013 + C00011	Quinolinate + 5-Phospho-alpha-D-ribose 1-diphosphate	Nicotinate D-ribonucleotide + Diphosphate + CO2	NAD	
R04144	21	C00002 + C03090 + C00037 <=> C00008 + C00009 + C03838	ATP + 5-Phosphoribosylamine + Glycine	ADP + Orthophosphate + 5'-Phosphoribosylglycinamide	Purine	1
R01954	21	C00002 + C00327 + C00049 <=> C00020 + C00013 + C03406	ATP + L-Citrulline + L-Aspartate	AMP + Diphosphate + N-(L-Arginino)succinate	Arginine	1
R01826	21	C00074 + C00279 + C00001 <=> C04691 + C00009	Phosphoenolpyruvate + D-Erythrose 4-phosphate + H2O	2-Dehydro-3-deoxy-D-arabino-heptonate 7-phosphate + Orthophosphate	Tryptothan,Phenylalanine,Tyrosine	
R03083	22	C04691 <=> C00944 + C00009	2-Dehydro-3-deoxy-D-arabino-heptonate 7-phosphate	3-Dehydroquinate + Orthophosphate	Tryptothan,Phenylalanine,Tyrosine	
R00158	22	C00002 + C00105 <=> C00008 + C00015	ATP + UMP	ADP + UDP	Pyrimidine	1
R04463	23	C00002 + C04376 + C00064 + C00001 <=> C00008 + C00009 + C04640 + C00025	ATP + 5'-Phosphoribosyl-N-formylglycinamide + L-Glutamine + H2O	ADP + Orthophosphate + 2-(Formamido)-N1-(5'-phosphoribosyl)acetamidine + L-Glutamate	Purine	1
R00156	23	C00002 + C00015 <=> C00008 + C00075	ATP + UDP	ADP + UTP	Pyrimidine	1
R11627	23	C21510 + 2 C00002 + 2 C00064 + 2 C00001 <=> C21511 + 2 C00008 + 2 C00025 + 2 C00009	Nickel-sirohydrochlorin + 2 ATP + 2 L-Glutamine + 2 H2O	Nickel-sirohydrochlorin a,c-diamide + 2 ADP + 2 L-Glutamate + 2 Orthophosphate	F430	1
R04208	24	C00002 + C04640 <=> C00008 + C00009 + C03373	ATP + 2-(Formamido)-N1-(5'-phosphoribosyl)acetamidine	ADP + Orthophosphate + Aminoimidazole ribotide	Purine	1
R03182	24	C00002 + C01037 + C00011 <=> C00008 + C00009 + C01909	ATP + 7,8-Diaminononanoate + CO2	ADP + Orthophosphate + Dethiobiotin	Biotin	1
R11628	24	C00002 + C21511 + 3 C00030 + C00001 <=> C00008 + C00009 + C21512 + 3 C00028	ATP + Nickel-sirohydrochlorin a,c-diamide + 3 Reduced acceptor + H2O	ADP + Orthophosphate + 15,17(3)-Seco-F430-17(3)-acid + 3 Acceptor	F430	1
R00571	24	C00002 + C00075 + C00014 <=> C00008 + C00009 + C00063-SYN	ATP + UTP + Ammonia	ADP + Orthophosphate + CTP	Pyrimidine	1
R00573	24	C00002 + C00075 + C00064 + C00001 <=> C00008 + C00009 + C00063-SYN + C00025	ATP + UTP + L-Glutamine + H2O	ADP + Orthophosphate + CTP + L-Glutamate	Pyrimidine	1
R11629	25	C21512 + C00002 <=> C05777 + C00008 + C00009	15,17(3)-Seco-F430-17(3)-acid + ATP	Coenzyme F430 + ADP + Orthophosphate	F430	1
R07404	25	C00002 + C03373 + C00288 <=> C00008 + C00009 + C15667	ATP + Aminoimidazole ribotide + HCO3-	ADP + Orthophosphate + 5-Carboxyamino-1-(5-phospho-D-ribosyl)imidazole	Purine	1
R02412	25	C00002 + C00493 <=> C00008 + C03175	ATP + Shikimate	ADP + Shikimate 3-phosphate	Tryptothan,Phenylalanine,Tyrosine	1
R04591	26	C00002 + C04751 + C00049 <=> C00008 + C00009 + C04823	ATP + 1-(5-Phospho-D-ribosyl)-5-amino-4-imidazolecarboxylate + L-Aspartate	ADP + Orthophosphate + 1-(5'-Phosphoribosyl)-5-amino-4-(N-succinocarboxamide)-imidazole	Purine	1
R03460	26	C00074 + C03175 <=> C00009 + C01269	Phosphoenolpyruvate + Shikimate 3-phosphate	Orthophosphate + 5-O-(1-Carboxyvinyl)-3-phosphoshikimate	Tryptothan,Phenylalanine,Tyrosine	
R01714	27	C01269 <=> C00251 + C00009	5-O-(1-Carboxyvinyl)-3-phosphoshikimate	Chorismate + Orthophosphate	Tryptothan,Phenylalanine,Tyrosine	
R02100	27	C00460 + C00001 <=> C00365 + C00013	dUTP + H2O	dUMP + Diphosphate	Pyrimidine	1
R08323	28	C16593 + C00188 + C00002 <=> C16594 + C00008 + C00009	7-Mercaptoheptanoic acid + L-Threonine + ATP	7-Mercaptoheptanoylthreonine + ADP + Orthophosphate	CoB	1
R06975	28	C00058 + C00002 + C04677 <=> C00008 + C00009 + C04734	Formate + ATP + 1-(5'-Phosphoribosyl)-5-amino-4-imidazolecarboxamide	ADP + Orthophosphate + 1-(5'-Phosphoribosyl)-5-formamido-4-imidazolecarboxamide	Purine	1
R11102	29	C00119 + C00156 <=> C21107 + C00011 + C00013	5-Phospho-alpha-D-ribose 1-diphosphate + 4-Hydroxybenzoate	4-(beta-D-Ribofuranosyl)phenol 5'-phosphate + CO2 + Diphosphate	THMPT	
R08217	29	C16594 + C00002 <=> C04628 + C00008	7-Mercaptoheptanoylthreonine + ATP	Coenzyme B + ADP	CoB	1
R01073	29	C00108 + C00119 <=> C04302 + C00013	Anthranilate + 5-Phospho-alpha-D-ribose 1-diphosphate	N-(5-Phospho-D-ribosyl)anthranilate + Diphosphate	Tryptophan	
R02094	29	C00002 + C00364 <=> C00008 + C00363	ATP + dTMP	ADP + dTDP	Pyrimidine	1
R02093	30	C00002 + C00363 <=> C00008 + C00459	ATP + dTDP	ADP + dTTP	Pyrimidine	1
R01135	30	C00044 + C00130 + C00049 <=> C00035 + C00009 + C03794	GTP + IMP + L-Aspartate	GDP + Orthophosphate + N6-(1,2-Dicarboxyethyl)-AMP	Purine,ATP	1
R01230	31	C00002 + C00655 + C00014 <=> C00020 + C00013 + C00144-SYN	ATP + Xanthosine 5'-phosphate + Ammonia	AMP + Diphosphate + GMP	Purine,GTP	1
R01231	31	C00002 + C00655 + C00064 + C00001 <=> C00020 + C00013 + C00144-SYN + C00025	ATP + Xanthosine 5'-phosphate + L-Glutamine + H2O	AMP + Diphosphate + GMP + L-Glutamate	Purine,GTP	1
R00127	32	C00002 + C00020-SYN <=> 2 C00008-SYN	ATP + AMP	2 ADP	Purine,ATP	1
R00332	32	C00002 + C00144-SYN <=> C00008 + C00035-SYN	ATP + GMP	ADP + GDP	Purine,GTP	1
R10902	32	C00002 + C00483 + C00025 <=> C00008 + C00009 + C20926	ATP + Tyramine + L-Glutamate	ADP + Orthophosphate + gamma-Glutamyltyramine	Methanofuran	1
R05815	32	C05773 + 2 C00064 + 2 C00002 + 2 C00001 <=> C06504 + 2 C00025 + 2 C00008 + 2 C00009	Cobyrinate + 2 L-Glutamine + 2 ATP + 2 H2O	Cob(II)yrinate a,c diamide + 2 L-Glutamate + 2 ADP + 2 Orthophosphate	Cobalamin	1
R11040	33	C21069 + C20926 <=> C21070 + C00013	[5-(Aminomethyl)furan-3-yl]methyl diphosphate + gamma-Glutamyltyramine	(4-[4-[2-(gamma-L-Glutamylamino)ethyl]phenoxymethyl]furan-2-yl)methanamine + Diphosphate	Methanofuran	
R00315	33	C00008-SYN + C00227 <=> C00002-SYN + C00033	ADP + Acetyl phosphate	ATP + Acetate	Carbon fixation, WL	
R00200	33	C00008-SYN + C00074 <=> C00002-SYN + C00022	ADP + Phosphoenolpyruvate	ATP + Pyruvate	Purine,ATP	
R00330	33	C00002 + C00035-SYN <=> C00008 + C00044-SYN	ATP + GDP	ADP + GTP	Purine,GTP	1
R00430	33	C00035-SYN + C00074 <=> C00044-SYN + C00022	GDP + Phosphoenolpyruvate	GTP + Pyruvate	Purine,GTP	
R01071	34	C00002-SYN + C00119 <=> C02739 + C00013	ATP + 5-Phospho-alpha-D-ribose 1-diphosphate	1-(5-Phospho-D-ribosyl)-ATP + Diphosphate	Histidine	
R00177	34	C00002-SYN + C00073 + C00001 <=> C00009 + C00013 + C00019-SYN	ATP + L-Methionine + H2O	Orthophosphate + Diphosphate + S-Adenosyl-L-methionine	Cysteine,SAM	
R03005	34	C00002-SYN + C01185 <=> C00013 + C00857	ATP + Nicotinate D-ribonucleotide	Diphosphate + Deamino-NAD+	NAD	
R03035	34	C00002-SYN + C01134 <=> C00013 + C00882	ATP + Pantetheine 4'-phosphate	Diphosphate + Dephospho-CoA	CoA	
R05220	34	C06505 + C00002-SYN <=> C06506 + C00536	Cob(I)yrinate a,c diamide + ATP	Adenosyl cobyrinate a,c diamide + Triphosphate	Cobalamin	
R00425	34	C00044-SYN + 3 C00001 <=> C00058 + C01304 + C00013	GTP + 3 H2O	Formate + 2,5-Diamino-6-(5-phospho-D-ribosylamino)pyrimidin-4(3H)-one + Diphosphate	THF,FMN	
R07459	34	C15810 + C00002-SYN <=> C15813 + C00013	Sulfur-carrier protein + ATP	Adenylyl-[sulfur-carrier protein] + Diphosphate	Thiamine diphosphate	
RMAN10	34	C00074 + C00044-SYN <=> C22297 + C00013	PEP + GTP	Enolpyruvoyl-2-diphospho-5'-guanosine + Diphosphate	F420	
R07461	35	C15812 + C15813 + C00030 <=> C00020-SYN + C15814 + C15811 + C00028	[Enzyme]-S-sulfanylcysteine + Adenylyl-[sulfur-carrier protein] + Reduced acceptor	AMP + Thiocarboxy-[sulfur-carrier protein] + [Enzyme]-cysteine + Acceptor	Thiamine diphosphate	
R04035	35	C02739 + C00001 <=> C02741 + C00013	1-(5-Phospho-D-ribosyl)-ATP + H2O	Phosphoribosyl-AMP + Diphosphate	Histidine	1
R05225	35	C06506 + 4 C00064 + 4 C00002 + 4 C00001 <=> C06507 + 4 C00025 + 4 C00009 + 4 C00008	Adenosyl cobyrinate a,c diamide + 4 L-Glutamine + 4 ATP + 4 H2O	Adenosyl cobyrinate hexaamide + 4 L-Glutamate + 4 Orthophosphate + 4 ADP	Cobalamin	1
R11919	35	C03373 + C00019-SYN + C00030 <=> C21764 + C05198 + C00073 + C00058 + C00014 + C00009 + C00028	Aminoimidazole ribotide + S-Adenosyl-L-methionine + Reduced acceptor	5-Hydroxybenzimidazole + 5'-Deoxyadenosine + L-Methionine + Formate + Ammonia + Orthophosphate + Acceptor	Cobalamin	
R00189	35	C00002 + C00857 + C00014 <=> C00020 + C00013 + C00003-SYN	ATP + Deamino-NAD+ + Ammonia	AMP + Diphosphate + NAD+	NAD	1
R00257	35	C00002 + C00857 + C00064 + C00001 <=> C00020 + C00013 + C00003-SYN + C00025	ATP + Deamino-NAD+ + L-Glutamine + H2O	AMP + Diphosphate + NAD+ + L-Glutamate	NAD	1
R00130	35	C00002 + C00882 <=> C00008 + C00010-SYN	ATP + Dephospho-CoA	ADP + CoA	CoA	1
R11372	36	C21310 + C00001 <=> C18239 + C00013	(8S)-3',8-Cyclo-7,8-dihydroguanosine 5'-triphosphate + H2O	Precursor Z + Diphosphate	MoCo	
R04509	36	C00002 + C04556 <=> C00008 + C04752	ATP + 4-Amino-2-methyl-5-(phosphooxymethyl)pyrimidine	ADP + 4-Amino-5-hydroxymethyl-2-methylpyrimidine diphosphate	Thiamine diphosphate	1
R07302	36	C00002 + C06507 + C03194 <=> C00008 + C00009 + C06508	ATP + Adenosyl cobyrinate hexaamide + (R)-1-Aminopropan-2-ol	ADP + Orthophosphate + Adenosyl cobinamide	Cobalamin	1
R00104	36	C00002 + C00003-SYN <=> C00008 + C00006-SYN	ATP + NAD+	ADP + NADP+	NADP	1
R04621	36	C05925 + C00001 <=> C04874 + C00009	Dihydroneopterin phosphate + H2O	7,8-Dihydroneopterin + Orthophosphate	THF	
R07280	37	C04454 + C00001 <=> C04732 + C00009	5-Amino-6-(5'-phospho-D-ribitylamino)uracil + H2O	5-Amino-6-(1-D-ribitylamino)uracil + Orthophosphate	Flavin,FMN	
R05221	37	C06508 + C00002 <=> C06509 + C00008	Adenosyl cobinamide + ATP	Adenosyl cobinamide phosphate + ADP	Cobalamin	1
R06558	37	C06508 + C00044 <=> C06509 + C00035	Adenosyl cobinamide + GTP	Adenosyl cobinamide phosphate + GDP	Cobalamin	1
R09395	37	C18239 + 2 C15814 <=> C05924 + 2 C15810	Precursor Z + 2 Thiocarboxy-[sulfur-carrier protein]	Molybdopterin + 2 Sulfur-carrier protein	MoCo	
R10712	38	C04752 + C20247 <=> C01081 + C00013 + C00011	4-Amino-5-hydroxymethyl-2-methylpyrimidine diphosphate + 2-(2-Carboxy-4-methylthiazol-5-yl)ethyl phosphate	Thiamin monophosphate + Diphosphate + CO2	Thiamine diphosphate	
R04457	38	C04732 + C15556 <=> C04332 + 2 C00001 + C00009	5-Amino-6-(1-D-ribitylamino)uracil + L-3,4-Dihydroxybutan-2-one 4-phosphate	6,7-Dimethyl-8-(D-ribityl)lumazine + 2 H2O + Orthophosphate	Flavin,FMN	
R04620	38	C04895 + 3 C00001 <=> C04874 + 3 C00009	7,8-Dihydroneopterin 3'-triphosphate + 3 H2O	7,8-Dihydroneopterin + 3 Orthophosphate	THF	
R04638	38	C04895 + C00001 <=> C05925 + C00013	7,8-Dihydroneopterin 3'-triphosphate + H2O	Dihydroneopterin phosphate + Diphosphate	THF	
R05222	38	C06509 + C00044-SYN <=> C06510 + C00013	Adenosyl cobinamide phosphate + GTP	Adenosine-GDP-cobinamide + Diphosphate	Cobalamin	
R03503	38	C00002 + C01300 <=> C00020 + C04807	ATP + 6-(Hydroxymethyl)-7,8-dihydropterin	AMP + 6-Hydroxymethyl-7,8-dihydropterin diphosphate	THF	1
R09726	38	C00002-SYN + C05924 <=> C00013 + C19848	ATP + Molybdopterin	Diphosphate + Adenylated molybdopterin	MoCo	
R10339	39	C04807 + C20559 <=> C20562 + C00013	6-Hydroxymethyl-7,8-dihydropterin diphosphate + 4-(beta-D-Ribofuranosyl)aniline 5'-phosphate	N-[(7,8-Dihydropterin-6-yl)methyl]-4-(beta-D-ribofuranosyl)aniline 5'-phosphate + Diphosphate	THMPT	
R03067	39	C04807 + C00568 <=> C00013 + C00921	6-Hydroxymethyl-7,8-dihydropterin diphosphate + 4-Aminobenzoate	Diphosphate + Dihydropteroate	THF	
R09735	39	C19848 + C06232 <=> C18237 + C00020-SYN + C00001	Adenylated molybdopterin + Molybdate	Molybdoenzyme molybdenum cofactor + AMP + H2O	MoCo	
R02237	39	C00002 + C00921 + C00025 <=> C00008 + C00009 + C00415	ATP + Dihydropteroate + L-Glutamate	ADP + Orthophosphate + Dihydrofolate	THF	1
R00617	39	C00002 + C01081 <=> C00008 + C00068	ATP + Thiamin monophosphate	ADP + Thiamin diphosphate	Thiamine diphosphate	1
R00549	40	C00002 + C00255 <=> C00008 + C00061-SYN	ATP + Riboflavin	ADP + FMN	Flavin,FMN	1
R04594	40	C04778 + C00001 <=> C05775 + C00009	N1-(5-Phospho-alpha-D-ribosyl)-5,6-dimethylbenzimidazole + H2O	alpha-Ribazole + Orthophosphate	Cobalamin	
RMAN11	40	C19154 + C22297 <=> C22277 + C00144-SYN	7,8-Didemethyl-8-hydroxy-5-deazariboflavin + Enolpyruvoyl-2-diphospho-5'-guanosine	Dehydro coenzyme F420-0 + GMP	F420	
RMAN2	40	C20562 <=> C05927	N-[(7,8-Dihydropterin-6-yl)methyl]-4-(beta-D-ribofuranosyl)aniline 5'-phosphate	7,8-Dihydromethanopterin	THMPT	
R05223	41	C06510 + C05775 <=> C00194 + C00144-SYN	Adenosine-GDP-cobinamide + alpha-Ribazole	Cobamide coenzyme + GMP	Cobalamin	
R00161	41	C00002-SYN + C00061-SYN <=> C00013 + C00016	ATP + FMN	Diphosphate + FAD	Flavin,FAD	
R03013	41	C01100 + C00001 <=> C00860 + C00009	L-Histidinol phosphate + H2O	L-Histidinol + Orthophosphat	Histidine	
R09399	42	C19153 + C00044 + C00025 <=> C19152 + C00035 + C00009	Coenzyme F420-0 + GTP + L-Glutamate	Coenzyme F420-1 + GDP + Orthophosphate	F420	1
R09400	43	C19152 + C00044 + C00025 <=> C00876-SYN + C00035 + C00009	Coenzyme F420-1 + GTP + L-Glutamate	Coenzyme F420 + GDP + Orthophosphate	F420	1

Table S3. Overview of electron transfer-related reactions (Figure 2B), including Reaction ID, stratification rank, recoded reaction equation, reaction equations in compound names (left and right), pathway information, and oxidation-information.

Reaction ID	Rank	Oxidation	Recoded reaction equation	Reaction equation (Compound name) left	Reaction equation (Compound name) right	Pathway
R00134	1		C00011 + C00005 + C00080 <=>C00058 + C00006	CO2 + NADPH + H+	Formate + NADP+	Carbonfixation,WL
R01195	1		2C00138+C00006+C00080<=>2C00139+C00005	2Reducedferredoxin+NADP++H+	2 Oxidized ferredoxin + NADPH	Carbonfixation,WL
R07157	1		C00011+2C00138+2C00080<=>C00237+C00001+2C00139	CO2 + 2 Reduced ferredoxin + 2 H+	CO+H2O+2Oxidizedferredoxin	Carbonfixation,WL
R03015	1		C00862+2C00138+C00011+2C00080<=>C01001+2C00139+ C00001	Methanofuran+2Reducedferredoxin+CO2+2H+	Formylmethanofuran +2 Oxidized ferredoxin +H2O	Carbonfixation,WL
RMAN4	1		C00011 + C00282 <=> C00058 + C00080	CO2+Hydrogen	Formate + H+	Carbonfixation,WL
R00019	2		C00282+2C00139<=>2C00138+2C00080	Hydrogen+2Oxidizedferredoxin	2Reducedferredoxin+2H+	Carbonfixation,WL
R01655	3		C00234 + C00080 <=> C00445 + C00001	10-Formyltetrahydrofolate+H+	5,10-Methenyltetrahydrofolate+H2O	Carbonfixation,WL
R03464	3		C01274 + C00080 <=> C04330 + C00001	5-Formyl-5,6,7,8-tetrahydromethanopterin+H+	5,10-Methenyltetrahydromethanopterin+H2O	Carbonfixation,WL
R01220	4		C00445 + C00005 <=> C00143 + C00006	5,10-Methenyltetrahydrofolate+NADPH	5,10-Methylenetetrahydrofolate+NADP+	Carbonfixation,WL
R04456	4		C04330 + C01080 <=> C04377 + C00876 + C00080	5,10-Methenyltetrahydromethanopterin+ReducedcoenzymeF420	5,10-Methylenetetrahydromethanopterin+CoenzymeF420+H+	Carbonfixation,WL
R04455	4		C04330 + C00282 <=> C04377 + C00080	5,10-Methenyltetrahydromethanopterin+Hydrogen	5,10-Methylenetetrahydromethanopterin+H+	Carbonfixation,WL
R07168	5		C00143 + C00004 + C00080 <=>C00440 + C00003	5,10-Methylenetetrahydrofolate+NADH +H+	5-Methyltetrahydrofolate+NAD+	Carbonfixation,WL
R04464	5		C04377 + C01080 <=> C04488 + C00876	5,10-Methylenetetrahydromethanopterin+ReducedcoenzymeF420	5-Methyl-5,6,7,8-tetrahydromethanopterin+CoenzymeF420	Carbonfixation,WL
R03025	5		C00876 + C00282 <=>C01080	CoenzymeF420+Hydrogen	ReducedcoenzymeF420	Carbonfixation,WL
R09096	6		C00010+C04488+C00237<=> C00024+C01217	CoA+5-Methyl-5,6,7,8-tetrahydromethanopterin+CO	Acetyl-CoA+5,6,7,8-Tetrahydromethanopterin	Carbonfixation,WL
R10243	6		C00440 + C00010 + C00237 <=>C00101 + C00024	5-Methyltetrahydrofolate+CoA+CO	Tetrahydrofolate+Acetyl-CoA	Carbonfixation,WL
R01196	7		2C00138+C00024+C00011+2C00080<=>2C00139+C00022+C00010	2 Reduced ferredoxin + Acetyl-CoA + CO2 + 2 H+	2 Oxidized ferredoxin + Pyruvate + CoA	Carbonfixation,rTCA
R10866	7		C00024 + C00011 + C02745 <=>C00022 + C00010 + C02869	Acetyl-CoA+CO2+Reducedflavodoxin	Pyruvate+CoA+Oxidizedflavodoxin	Carbonfixation,rTCA
R00396	8		C00022 + C00014 + C00004 + C00080 <=> C00041 + C00003 + C00001	Pyruvate+Ammonia+NADH +H+	L-Alanine + NAD+ + H2O	Alanine
R00342	9		C00036 + C00004 + C00080 <=>C00149 + C00003	Oxaloacetate+NADH +H+	(S)-Malate + NAD+	Carbonfixation,rTCA
R04439	9		C06010 + C00005 + C00080 <=>C04272 + C00006	(S)-2-Acetolactate+NADPH +H+	(R)-2,3-Dihydroxy-3-methylbutanoate+NADP+	CoA
R04440	10	1	C04181 + C00005 + C00080 <=>C04272 + C00006	3-Hydroxy-3-methyl-2-oxobutanoicacid+NADPH +H+	(R)-2,3-Dihydroxy-3-methylbutanoate+NADP+	Valine,Isoleucine
R10116	11		C20372 + C00005 + C00080 <=>C20373 + C00006	3-Ketoglutaryl-[acp]methylester+NADPH +H+	3-Hydroxyglutaryl-[acp]methylester +NADP+	Biotin
R00402	11		C00122 + C00004 + C00080 <=>C00042 + C00003	Fumarate + NADH + H+	Succinate+NAD+	Carbonfixation,rTCA
R07136	11	1	C11537 + C00003 <=> C05528 + C00004 + C00080	(2R)-3-Sulfolactate+NAD+	3-Sulfopyruvate+NADH +H+	CoM
R00994	11	1	C06032 + C00003 <=>C00109 + C00011 + C00004 + C00080	D-erythro-3-Methylmalate+NAD+	2-Oxobutanoate+CO2+NADH +H+	Isoleucine
R01513	11	1	C00197 + C00003 <=> C03232 + C00004 + C00080	3-Phospho-D-glycerate+NAD+	3-Phosphonooxypyruvate+NADH +H+	Serine
R01061	12		C00236 + C00004 + C00080 <=> C00118 + C00009 + C00003	3-Phospho-D-glyceroylphosphate+NADH +H+	D-Glyceraldehyde3-phosphate+Orthophosphate+NAD+	Glycolysis/gluconeogenesis
R01063	12		C00236 + C00005 + C00080 <=> C00118 + C00009 + C00006	3-Phospho-D-glyceroylphosphate+NADPH +H+	D-Glyceraldehyde3-phosphate+Orthophosphate+NADP+	Glycolysis/gluconeogenesis
R02472	12		C00966 + C00005 + C00080 <=>C00522 + C00006	2-Dehydropantoate+NADPH +H+	(R)-Pantoate + NADP+	CoA
R10118	13		C20374 + C00005 + C00080 <=>C20375 + C00006	Enoylglutaryl-[acp]methylester+NADPH +H+	Glutaryl-[acp]methyl ester +NADP+	Biotin
R01197	13		2C00138+C00091+C00011+2C00080<=>2C00139+C00026+C00010	2 Reduced ferredoxin + Succinyl-CoA + CO2 + 2 H+	2Oxidizedferredoxin+2-Oxoglutarate+CoA	Carbonfixation,rTCA
R07159	13		C00118+C00001+2C00139<=>C00197+2C00080+2C00138	D-Glyceraldehyde3-phosphate+H2O+2Oxidizedferredoxin	3-Phospho-D-glycerate+2H++2Reducedferredoxin	Glycolysis/gluconeogenesis
R01058	13	1	C00118 + C00006 + C00001 <=>C00197 + C00005 + C00080	D-Glyceraldehyde3-phosphate+NADP++H2O	3-Phospho-D-glycerate+NADPH +H+	Glycolysis/gluconeogenesis
R09153	13		C00593 <=> C03576	[Protein]-S-sulfanyl-L-cysteine+Sulfoacetaldehyde+4Reducedferredoxin+4H+	[Protein]-L-cysteine+CoenzymeM +4Oxidizedferredoxin	CoM
R00267	14		C00026 + C00011 + C00005 + C00080 <=> C00311 + C00006	2-Oxoglutarate+CO2+NADPH +H+	Isocitrate+NADP+	Carbonfixation,rTCA
R00243	14		C00026 + C00014 + C00004 + C00080 <=> C00025 + C00003 + C00001	2-Oxoglutarate+Ammonia+NADH +H+	L-Glutamate + NAD+ + H2O	Glutamate
R00248	14		C00026 + C00014 + C00005 + C00080 <=> C00025 + C00006 + C00001	2-Oxoglutarate+Ammonia+NADPH +H+	L-Glutamate + NADP+ + H2O	Glutamate
R05068	14		C14463 + C00005 + C00080 <=>C06007 + C00006	(R)-3-Hydroxy-3-methyl-2-oxopentanoate+NADPH +H+	(R)-2,3-Dihydroxy-3-methylpentanoate+NADP+	Isoleucine
R04426	14	1	C04411 + C00003 <=> C04236 + C00004 + C00080	(2R,3S)-3-Isopropylmalate+NAD+	(2S)-2-Isopropyl-3-oxosuccinate+NADH +H+	Leucine
R10120	15		C20376 + C00005 + C00080 <=>C20377 + C00006	3-Ketopimeloyl-[acp]methylester+NADPH +H+	3-Hydroxypimeloyl-[acp]methylester +NADP+	Biotin
RMAN5	15		C00025 <=> C03741	L-Glutamate	(S)-4-Amino-5-oxopentanoate	F430, Cobalamin
R03313	16		C03287 + C00005 + C00080 <=> C01165 + C00009 + C00006	L-Glutamyl5-phosphate+NADPH +H+	L-Glutamate5-semialdehyde+Orthophosphate+NADP+	Proline
R01088	16		C00233 + C00014 + C00004 + C00080 <=> C00123 + C00001 + C00003	4-Methyl-2-oxopentanoate+Ammonia+NADH +H+	L-Leucine+H2O+NAD+	Leucine
R07407	16	1	C00049 + C00006 <=> C05840 + C00005 + C00080	L-Aspartate+NADP+	Iminoaspartate+NADPH +H+	NAD
R07410	16	1	C00049 + C00003 <=> C05840 + C00004 + C00080	L-Aspartate+NAD+	Iminoaspartate+NADH +H+	NAD
R03443	17		C04133 + C00005 + C00080 <=>C01250 + C00009 + C00006	N-Acetyl-L-glutamate5-phosphate+NADPH +H+	N-Acetyl-L-glutamate5-semialdehyde+Orthophosphate+NADP+	Arginine
R02291	17		C03082 + C00005 + C00080 <=>C00441 + C00009 + C00006	4-Phospho-L-aspartate+NADPH +H+	L-Aspartate4-semialdehyde+Orthophosphate+NADP+	Lysine,Threonine,Methionine
R10122	17		C20378 + C00005 + C00080 <=>C19846 + C00006	Enoylpimeloyl-[acp]methylester+NADPH +H+	Pimeloyl-[acyl-carrierprotein]methyl ester+NADP+	Biotin
R01934	17	1	C05662 + C00003 <=> C00322 + C00011 + C00004 + C00080	Homoisocitrate+NAD+	2-Oxoadipate+CO2+NADH +H+	Lysine
R01773	18		C00441 + C00004 + C00080 <=>C00263 + C00003	L-Aspartate4-semialdehyde+NADH +H+	L-Homoserine+NAD+	Methionine,Threonine
R01775	18		C00441 + C00005 + C00080 <=>C00263 + C00006	L-Aspartate4-semialdehyde+NADPH +H+	L-Homoserine+NADP+	Methionine,Threonine
R02736	18	1	C01172 + C00006 <=> C01236 + C00005 + C00080	beta-D-Glucose6-phosphate+NADP+	D-Glucono-1,5-lactone6-phosphate+NADPH +H+	Pentosephosphatepathway
R10907	18	1	C01172 + C00003 <=> C01236 + C00004 + C00080	beta-D-Glucose6-phosphate+NAD+	D-Glucono-1,5-lactone6-phosphate+NADH +H+	Pentosephosphatepathway
R01248	18		C03912 + C00004 + C00080 <=>C00148 + C00003	(S)-1-Pyrroline-5-carboxylate+NADH +H+	L-Proline + NAD+	Proline
R01251	18		C03912 + C00005 + C00080 <=>C00148 + C00006	(S)-1-Pyrroline-5-carboxylate+NADPH +H+	L-Proline + NADP+	Proline
R01869	18	1	C00337 + C00003 <=> C00295 + C00080 + C00004	(S)-Dihydroorotate+NAD+	Orotate + H+ + NADH	Pyrimidine
R04198	19		C20258 + C00004 + C00080 <=>C03972 + C00003 + C00001	(2S,4S)-4-Hydroxy-2,3,4,5-tetrahydrodipicolinate+NADH +H+	2,3,4,5-Tetrahydrodipicolinate+NAD++H2O	Lysine
R04199	19		C20258 + C00005 + C00080 <=>C03972 + C00006 + C00001	(2S,4S)-4-Hydroxy-2,3,4,5-tetrahydrodipicolinate+NADPH +H+	2,3,4,5-Tetrahydrodipicolinate+NADP++H2O	Lysine
R03759	20		C01888 + C00004 + C00080 <=>C03194 + C00003	Aminoacetone+NADH +H+	(R)-1-Aminopropan-2-ol + NAD+	Glycine
R01528	20	1	C00345 + C00006 <=>C00199 + C00011 + C00005 + C00080	6-Phospho-D-gluconate+NADP+	D-Ribulose5-phosphate+CO2+NADPH +H+	Pentosephosphatepathway
R10221	20	1	C00345 + C00003 <=>C00199 + C00011 + C00004 + C00080	6-Phospho-D-gluconate+NAD+	D-Ribulose5-phosphate+CO2+NADH +H+	Pentosephosphatepathway
R03947	21	1	C02463 + C00003 <=> C05778 + C00004 + C00080	Precorrin 2 + NAD+	Sirohydrochlorin+NADH +H+	F430,Cobalamin
R01465	21	1	C00188 + C00003 <=> C03508 + C00004 + C00080	L-Threonine + NAD+	L-2-Amino-3-oxobutanoicacid+NADH +H+	Glycine
R02755	21		C03871 + C00014 + C00005 + C00080 <=> C00680 + C00006 + C00001	L-2-Amino-6-oxoheptanedioate+Ammonia+NADPH +H+	meso-2,6-Diaminoheptanedioate+NADP++H2O	Lysine
R08214	21	1	C16597 + C00003 <=>C16588 + C00011 + C00004 + C00080	(-)-threo-Iso(homo)2-citrate+NAD+	2-Oxopimelate+CO2+NADH +H+	CoB
R02413	24		C02637 + C00005 + C00080 <=>C00493 + C00006	3-Dehydroshikimate+NADPH +H+	Shikimate+NADP+	Tryptothan,Phenylalanine,Tyrosine
R08215	25	1	C16600 + C00003 <=>C16589 + C00011 + C00004 + C00080	(-)-threo-Iso(homo)3-citrate+NAD+	2-Oxosuberate+CO2+NADH +H+	CoB
R11636	25		C00063-SYN + C00058 <=> C00458 + C00011 + C00001	CTP + Formate	dCTP + CO2 + H2O	Pyrimidine
R05812	28		C11542 + C00004 + C00080 <=> C11543 + C00003	Cobalt-precorrin6+NADH +H+	Cobalt-dihydro-precorrin 6 + NAD+	Cobalamin
R00936	29		C00415 + C00004 + C00080 <=>C00101-SYN + C00003	Dihydrofolate+NADH +H+	Tetrahydrofolate+NAD+	THF
R00939	29		C00415 + C00005 + C00080 <=>C00101-SYN + C00006	Dihydrofolate+NADPH +H+	Tetrahydrofolate+NADP+	THF
R01728	29		C00254 + C00003 <=>C01179 + C00011 + C00004 + C00080	Prephenate+ NAD+	3-(4-Hydroxyphenyl)pyruvate+CO2+NADH +H+	Tyrosine
R01730	29	1	C00254 + C00006 <=>C01179 + C00011 + C00005 + C00080	Prephenate+ NADP+	3-(4-Hydroxyphenyl)pyruvate+CO2+NADPH +H+	Tyrosine
R01130	30	1	C00130 + C00003 + C00001 <=>C00655 + C00004 + C00080	IMP + NAD+ + H2O	Xanthosine5'-phosphate+NADH +H+	Purine,GTP
R00732	30	1	C00826 + C00003 <=>C00082 + C00011 + C00004 + C00080	L-Arogenate+ NAD+	L-Tyrosine+CO2+NADH +H+	Tyrosine
R00733	30	1	C00826 + C00006 <=>C00082 + C00011 + C00005 + C00080	L-Arogenate+ NADP+	L-Tyrosine+CO2+NADPH +H+	Tyrosine
RMAN1	30		C21107 <=> C20559	4-(beta-D-Ribofuranosyl)phenol 5'-phosphate	4-(beta-D-Ribofuranosyl)aniline5'-phosphate	THMPT
R05218	33		2C06504+C01847<=>2C06505+C00061+2C00080	2 Cob(II)yrinate a,c diamide + Reduced FMN	2 Cob(I)yrinate a,c diamide + FMN + 2 H+	Cobalamin
R05705	34		C00061 + C00004 + C00080 <=>C01847 + C00003	FMN + NADH + H+	ReducedFMN +NAD+	Cobalamin
R05706	34		C00061 + C00005 + C00080 <=>C01847 + C00006	FMN + NADPH + H+	ReducedFMN +NADP+	Cobalamin
R11633	34		C00044-SYN + C00058 <=> C00286 + C00011 + C00001	GTP + Formate	dGTP + CO2 + H2O	Purine
R11634	34		C00002-SYN + C00058 <=> C00131 + C00011 + C00001	ATP + Formate	dATP + CO2+ H2O	Purine
R01078	35		C01909+C17023+2C00019-SYN +2C05359+2C00080<=>C00120+ 2C00073+2C05198	Dethiobiotin+Sulfur donor +2S-Adenosyl-L-methionine+2e- +2H+	Biotin+2L-Methionine+25'-Deoxyadenosine	Biotin
R11580	35		C00019-SYN + C17401 + C00030 <=> C00021-SYN+ C11540 + C00028	S-Adenosyl-L-methionine+Cobalt-factor III+Reducedacceptor	S-Adenosyl-L-homocysteine+Cobalt-precorrin4+Acceptor	Cobalamin
R09394	35		C00044-SYN + C00019-SYN + C00030 <=> C21310 + C05198 + C00073 + C00028	GTP+S-Adenosyl-L-methionine+Reducedacceptor	(8S)-3',8-Cyclo-7,8-dihydroguanosine5'-triphosphate+5'-Deoxyadenosine+L-Methionine+Acceptor	MoCo
R11919	35		C03373 + C00019-SYN + C00030 <=> C21764 + C05198 + C00073 + C00058 + C00014 + C00009 + C00028	Aminoimidazoleribotide+S-Adenosyl-L-methionine+Reducedacceptor	5-Hydroxybenzimidazole+5'-Deoxyadenosine+L-Methionine+Formate+Ammonia+Orthophosphate+Acceptor	Cobalamin
R03458	36		C01268 + C00005 + C00080 <=>C04454 + C00006	5-Amino-6-(5'-phosphoribosylamino)uracil+NADPH +H+	5-Amino-6-(5'-phospho-D-ribitylamino)uracil+NADP+	Flavin,FMN
R10802	41		C05927 + C00030 <=> C01217-SYN + C00028	7,8-Dihydromethanopterin+Reducedacceptor	5,6,7,8-Tetrahydromethanopterin+Acceptor	THMPT
RMAN12	41		C22277 + C01847 <=> C19153 + C00061 + C00080	DehydrocoenzymeF420-0+ReducedFMN	CoenzymeF420-0+FMN +H+	F420
R03388	41		C05927 + C00080 + C00005 <=>C01217-SYN + C00006	7,8-Dihydromethanopterin+H++NADPH	5,6,7,8-Tetrahydromethanopterin+NADP+	THMPT
R03012	42	1	C00860 + C00003 <=> C01929 + C00004 + C00080	L-Histidinol+NAD+	L-Histidinal+NADH +H+	Histidine
R01163	43	1	C01929 + C00001 + C00003 <=>C00135 + C00004 + C00080	L-Histidinal+H2O+NAD+	L-Histidine+NADH +H+	Histidine

Table S4. Overview of thioester-related reactions (Figure 2C), including Reaction ID, stratification rank, recoded reaction equation, reaction equations in compound names (left and right), pathway information, and thioester-formation-information.

Reaction ID	Rank	Formation	Recoded reaction equation	Reaction equation (Compound name) left	Reaction equation (Compound name) right	Pathway
R10243	6	1	C00440 + C00010 + C00237 <=> C00101 + C00024	5-Methyltetrahydrofolate +CoA +CO	Tetrahydrofolate +Acetyl-CoA	Carbon fixation, WL
R09096	6	1	C00010 + C04488 + C00237 <=> C00024 + C01217	CoA +5-Methyl-5,6,7,8-tetrahydromethanopterin +CO	Acetyl-CoA +5,6,7,8-Tetrahydromethanopterin	Carbon fixation, WL
R00742	7		C00002 + C00024 + C00288 <=> C00008 + C00009 + C00083	ATP +Acetyl-CoA + HCO3-	ADP +Orthophosphate +Malonyl-CoA	Carbon fixation
R00230	7		C00024 + C00009 <=> C00010 + C00227	Acetyl-CoA +Orthophosphate	CoA +Acetylphosphate	Carbon fixation, WL
R01196	7		2 C00138 +C00024 +C00011 + 2 C00080 <=>2 C00139 +C00022 + C00010	2 Reduced ferredoxin +Acetyl-CoA + CO2 +2 H+	2 Oxidized ferredoxin +Pyruvate +CoA	Carbon fixation, rTCA
R10866	7		C00024 + C00011 + C02745 <=> C00022 + C00010 + C02869	Acetyl-CoA +CO2 +Reduced flavodoxin	Pyruvate +CoA +Oxidized flavodoxin	Carbon fixation, rTCA
R01626	8		C00083 + C00229 <=> C00010 + C01209	Malonyl-CoA +Acyl-carrier protein	CoA +Malonyl-[acyl-carrier protein]	Biotin
R07399	8		C00024 + C00022 + C00001 <=> C02612 + C00010	Acetyl-CoA +Pyruvate +H2O	(R)-2-Methylmalate +CoA	Isoleucine
R10115	10		C19673 + C01209 <=> C20372 + C00011 + C00229	Malonyl-[acp] methylester +Malonyl-[acyl-carrier protein]	3-Ketoglutaryl-[acp] methylester +CO2 + Acyl-carrier protein	Biotin
R01213	11		C00024 + C00141 + C00001 <=> C02504 + C00010	Acetyl-CoA +3-Methyl-2-oxobutanoic acid +H2O	alpha-Isopropylmalate +CoA	Leucine
R10116	11		C20372 + C00005 + C00080 <=> C20373 + C00006	3-Ketoglutaryl-[acp] methylester +NADPH +H+	3-Hydroxyglutaryl-[acp] methylester +NADP+	Biotin
R00405	12	1	C00002 + C00042 + C00010 <=> C00008 + C00009 + C00091	ATP +Succinate +CoA	ADP +Orthophosphate +Succinyl-CoA	Carbon fixation, rTCA
R10117	12		C20373 <=> C20374 + C00001	3-Hydroxyglutaryl-[acp] methylester	Enoylglutaryl-[acp] methylester +H2O	Biotin
R10118	13		C20374 + C00005 + C00080 <=> C20375 + C00006	Enoylglutaryl-[acp] methylester +NADPH +H+	Glutaryl-[acp] methylester +NADP+	Biotin
R01197	13		2 C00138 +C00091 +C00011 + 2 C00080 <=>2 C00139 +C00026 + C00010	2 Reduced ferredoxin +Succinyl-CoA + CO2 +2 H+	2 Oxidized ferredoxin +2-Oxoglutarate +CoA	Carbon fixation, rTCA
R10119	14		C20375 + C01209 <=> C20376 + C00011 + C00229	Glutaryl-[acp] methylester +Malonyl-[acyl-carrier protein]	3-Ketopimeloyl-[acp] methylester +CO2 + Acyl-carrier protein	Biotin
R00271	14		C00024 + C00001 + C00026 <=> C01251 + C00010	Acetyl-CoA +H2O +2-Oxoglutarate	(R)-2-Hydroxybutane-1,2,4-tricarboxylate +CoA	Lysine
R00259	15		C00024 + C00025 <=> C00010 + C00624	Acetyl-CoA +L-Glutamate	CoA +N-Acetyl-L-glutamate	Arginine
R10120	15		C20376 + C00005 + C00080 <=> C20377 + C00006	3-Ketopimeloyl-[acp] methylester +NADPH +H+	3-Hydroxypimeloyl-[acp] methylester +NADP+	Biotin
R10121	16		C20377 <=> C20378 + C00001	3-Hydroxypimeloyl-[acp] methylester	Enoylpimeloyl-[acp] methylester +H2O	Biotin
R00352	17	1	C00002 + C00158 + C00010 <=> C00008 + C00009 + C00024 + C00036	ATP +Citrate +CoA	ADP +Orthophosphate +Acetyl-CoA +Oxaloacetate	Carbon fixation, rTCA
R00586	17		C00065 + C00024 <=> C00979 + C00010	L-Serine +Acetyl-CoA	O-Acetyl-L-serine +CoA	Cysteine
R01322	17	1	C00002 + C00158 + C00010 <=> C00008 + C00009 + C00566	ATP +Citrate +CoA	ADP +Orthophosphate +(3S)-Citryl-CoA	Carbon fixation, rTCA
R10122	17		C20378 + C00005 + C00080 <=> C19846 + C00006	Enoylpimeloyl-[acp] methylester +NADPH +H+	Pimeloyl-[acyl-carrier protein] methylester +NADP+	Biotin
R00354	18		C00566 <=> C00024 + C00036	(3S)-Citryl-CoA	Acetyl-CoA +Oxaloacetate	Carbon fixation, rTCA
R00371	18		C00024 + C00037 <=> C00010 + C03508	Acetyl-CoA +Glycine	CoA +L-2-Amino-3-oxobutanoic acid	Glycine
R08331	18		C00322 + C00024 + C00001 <=> C16583 + C00010	2-Oxoadipate +Acetyl-CoA +H2O	(R)-(Homo)2-citrate +CoA	CoB
R01777	19		C00091 +C00263 <=>C00010 + C01118	Succinyl-CoA +L-Homoserine	CoA +O-Succinyl-L-homoserine	Methionine
R04364	20		C03972 + C00024 + C00001 <=> C05539 + C00010	2,3,4,5-Tetrahydrodipicolinate +Acetyl-CoA +H2O	N-Acetyl-L-2-amino-6-oxopimelate +CoA	Lysine
R04365	20		C00091 + C03972 + C00001 <=> C00010 + C04462	Succinyl-CoA +2,3,4,5-Tetrahydrodipicolinate +H2O	CoA +N-Succinyl-2-L-amino-6-oxoheptanedioate	Lysine
R08332	22		C16588 + C00024 + C00001 <=> C16598 + C00010	2-Oxopimelate +Acetyl-CoA +H2O	(R)-(Homo)3-citrate +CoA	CoB
R09543	35		C00019-SYN + C01209 <=> C00021-SYN + C19673	S-Adenosyl-L-methionine +Malonyl-[acyl-carrier protein]	S-Adenosyl-L-homocysteine +Malonyl-[acp] methylester	Biotin
